# Metabolic Profile and Long-Term Risk of Depression, Anxiety, and Stress-Related Disorders

**DOI:** 10.1001/jamanetworkopen.2024.4525

**Published:** 2024-04-02

**Authors:** Charilaos Chourpiliadis, Yu Zeng, Anikó Lovik, Dang Wei, Unnur Valdimarsdóttir, Huan Song, Niklas Hammar, Fang Fang

**Affiliations:** 1 Institute of Environmental Medicine, Karolinska Institutet, Stockholm, Sweden; 2 West China Biomedical Big Data Center, West China Hospital, Sichuan University, Chengdu, China; 3 Institute of Psychology, Leiden University, Leiden, the Netherlands; 4 Center of Public Health Sciences, University of Iceland, Reykjavik, Iceland; 5 Department of Epidemiology, Harvard T.H. Chan School of Public Health, Boston, Massachusetts

## Abstract

**Importance:**

Biomarkers of lipid, apolipoprotein, and carbohydrate metabolism have been previously suggested to be associated with the risk for depression, anxiety, and stress-related disorders, but results are inconsistent.

**Objective:**

To examine whether the biomarkers of carbohydrate, lipid, and apolipoprotein metabolism are associated with the risk of depression, anxiety, and stress-related disorders.

**Design, Setting, and Participants:**

This population-based cohort study with longitudinal data collection assessed 211 200 participants from the Apolipoprotein-Related Mortality Risk (AMORIS) cohort who underwent occupational health screening between January 1, 1985, and December 31, 1996, mainly in the Stockholm region in Sweden. Statistical analysis was performed during 2022 to 2023.

**Exposures:**

Lipid, apolipoprotein, and carbohydrate biomarkers measured in blood.

**Main Outcomes and Measures:**

The associations between biomarker levels and the risk of developing depression, anxiety, and stress-related disorders through the end of 2020 were examined using Cox proportional hazards regression models. In addition, nested case-control analyses were conducted within the cohort, including all incident cases of depression, anxiety, and stress-related disorders, and up to 10 control individuals per case who were individually matched to the case by year of birth, sex, and year of enrollment to the AMORIS cohort, using incidence density sampling. Population trajectories were used to illustrate the temporal trends in biomarker levels for cases and controls.

**Results:**

A total of 211 200 individuals (mean [SD] age at first biomarker measurement, 42.1 [12.6] years; 122 535 [58.0%] male; 188 895 [89.4%] born in Sweden) participated in the study. During a mean (SD) follow-up of 21.0 (6.7) years, a total of 16 256 individuals were diagnosed with depression, anxiety, or stress-related disorders. High levels of glucose (hazard ratio [HR], 1.30; 95% CI, 1.20-1.41) and triglycerides (HR, 1.15; 95% CI, 1.10-1.20) were associated with an increased subsequent risk of all tested psychiatric disorders, whereas high levels of high-density lipoprotein (HR, 0.88; 95% CI, 0.80-0.97) were associated with a reduced risk. These results were similar for male and female participants as well as for all tested disorders. The nested case-control analyses demonstrated that patients with depression, anxiety, or stress-related disorders had higher levels of glucose, triglycerides, and total cholesterol during the 20 years preceding diagnosis, as well as higher levels of apolipoprotein A-I and apolipoprotein B during the 10 years preceding diagnosis, compared with control participants.

**Conclusions and Relevance:**

In this cohort study of more than 200 000 participants, high levels of glucose and triglycerides and low levels of high-density lipoprotein were associated with future risk of depression, anxiety, and stress-related disorders. These findings may support closer follow-up of individuals with metabolic dysregulations for the prevention and diagnosis of psychiatric disorders.

## Key Points

**Question:**

Are the biomarkers of carbohydrate, lipid, and apolipoprotein metabolism associated with the risk of depression, anxiety, and stress-related disorders?

**Findings:**

In this population-based cohort study of more than 200 000 individuals, high levels of glucose and triglycerides and a low level of high-density lipoprotein were associated with a higher future risk of depression, anxiety, and stress-related disorders.

**Meaning:**

These findings suggest that carbohydrate and lipid metabolism may be involved in the development of common psychiatric disorders.

## Teaser

This cohort study examines whether metabolic biomarkers of carbohydrate, lipid, and apolipoprotein are associated with the development of common psychiatric disorders.

## Introduction

Depression, anxiety, and stress-related disorders are common psychiatric disorders, affecting approximately one-third of individuals during the life course.^1^ Increasing evidence suggests that metabolic dysregulation may contribute to the development of psychiatric disorders.^2,3,4^ Inflammation may play an important role in the link between metabolic dysregulation and psychiatric disorders. Lipid and glucose abnormalities may activate innate immune cells,^5,6,7,8^ promote the release of proinflammatory cytokines and biogenic amines catabolism,^9,10^ and consequently lead to microglia-induced hypothalamic inflammation,^11^ which have all been linked to the development of depression, anxiety, and stress-related disorders. Furthermore, adipokines, saturated fatty acids in plasma, and gut-signal molecules might increase the permeability of the blood-brain barrier, resulting in an elevated risk of chronic inflammation in the brain and eventually a higher risk of psychiatric disorders.^12^

The association between glucose or lipid biomarkers and the risk of depression has been investigated, but the results remain inconsistent. Although several studies found a positive association between glucose level and risk of depression,^13,14,15,16,17^ Golden et al^18^ found a negative association, whereas Vogelzangs et al^19^ found a null association. Similarly, among lipid biomarkers, a positive association was shown for triglycerides (TG), whereas a negative association was shown for high-density lipoprotein cholesterol (HDL-C), low-density lipoprotein cholesterol (LDL-C), and total cholesterol (TC) in some studies^13,14,15,20,21,22^ but not others.^19,20,23,24^ The heterogeneity of previous findings may partly result from methodologic shortcomings (eg, short follow-up^15,17,19,20,21,22,24^ and small sample size^13,15,16,17,19,20,22,23,24^). Furthermore, most of the studies investigated older individuals,^17,19,20,21,22,23,24,25,26,27,28,29^ with only a few exploring younger individuals.^14,30,31,32^ Depression was mostly ascertained using self-reported scales in previous studies,^15,17,18,19,20,23,24^ which are likely more prone to misclassification than structured clinical interviews.^33,34^ In contrast to depression, few studies have explored the associations between the carbohydrate and lipid biomarkers with anxiety and stress-related disorders, apart from the studies that investigated the associations for hyperlipidemia^32^ and diabetes type 1^35^ and type 2.^30,36^ Finally, no longitudinal studies have, to our best knowledge, explored the associations of apolipoprotein A-I (ApoA-I) and apolipoprotein B (ApoB) with the risk of anxiety or stress-related disorders. Therefore, we aimed to explore the associations of blood carbohydrate, lipid, and apolipoprotein biomarkers with the risk of depression, anxiety, and stress-related disorders in a large-scale population-based cohort with longitudinal data collection. Metabolic biomarker levels fluctuate over time^37,38^ and might be influenced by different health conditions, including psychiatric disorders. To better demonstrate the temporal association between these biomarkers and the studied psychiatric disorders, we conducted 2 complementary analyses. First, we performed a time-to-event analysis using the baseline measurement of the biomarkers as the exposure of interest to assess its association with the risk of depression, anxiety, and stress-related disorders. The baseline measurement is the furthest away from the diagnosis of psychiatric disorders and therefore is least likely to be affected by the development of a disorder. We also performed a nested case-control study to demonstrate the longitudinal trajectories of these biomarkers during the 30 years before a diagnosis of these disorders.

## Methods

### Study Population

The Swedish Apolipoprotein-Related Mortality Risk (AMORIS) cohort includes 812 073 individuals (49% men and 51% women) with laboratory analyses of blood and urine samples from January 1, 1985, to December 31, 1996 (recruitment period), mainly in the Stockholm region of Sweden. The individuals participating in the AMORIS cohort were either healthy individuals referred for laboratory investigation for routine health screening in the occupational setting or individuals with health conditions (indication) referred for laboratory testing by physicians in outpatient care. In the current cohort study, we included a total of 211 200 participants of the AMORIS cohort who were 16 years or older, had at least 1 routine health screening in the occupational setting during the recruitment period, were free of any mental disorder at baseline, and had at least 1 measurement for the studied biomarkers (eFigure 1 in Supplement 1). History of mental disorders was ascertained through the Swedish Patient Register, which includes information on psychiatric inpatient care since 1973 and on specialized outpatient care since 2001.^39^ We used the Swedish versions of the *International Classification of Diseases, Eighth Revision* (*ICD-8*) (codes 290-315), *International Classification of Diseases, Ninth Revision* (*ICD-9*) (codes 290-319), and *International Statistical Classification of Diseases and Related Health Problems, Tenth Revision* (*ICD-10*) (codes F00-F99) to identify any mental disorder. A history of chronic diseases diagnosed before the first biomarker measurement, including cardiovascular disease and cancer, was found in 367 participants (0.2%). A total of 248 individuals (67.6%) with a history of chronic diseases had a score of 1, 71 (19.4%) had a score of 2, and 48 (13%) had a score of 3 or higher on the Charlson Comorbidity Index.^40^ The study was approved by the Swedish Ethical Review Authority with the requirement for informed consent waived due to the large size of the study population and the substantial time elapsed since the baseline health examination. This study followed the Strengthening the Reporting of Observational Studies in Epidemiology (STROBE) reporting guideline.

### Outcome Ascertainment

The primary outcome was a first diagnosis of depression, anxiety, or stress-related disorders after recruitment to the AMORIS cohort, according to the Swedish Patient Register. In the definition of stress-related disorders, we included acute stress reaction, posttraumatic stress disorder, adjustment disorders, other reactions to severe stress, and unspecified reaction to severe stress. We considered both primary and secondary diagnoses as recorded in the Swedish Patient Register in ascertainment of the outcomes. As secondary outcomes, we considered the first diagnosis of each of these disorders separately. The *ICD-8*, *ICD-9*, and *ICD-10* codes used for the outcome ascertainment can be found in eTable 1 in Supplement 1. The validity of the Swedish National Patient Register is generally quite high, with a positive predictive value of approximately 85% to 95% for most of the diagnoses linked with hospitalization (inpatient diagnoses).^39^

### Biomarkers of Interest

We studied glucose, TC, HDL-C, LDL-C, TGs, the ratio of LDL-C to HDL-C, ApoA-I, ApoB, and the ratio of ApoB to ApoA-I. All laboratory analyses were performed on fresh blood samples by the same laboratory (Central Automation Laboratory, Stockholm, Sweden), using well-documented and consistently implemented methods.^41^ The concentrations of LDL-C were calculated using the Friedewald formula. The concentrations of HDL-C were calculated from the concentrations of TC, TGs, and ApoA-I in 185 466 cohort participants (84.5%) and measured directly in blood in 33 951 participants (15.5%).^42^ Enzymatic methods were implemented to calculate the plasma concentrations of TC and TGs and the serum concentration of glucose. Apolipoproteins were measured with immunoturbidimetry.^43,44^

### Covariates

Information on sex, age at blood measurement, and fasting status at blood measurement (overnight fasting: yes or no) was obtained from the AMORIS cohort. Information on socioeconomic status and country of birth (Sweden, other Nordic countries, other countries, or missing) was obtained from the Swedish Censuses in 1980, 1985, and 1990 and the Longitudinal Integrated Database for Health Insurance and Labor Market Studies (LISA) from 1990 onward. Socioeconomic status was classified as low (unskilled workers, skilled workers, and lower employees), high (intermediate employees, higher employees, and business owners), or unknown.

### Statistical Analysis

#### Cohort Study

We followed up study participants from the first measurement of studied biomarkers until the first diagnosis of depression, anxiety, or stress-related disorders, emigration, death, or the end of follow-up (December 31, 2020), whichever occurred first, through cross-linkage to the Swedish Patient Register and Total Population Register (ie, information on emigration and death). We applied Cox proportional hazards regression models to estimate hazard ratios (HRs) with 95% CIs of depression, anxiety, or stress-related disorders in relation to the level of first biomarker measurement. The proportionality assumption was graphically examined using scaled Schoenfeld residuals, without identifying major violations. The attained age was used as the underlying time scale, and the date of birth was used as the time origin. Additionally, we disregarded the first 5 years of follow-up from the analysis reduce the risk of confounding. We adjusted for sex, age at the first biomarker measurement, fasting status, socioeconomic status, and country of birth in the main model. The covariates selected have been previously shown to be associated with metabolic biomarkers, psychiatric disorders, or both.^45,46,47^

The biomarkers were used as categorical variables based on clinical cutoffs, namely, 6.11 mmol/L for glucose (to convert to milligrams per deciliter, divide by 0.0555), 5 mmol/L for TC (to convert to milligrams per deciliter, divide by 0.0259), 1.71 mmol/L for TGs (to convert to milligrams per deciliter, divide by 0.0113), 3 mmol/L for LDL-C (to convert to milligrams per deciliter, divide by 0.0259), 1.03 mmol/L for HDL-C (to convert to milligrams per deciliter, divide by 0.0259), 3.5 for LDL-C/HDL-C ratio, 1.0 mmol/L in men and 1.1 mmol/L in women for ApoA-I (to convert to milligrams per deciliter, multiply by 0.01), 0.9 mmol/L for ApoB (to convert to milligrams per deciliter, multiply by 0.01), and 0.9 in men and 0.8 in women for ApoB/ApoA-I ratio, according to previous publications and guidelines for the management of cardiovascular disease.^48,49^ Additionally, we treated the biomarkers as continuous variables to estimate the association between 1-SD increase of the biomarkers and the risk of depression, anxiety, or stress-related disorders. Stratified analyses by sex were performed to explore sex-specific results.

We performed the following sensitivity analyses to assess the robustness of the main findings. First, because some individuals in the main analysis were not employed at the time of screening, although their health screening was covered as an employment benefit, we performed a sensitivity analysis including only 161 237 participants who were 16 to 65 years old and employed at the time of screening, according to data in the closest Swedish Census or LISA. Furthermore, to examine whether the findings would pertain in biomarkers measured in relation to referral by physicians in outpatient care, we performed another sensitivity analysis using biomarker measurements in relation to referral from outpatient care. Finally, we performed a sensitivity analysis excluding the individuals with missing socioeconomic status from the analysis.

#### Nested Case-Control Study

We additionally conducted a nested case-control study within the above cohort study. All the incident cases of depression, anxiety, or stress-related disorders, combined and individually, were included as cases. At most, 10 randomly selected control individuals were individually matched to each case by sex, age, and calendar year of enrollment to the AMORIS cohort, using incidence density sampling with replacement. The date of diagnosis of the cases was defined as the index date for the case and their matched controls. Study participants who were free of the outcome before and at the index date were eligible as controls. All available measurements of the studied biomarkers before the index date were analyzed to demonstrate the temporal pattern of these biomarkers during the 30 years before index date. Descriptive statistics on the matching variables for cases and controls are reported in eTable 2 in Supplement 1. The method of local polynomial smoothing with fourth-degree polynomial function and gaussian kernel function was used to plot the mean concentrations of the biomarkers over time before the index date with 95% CIs.

All the analyses were conducted as complete case analyses because the covariates either did not contain any missing value (age at first blood sampling and sex) or contained less than 0.1% missingness (country of birth and fasting status). The missingness level was 12% for socioeconomic status, so those with missing socioeconomic status were included as an additional category (missing) in the analysis. The statistical analysis was performed during 2022 to 2023. All analyses were conducted using Stata software, version 16 (StataCorp).

## Results

Among the 211 200 study participants, 122 535 (58.0%) were male and 88 665 (42.0%) were female, and 188 895 (89.4%) were born in Sweden. The mean (SD) age at the first blood sampling (baseline) was 42.1 (12.6) years (Table 1). During a mean (SD) follow-up time of 21.0 (6.7) years, a total of 16 256 participants were diagnosed with depression, anxiety, or stress-related disorders (incidence rate, 36.4 per 10 000 person-years), with a mean (SD) age at diagnosis of 60.5 (15.6) years. Among these, 9725 (4.6%) were diagnosed with depression (incidence rate, 21.5 per 10 000 person-years), 7582 (3.6%) were diagnosed with anxiety (incidence rate, 16.6 per 10 000 person-years), and 4833 (2.3%) were diagnosed with stress-related disorders (incidence rate, 10.5 per 10 000 person-years). A total of 3128 participants (1.5%) were diagnosed with both depression and anxiety, whereas less than 1% were diagnosed with both depression and stress-related disorders (1978 [0.9%]) or with both anxiety and stress-related disorders (1544 [0.7%]). Only 984 participants (0.4%) had received all 3 diagnoses.

**Table 1.** Baseline Characteristics of the Study Participants^a^

Characteristic	All (N = 211 200)	Female (n = 88 665)	Male (n = 122 535)
Age at first blood sampling in years, mean (SD), y	42.1 (12.6)	42.4 (13.1)	41.8 (12.3)
Country of birth			
Sweden	188 895 (89.4)	78 446 (88.5)	110 449 (90.1)
Other Nordic countries	10 992 (5.2)	5687 (6.4)	5305 (4.3)
Other countries	11 308 (5.4)	4531 (5.1)	6777 (5.5)
Missing	5 (<0.1)	1 (<0.1)	4 (<0.1)
Socioeconomic status			
Low	96 256 (45.6)	47 745 (53.9)	48 511 (39.6)
High	89 865(42.6)	30 086 (33.9)	59 779 (48.8)
Missing	25 079 (11.8)	10 834 (12.2)	14 245 (11.6)
Glucose, mean (SD), mmol/L (n = 184 067)	4.9 (1.1)	4.8 (1.0)	5 (1.1)
Fasting status at glucose measurement			
Overnight fasting	98 562 (53.6)	40 045 (51.7)	58 517 (54.9)
No fasting	85 405 (46.4)	37 326 (48.2)	48 079 (45.1)
Missing	100 (<0.1)	52 (0.1)	48 (<0.1)
TC, mean (SD), mmol/L (n = 191 074)	5.5 (1.1)	5.4 (1.1)	5.5 (1.1)
Fasting status at TC measurement			
Overnight fasting	101 447 (53.1)	41 197 (51.5)	60 250 (54.2)
No fasting	89 522 (46.9)	38 717 (48.4)	50 805 (45.8)
Missing	105 (<0.1)	57 (0.1)	47 (<0.1)
TG, mean (SD), mmol/L (n = 190 089)	0.09 (0.8)	−0.1 (0.7)	0.3 (0.8)
Fasting status at TGs measurement			
Overnight fasting	101 452 (53.4)	41 230 (51.8)	60 222 (54.6)
No fasting	88 532 (46.6)	38 391 (48.2)	50 141 (45.4)
Missing	105 (<0.1)	57 (<0.1)	48 (<0.1)
HDL-C, mean (SD), mmol/L (n = 87 299)	1.6 (0.4)	1.7 (0.4)	1.4 (0.3)
Fasting status at HDL-C measurement			
Overnight fasting	51 056 (58.5)	19 850 (56.1)	31 206 (60.1)
No fasting	36 190 (41.5)	15 514 (43.8)	20 676 (39.8)
Missing	53 (<0.1)	28 (0.1)	25 (0.1)
LDL-C, mean (SD), mmol/L (n = 83 092)	3.5 (1.0)	3.4 (1.0)	3.6 (1.0)
Fasting status at LDL-C measurement			
Overnight fasting	45 674 (55.0)	17 612 (53.0)	28 062 (56.3)
No fasting	37 376 (45.0)	15 609 (47.0)	21 767 (43.7)
Missing	42 (<0.1)	19 (<0.1)	23 (<0.1)
LDL-C/HDL-C ratio, mean (SD) (n = 79 629)^b^	1.1 (0.6)	0.9 (0.6)	1.3 (0.6)
Fasting status at LDL-C/HDL-C ratio measurement			
Overnight fasting	43 465 (54.6)	16 861 (52.1)	26 604 (56.4)
No fasting	36 122 (45.4)	15 506 (47.9)	20 616 (43.6)
Missing	42 (<0.1)	19 (<0.1)	23 (0.1)
ApoA-I, mean (SD), mmol/L (n = 72 446)	1.4 (0.2)	1.5 (0.2)	1.3 (0.2)
Fasting status at ApoA-I measurement			
Overnight fasting	42 439 (58.6)	15 567 (55.6)	26 872 (60.5)
No fasting	29 974 (41.4)	12 423 (44.4)	17 551 (39.5)
Missing	33 (<0.1)	18 (<0.1)	15 (<0.1)
ApoB, mean (SD), mmol/L (n = 62 549)	1.2 (0.3)	1.1 (0.3)	1.2 (0.3)
Fasting status at ApoB measurement			
Overnight fasting	37 700 (60.3)	14 075 (56.9)	23 625 (62.5)
No fasting	24 794 (39.6)	10 644 (43.0)	14 150 (37.4)
Missing	55 (0.1)	33 (0.1)	22 (0.1)
ApoB/ApoA-I ratio, mean (SD) (n = 53 669)^b^	−0.3 (0.5)	−0.5 (0.5)	−0.2 (0.4)
Fasting status at ApoB/ApoA-I ratio measurement			
Overnight fasting	34 957 (65.1)	12 687 (62.2)	22 270 (66.9)
No fasting	18 681 (34.8)	7693 (37.7)	10 988 (33.0)
Missing	31 (0.1)	18 (0.1)	13 (0.1)

Abbreviations: ApoA-I, apolipoprotein A-I; ApoB, apolipoprotein B; HDL-C, high-density lipoprotein cholesterol; LDL-C, low-density lipoprotein cholesterol; TC, total cholesterol; TG, triglycerides.

SI conversion factors: To convert glucose to milligrams per deciliter, divide by 0.0555; to convert TC, HDL-C, and LDL-C to milligrams per deciliter, divide by 0.0259; to convert TGs to milligrams per deciliter, divide by 0.0113; to convert ApoA-I and ApoB to milligrams per deciliter, multiply by 0.01.

^a^ Data are presented as number (percentage) of participants unless otherwise indicated.

^b^ Logarithmically (log_2_) transformed.

Compared with low or normal levels, high levels of glucose (HR, 1.30; 95% CI, 1.20-1.41) and TGs (HR, 1.15; 95% CI, 1.10-1.20) were associated with a higher risk of depression, anxiety, and stress-related disorders, whereas a high level of HDL-C was associated with a lower risk (HR, 0.88; 95% CI, 0.80-0.97) (Table 2). For instance, a glucose level higher than normal (>6.1 mmol/L) was noted in 31 290 participants (5.9%), whereas a glucose level of lower than normal (<3.9 mmol/L) was noted in 22 872 participants (4.3%). The risk for depression, anxiety, or stress-related disorders did not differ between individuals with a low glucose level and individuals with a normal glucose level (adjusted HR, 0.94; 95% CI, 0.88-1.02).

**Table 2.** IRs per 10 000 Person-Years and aHRs of Depression, Anxiety, or Stress-Related Disorders

Biomarker^a^	Depression, anxiety, or stress-related disorders		Depression		Anxiety		Stress-related disorders	
	No. of events (IR)	aHR (95% CI)	No. of events (IR)	aHR (95% CI)	No. of events (IR)	aHR (95% CI)	No. of events (IR)	aHR (95% CI)
**High glucose (≥6.11 mmol/L)**								
Yes	622 (41.5)	1.30 (1.20-1.41)	416 (27.4)	1.36 (1.23-1.50)	261 (17.0)	1.22 (1.08-1.38)	130 (8.5)	1.25 (1.04-1.49)
No	13 455 (36.2)	1.0 [Reference]	7949 (21.1)	1.0 [Reference]	6338 (16.7)	1.0 [Reference]	4164 (10.9)	1.0 [Reference]
**High total cholesterol (≥5.00 mmol/L)**								
Yes	8987 (35.1)	1.01 (0.98-1.05)	5532 (21.3)	1.00 (0.95-1.05)	4154 (15.9)	1.04 (0.99-1.10)	2248 (8.6)	1.00 (0.94-1.06)
No	5575 (38.4)	1.0 [Reference]	3151 (21.4)	1.0 [Reference]	2662 (17.9)	1.0 [Reference]	2157 (14.5)	1.0 [Reference]
**High triglycerides (≥1.71 mmol/L)**								
Yes	2497 (36.2)	1.15 (1.10-1.20)	1608 (23.0)	1.18 (1.12-1.25)	1132 (16.1)	1.16 (1.09-1.24)	645 (9.15)	1.21 (1.11-1.32)
No	11 982 (36.3)	1.0 [Reference]	7030 (21.0)	1.0 [Reference]	5644 (16.7)	1.0 [Reference]	3735 (11.0)	1.0 [Reference]
**High LDL-C (≥3.00 mmol/L)**								
Yes	3973 (33.8)	1.01 (0.95-1.06)	2459 (20.7)	0.98 (0.91-1.06)	1810 (15.1)	0.99 (0.91-1.07)	945 (7.9)	0.98 (0.88-1.08)
No	2234 (37.0)	1.0 [Reference]	1279 (20.9)	Ref	1087 (17.7)	Ref	834 (13.5)	Ref
**High HDL-C (≥1.03 mmol/L)**								
Yes	6019 (34.8)	0.88 (0.80-0.97)	3583 (20.4)	0.91 (0.80-1.03)	2820 (16.0)	0.78 (0.68-0.90)	1762 (10.0)	0.87 (0.72-1.05)
No	448 (33.2)	1.0 [Reference]	271 (19.8)	1.0 [Reference]	228 (16.6)	1.0 [Reference]	122 (8.8)	1.0 [Reference]
**High LDL-C/HDL-C ratio (≥3.50)**								
**Yes**	701 (31.6)	1.06 (0.97-1.14)	444 (19.8)	1.04 (0.94-1.15)	325 (14.4)	1.10 (0.98-1.24)	139 (6.2)	0.97 (0.81-1.16)
No	5 252 (35.3)	1.0 [Reference]	3114 (20.7)	1.0 [Reference]	2465 (16.3)	1.0 [Reference]	1591 (10.4)	1.0 [Reference]
**High ApoA-I (≥1.00 mmol/L in men and 1.10 mmol/L in women)**								
Yes	5269 (34.3)	0.92 (0.78-1.10)	3155 (20.3)	0.83 (0.67-1.03)	2502 (16.0)	1.04 (0.80-1.35)	1499 (9.6)	0.88 (0.66-1.19)
No	135 (37.4)	1.0 [Reference]	88 (24.1)	1.0 [Reference]	58 (15.7)	1.0 [Reference]	45 (12.2)	1.0 [Reference]
**High ApoB (≥0.90 mmol/L)**								
Yes	3574 (32.9)	0.95 (0.88-1.02)	2159 (19.7)	0.94 (0.85-1.04)	1690 (15.3)	1.02 (0.91-1.13)	901 (8.1)	0.86 (0.76-0.98)
No	1001 (37.6)	1.0 [Reference]	548 (20.2)	1.0 [Reference]	463 (17.0)	1.0 [Reference]	405 (14.8)	1.0 [Reference]
**High ApoB/ApoA-I ratio (≥0.90 in men and 0.80 in women)**								
Yes	1599 (32.9)	1.06 (0.99-1.13)	984 (20.0)	1.04 (0.96-1.14)	765 (15.5)	1.12 (1.01-1.23)	355 (7.2)	1.00 (0.87-1.14)
No	2207 (33.2)	1.0 [Reference]	1274 (18.9)	1.0 [Reference]	1043 (15.4)	1.0 [Reference]	715 (10.5)	1.0 [Reference]

Abbreviations: aHR, adjusted hazard ratio; ApoA-I, apolipoprotein A-I; ApoB, apolipoprotein B; HDL-C, high-density lipoprotein cholesterol; IR, incidence rate; LDL-C, low-density lipoprotein cholesterol.

SI conversion factors: To convert glucose to milligrams per deciliter, divide by 0.0555; to convert total cholesterol, HDL-C, and LDL-C to milligrams per deciliter, divide by 0.0259; to convert triglycerides to milligrams per deciliter, divide by 0.0113; to convert ApoA-I and ApoB to milligrams per deciliter, multiply by 0.01.

^a^ Adjusted for age at first blood sampling, sex, fasting status at first blood sampling, country of birth, and socioeconomic status.

These results were consistent when examining depression, anxiety, and stress-related disorders separately. These associations were also comparable for men and women (eTable 3 in Supplement 1). Similar associations were noted when studying the associations per 1-SD increase of the biomarker levels (Table 3).

**Table 3.** IRs per 10 000 Person-Years and aHRs of Depression, Anxiety, or Stress-Related Disorders for 1-SD Increase in Carbohydrate, Lipid, and Apolipoprotein Biomarker Levels

Biomarker^a^	Depression, anxiety, or stress-related disorders		Depression		Anxiety		Stress-related disorders	
	No. of events (IR)	aHR (95% CI)	No. of events (IR)	aHR (95% CI)	No. of events (IR)	aHR (95% CI)	No. of events (IR)	aHR (95% CI)
Glucose	14 077 (36.4)	1.07 (1.05-1.09)	8365 (21.4)	1.08 (1.05-1.10)	6599 (16.8)	1.07 (1.04-1.10)	4294 (10.9)	1.07 (1.03-1.11)
TC	14 562 (36.5)	1.02 (1.00-1.04)	8683 (21.4)	1.02 (1.00-1.05)	6816 (16.7)	1.04 (1.01-1.07)	4405 (10.8)	1.00 (0.96-1.03)
TG	14 479 (36.3)	1.07 (1.05-1.09)	8638 (21.4)	1.08 (1.06-1.10)	6776 (16.7)	1.08 (1.06-1.10)	4380 (10.7)	1.07 (1.04-1.11)
LDL-C	6207 (34.9)	1.01 (0.98-1.04)	3738 (20.8)	1.00 (0.96-1.03)	2897 (16.0)	1.03 (0.99-1.08)	1779 (9.8)	0.99 (0.94-1.06)
HDL-C	6467 (34.7)	0.94 (0.92-0.97)	3854 (20.4)	0.95 (0.92-0.99)	3048 (16.1)	0.92 (0.89-0.96)	1884 (9.9)	0.95 (0.90-1.00)
LDL-C/HDL-C ratio	5953 (34.9)	1.03 (1.00-1.07)	3558 (20.6)	1.01 (0.98-1.06)	2790 (16.1)	1.06 (1.01-1.11)	1730 (9.9)	1.04 (0.98-1.10)
ApoA-I	5404 (34.4)	0.97 (0.94-1.00)	3243 (20.4)	0.98 (0.94-1.02)	2560 (16.0)	0.97 (0.93-1.02)	1544 (9.6)	0.97 (0.91-1.03)
ApoB	4575 (33.9)	0.98 (0.94-1.01)	2707 (19.8)	0.98 (0.94-1.03)	2153 (15.7)	1.00 (0.95-1.06)	1306 (9.5)	0.92 (0.85-0.99)
ApoB/ApoA-I ratio	3806 (33.1)	1.01 (0.97-1.05)	2258 (19.4)	1.00 (0.96-1.06)	1808 (15.5)	1.03 (0.97-1.09)	1070 (9.1)	0.98 (0.91-1.06)

Abbreviations: ApoA-I, apolipoprotein A-I; ApoB, apolipoprotein B; HDL-C, high-density lipoprotein cholesterol; IR, incidence rate; LDL-C, low-density lipoprotein cholesterol; TC, total cholesterol; TG, triglycerides.

^a^ Adjusted for age at first blood sampling, sex, fasting status, country of birth, and socioeconomic status.

The sensitivity analysis including only employed individuals demonstrated similar results to those of the main analysis (eTables 4 and 5 in Supplement 1). Finally, when examining the associations using biomarker measurements through referral by outpatient care, we observed similar results for glucose and TGs, whereas the results for HDL-C diminished greatly. In this analysis, we also found a high LDL-C, TC, ApoB, and ApoB/ApoA-I ratio to be associated with a lower risk of depression, anxiety, and stress-related disorders, collectively or individually (eTable 6 in Supplement 1). The percentage of individuals with depression, anxiety, or stress-related disorders tended to be lower among individuals with high socioeconomic status (5967 [6.7%]) compared with individuals with low (8166 [8.5%]) or missing (2251 [9.0%]) socioeconomic status (eTable 7 in Supplement 1). Similar results to the ones from the main analysis were also found after excluding participants with missing socioeconomic status (eTable 8 in Supplement 1).

The Figure and eTable 9 in Supplement 1 show the biomarker levels up to 30 years before diagnosis of the cases and their matched controls. Compared with controls, patients with depression, anxiety, or stress-related disorders had consistently higher levels of glucose, TGs, and TC during 20 years before diagnosis. Patients with depression, anxiety, or stress-related disorders also had higher levels of ApoA-I and ApoB during the 10 years before diagnosis compared with controls. Similar findings were observed for depression but not for anxiety or stress-related disorders (eFigure 2 in Supplement 1).

**Figure.**
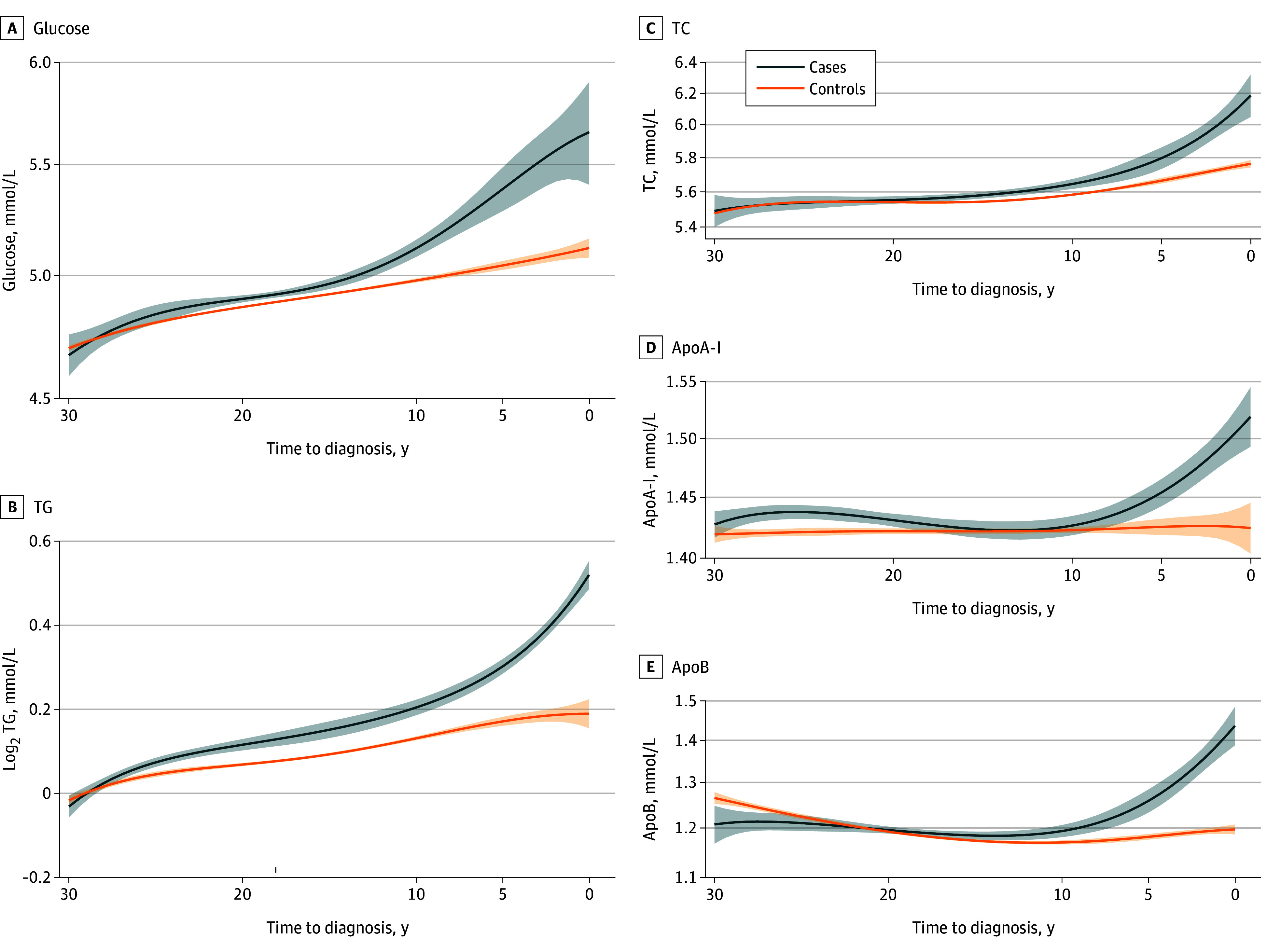
Mean Concentrations of Blood Biomarkers During the 30 Years Before Diagnosis of Depression, Anxiety, or Stress-Related Disorders The method of local polynomial smoothing with fourth-degree polynomial function and gaussian kernel function was used to plot the mean concentrations of the biomarkers over time before the index date with 95% CIs. ApoA-I indicates apolipoprotein A-I (to convert to milligrams per deciliter, multiply by 0.01); ApoB, apolipoprotein B (to convert to milligrams per deciliter, multiply by 0.01); TC, total cholesterol (to convert to milligrams per deciliter, multiply by 0.0259); TG, triglycerides (to convert to milligrams per deciliter, multiply by 0.0113). Shaded areas represent 95% CIs.

## Discussion

In this large, population-based, longitudinal cohort study, we found that elevated levels of glucose and TGs as well as lower levels of HDL-C were associated with increased risk of common psychiatric disorders, namely, depression, anxiety, or stress-related disorders. These results did not differ among the 3 disorders or between men and women. In addition, these biomarkers showed different levels between individuals who developed depression, anxiety, or stress-related disorders and those not developing such during many years before diagnosis.

### Comparison With Previous Studies

Previous studies have suggested that low levels of HDL-C,^13,14,15,20,50^ high levels of TGs,^13,14,15^ and low levels of LDL-C and TC^14,21,23^ are associated with a higher risk of depressive disorders. The current study provided similar evidence for TGs and HDL-C but not for TC and LDL-C. A novel finding is that high levels of TGs and low HDL-C were associated with increased risk of anxiety and stress-related disorders. Additionally, we found that individuals with depression, anxiety, or stress-related disorders demonstrated higher levels of TGs and TC already 20 years before diagnosis compared with individuals not developing these disorders.

No previous longitudinal study to our knowledge has investigated the association of apolipoproteins with anxiety and stress-related disorders. In our study, no association was found for any of the apolipoprotein biomarkers in the time to event analysis. In contrast, in the temporal trend analysis, we observed that individuals with the studied psychiatric disorders, especially depression, demonstrated elevated levels of ApoA-I and ApoB during the 10 years before diagnosis. It is possible that presymptomatic stages of these disorders explained the observed temporal pattern of these biomarkers.

Our finding of a positive association between glucose and risk of depression, anxiety, and stress-related disorders is partly supported by the existing literature. Some studies found that chronic hyperglycemia in the context of diabetes is associated with an increased risk of depression, anxiety, and stress-related disorders in some studies.^16,17,24,25,26,27,29,31,35,36,51,52^ Similarly, several other studies found that levels of glucose and glycated hemoglobin are associated with the development of depressive disorders.^13,15,16^ However, some studies failed to demonstrate an association between diabetes and incident depressive disorders^28,53^ or showed that the association was in fact mediated by the stress of managing diabetes.^18^ The latter hypothesis is not possible to test in our study because the biomarker measurement (1985-1996) was performed many years before information on treatment of diabetes (eg, use of antidiabetics) became available.

Low levels of HDL-C and elevated TG were also associated with increased risk of depression, anxiety, or stress-related disorders. Low HDL-C levels and elevated levels of TG are both linked to obesity-related inflammation,^54^ which, together with the subsequent disruption in leptin signaling, might contribute to a shared biological mechanism between obesity and mood dysregulation.^55,56^

Stress-related disorders usually follow a stressful event or psychological trauma.^57^ The elevated levels of glucose and TG observed in individuals with such diagnoses could be indicative of a maladaptive autonomic response, including aberrant sympathetic activity that often occurs in individuals previously exposed to a traumatic event.^58,59^ This finding indicates the need for early identification of patients at risk for persistent psychiatric response to trauma or significant life changes, because early intervention can prevent disease progression and cardiometabolic comorbidities.^60^

The absence of a modifying effect of sex on the association of metabolic biomarkers with depression, anxiety, and stress-related disorders is in line with a previous study.^23^ These findings together suggest a universal role of these biomarkers in the risk of common psychiatric disorders, irrespective of sex. The different results for LDL-C, ApoB, and TC in the sensitivity analysis conducted for biomarkers measured through referral by outpatient care might, however, suggest a potential contribution of confounding by indication. This finding indicates the importance of taking into consideration indication bias when using administrative databases for purposes of this kind. The main analysis was conducted only among individuals at least 16 years old at baseline, because we focused on biomarker measurements through health screening in relation to a job.

Because depression, anxiety, and stress-related disorders often co-occur^61,62,63^ and there is a possibility of overlapping symptoms among these disorders, one of the outcomes we studied was any diagnosis of depression, anxiety, or stress-related disorders. In the current study, depression was diagnosed in 4.6%, anxiety in 3.6%, and stress-related disorders in 2.3% of the participants; 1.5% of the participants were diagnosed with both depression and anxiety, whereas less than 1% were diagnosed with both depression and stress-related disorders (0.9%) or with both anxiety and stress-related disorders (0.7%). Only 0.4% of the participants had received all 3 diagnoses.

To our knowledge, this study is the first to explore the association of a wide range of metabolic biomarkers measured for screening purposes with the risk of depression, anxiety, or stress-related disorders using a cohort with a follow-up time of up to 30 years. The ascertainment of biomarkers, psychiatric disorders, and covariates was achieved using prospectively and independently collected data, therefore alleviating the concern of differential misclassification and adding to the internal validity of our study. We disregarded the first 5 years of follow-up because the literature has shown an average diagnostic delay of 5 years for these disorders.^64,65,66,67^ The rationale behind this decision was to avoid estimates affected by potential confounding, namely, that the levels of the metabolic biomarkers might be the result of, instead of the risk factor for, the studied disorders.

There are several factors that might limit the comparability of our work with previous studies. Several of the previous studies have had relatively short follow-up times,^15,17,19,21,22,24^ leading to potentially limited statistical power. Most of the existing studies focused on depression and used primarily questionnaires or scales for depressive symptoms instead of a clinical diagnosis of depression.^15,17,18,19,20,23,24^ Furthermore, a previous longitudinal study almost exclusively explored depression.^68^ Our findings on anxiety and stress-related disorders therefore add to the existing knowledge base.

### Limitations

This study has some weaknesses. First, because our study participants were employed at the time of recruitment (ie, underwent a health screening in relation to a job), the generalizability of our findings to the general population is limited. The participants in the AMORIS cohort were comparable in terms of age, sex, and country of birth with the general population of Stockholm County in 1990 and in terms of socioeconomic status with the employed individuals living in Stockholm that year.^41^ The higher employment rate in the AMORIS cohort can be attributed to their recruitment for laboratory testing through occupational health screening, which, in turn, accounts for the lower all-cause mortality observed compared with the general population.^41^ Second, although we adjusted for several covariates, residual confounding cannot be excluded due to unmeasured confounders, such as body mass index, smoking, physical activity, diet, and early-life factors (eg, adverse childhood experiences),^69,70^ because the AMORIS cohort has limited information on other potential risk factors for the studied psychiatric disorders. Given the fact that all participants were recruited though participating in an occupational health checkup, the study population was in general comparatively healthy. It is possible that in future studies, accounting for body mass index would explain away part of the association and thus produce slightly different estimates.^71^ Third, our findings are likely to be subject to detection bias because individuals with abnormal levels of the studied biomarkers might be more closely monitored by health care practitioners. Previous literature on primary care in Sweden indicated that depression, anxiety, and stress-related disorders were underdiagnosed.^72^ Fourth, because the Swedish Patient Register only includes information from hospital-based inpatient and specialized outpatient care, there is inevitably some degree of misclassification in the ascertainment of psychiatric disorders in this study. For instance, patients with psychiatric disorders attended by primary care alone as well as individuals with psychiatric symptoms never attended by the health care system would have been classified as free of psychiatric disorders. Related to this, because the studied disorders commonly fluctuate in symptom severity, some of the newly diagnosed cases identified during follow-up might indeed represent symptom exacerbations of previously undiagnosed disorders.

## Conclusions

In this large, population-based, longitudinal cohort study, we found elevated levels of glucose and TGs and reduced levels of HDL-C to be associated with a higher risk of subsequent diagnosis of depression, anxiety, and stress-related disorders. This study provides further longitudinal evidence that metabolic dysregulation or the metabolic syndrome increases the risk of developing common psychiatric disorders. These results add further evidence of the association between cardiometabolic health and psychiatric disorders and potentially advocate for a closer follow-up of individuals with metabolic dysregulations for prevention and early diagnosis of psychiatric disorders. Additional studies are needed to explore whether rigorous or earlier interventions for cardiometabolic diseases could counteract such an association.

## Notes

Supplement 1. **eTable 1.** ICD Codes Used for Outcome Ascertainment **eTable 2.** Descriptive Statistics of Matching Variables Between Cases and Controls **eTable 3.** Incidence Rates (IR) per 10 000 Person-Years and Adjusted Hazard Ratios (aHRs) With 95% Confidence Intervals (CI) of Depression, Anxiety, or Stress-Related Disorders in Relation to High Versus Low Levels Of Carbohydrate, Lipid, and Apolipoprotein Biomarkers, Analysis Stratified by Sex **eTable 4.** Incidence Rates (IR) per 10 000 Person-Years and Adjusted Hazard Ratios (aHRs) With 95% Confidence Intervals (CI) of Depression, Anxiety, or Stress-Related Disorders in Relation to High Versus Low Levels of Carbohydrate, Lipid, and Apolipoprotein Biomarkers Among the 161 237 Definitely Employed Individuals–A Study Based on AMORIS Cohort **eTable 5.** Incidence Rates (IR) per 10 000 Person-Years and Adjusted Hazard Ratios (aHRs) With 95% Confidence Intervals (CI) of Depression, Anxiety, or Stress-Related Disorders for One Standard Deviation Increase in the Levels of Carbohydrate, Lipid, and Apolipoprotein Biomarkers Among the 161 237 Definitely Employed Individuals – A Study Based on AMORIS Cohort **eTable 6.** Incidence Rates (IR) per 10 000 Person-Years and Adjusted Hazard Ratios (aHRs) With 95% Confidence Intervals (CI) of Depression, Anxiety, or Stress-Related Disorders in Relation to High Versus Low Levels of Carbohydrate, Lipid, and Apolipoprotein Biomarkers Among Individuals With Biomarker Measured Through Referral by Outpatient Care–A Study Based on AMORIS Cohort **eTable 7.** Number (%) of Participants With Diagnosis of Depression, Anxiety and Stress-Related Disorders Among Individuals With Low, High or Missing Socioeconomic Status **eTable 8.** Incidence Rates (IR) per 10 000 Person-Years and Adjusted Hazard Ratios (aHRs) With 95% Confidence Intervals (CI) of Depression, Anxiety, or Stress-Related Disorders in Relation to High Versus Low Levels of Carbohydrate, Lipid, and Apolipoprotein Biomarkers, Excluding From the Analysis Individuals Missing Socioeconomic Status–A Study Based on AMORIS Cohort **eTable 9.** Observed and Predicted Biomarker Levels Among Cases and Controls During up to 30 Years Before Diagnosis of the Cases and Their Matched Controls **eFigure 1.** Flowchart of the Study Design **eFigure 2.** Mean Concentrations of Blood Biomarkers of Lipid, Carbohydrate, and Apolipoprotein Metabolism During the 30 Years Before the Diagnosis of Depression, Anxiety, of Stress-Related Disorders, Comparing Patients With Such Disorders (Green Area) to the Matched Controls (Pink Area)

Supplement 2.
Data Sharing Statement
