# Supplementary material for: Metabolic Profile and Long-Term Risk of Depression, Anxiety, and Stress-Related Disorders
Source: JAMA Netw Open. 2024 Apr 2;7(4):e244525. doi: 10.1001/jamanetworkopen.2024.4525 (PMC10988352; doi:10.1001/jamanetworkopen.2024.4525)
Supplement: Supplement 1. — eTable 1. ICD Codes Used for Outcome Ascertainment eTable 2. Descriptive Statistics of Matching Variables Between Cases and Controls eTable 3. Incidence Rates (IR) per 10 000 Person-Years and Adjusted Hazard Ratios (aHRs) With 95% Confidence Intervals (CI) of Depression, Anxiety, or Stress-Related Disorders in Relation to High Versus Low Levels Of Carbohydrate, Lipid, and Apolipoprotein Biomarkers, Analysis Stratified by Sex eTable 4. Incidence Rates (IR) per 10 000 Person-Years and Adjusted Hazard Ratios (aHRs) With 95% Confidence Intervals (CI) of Depression, Anxiety, or Stress-Related Disorders in Relation to High Versus Low Levels of Carbohydrate, Lipid, and Apolipoprotein Biomarkers Among the 161 237 Definitely Employed Individuals–A Study Based on AMORIS Cohort eTable 5. Incidence Rates (IR) per 10 000 Person-Years and Adjusted Hazard Ratios (aHRs) With 95% Confidence Intervals (CI) of Depression, Anxiety, or Stress-Related Disorders for One Standard Deviation Increase in the Levels of Carbohydrate, Lipid, and Apolipoprotein Biomarkers Among the 161 237 Definitely Employed Individuals – A Study Based on AMORIS Cohort eTable 6. Incidence Rates (IR) per 10 000 Person-Years and Adjusted Hazard Ratios (aHRs) With 95% Confidence Intervals (CI) of Depression, Anxiety, or Stress-Related Disorders in Relation to High Versus Low Levels of Carbohydrate, Lipid, and Apolipoprotein Biomarkers Among Individuals With Biomarker Measured Through Referral by Outpatient Care–A Study Based on AMORIS Cohort eTable 7. Number (%) of Participants With Diagnosis of Depression, Anxiety and Stress-Related Disorders Among Individuals With Low, High or Missing Socioeconomic Status eTable 8. Incidence Rates (IR) per 10 000 Person-Years and Adjusted Hazard Ratios (aHRs) With 95% Confidence Intervals (CI) of Depression, Anxiety, or Stress-Related Disorders in Relation to High Versus Low Levels of Carbohydrate, Lipid, and Apolipoprotein Biomarkers, Excluding From the Analysis Individuals Miss [file jamanetwopen-e244525-s001.pdf]

Supplementary Online Content

Chourpiliadis C, Zeng Y, Lovik A, et al. Metabolic profile and long-term risk of depression, anxiety, and stress-related disorders. *JAMA Netw Open*. 2024;7(4):e244525. doi:10.1001/jamanetworkopen.2024.4525

eTable 1. ICD Codes Used for Outcome Ascertainment
eTable 2. Descriptive Statistics of Matching Variables Between Cases and Controls
eTable 3. Incidence Rates (IR) per 10 000 Person-Years and Adjusted Hazard Ratios (aHRs) With 95% Confidence Intervals (CI) of Depression, Anxiety, or Stress-Related Disorders in Relation to High Versus Low Levels Of Carbohydrate, Lipid, and Apolipoprotein Biomarkers, Analysis Stratified by Sex
eTable 4. Incidence Rates (IR) per 10 000 Person-Years and Adjusted Hazard Ratios (aHRs) With 95% Confidence Intervals (CI) of Depression, Anxiety, or Stress-Related Disorders in Relation to High Versus Low Levels of Carbohydrate, Lipid, and Apolipoprotein Biomarkers Among the 161 237 Definitely Employed Individuals– A Study Based on AMORIS Cohort
eTable 5. Incidence Rates (IR) per 10 000 Person-Years and Adjusted Hazard Ratios (aHRs) With 95% Confidence Intervals (CI) of Depression, Anxiety, or Stress-Related Disorders for One Standard Deviation Increase in the Levels of Carbohydrate, Lipid, and Apolipoprotein Biomarkers Among the 161 237 Definitely Employed Individuals – A Study Based on AMORIS Cohort
eTable 6. Incidence Rates (IR) per 10 000 Person-Years and Adjusted Hazard Ratios (aHRs) With 95% Confidence Intervals (CI) of Depression, Anxiety, or Stress-Related Disorders in Relation to High Versus Low Levels of Carbohydrate, Lipid, and Apolipoprotein Biomarkers Among Individuals With Biomarker Measured Through Referral by Outpatient Care – A Study Based on AMORIS Cohort
eTable 7. Number (%) of Participants With Diagnosis of Depression, Anxiety and Stress-Related Disorders Among Individuals With Low, High or Missing Socioeconomic Status
eTable 8. Incidence Rates (IR) per 10 000 Person-Years and Adjusted Hazard Ratios (aHRs) With 95% Confidence Intervals (CI) of Depression, Anxiety, or Stress-Related Disorders in Relation to High Versus Low Levels of Carbohydrate, Lipid, and Apolipoprotein Biomarkers, Excluding From the Analysis Individuals Missing Socioeconomic Status – A Study Based on AMORIS Cohort
eTable 9. Observed and Predicted Biomarker Levels Among Cases and Controls During up to 30 Years Before Diagnosis of the Cases and Their Matched Controls
eFigure 1. Flowchart of the Study Design
eFigure 2. Mean Concentrations of Blood Biomarkers of Lipid, Carbohydrate, and Apolipoprotein Metabolism During the 30 Years Before the Diagnosis of Depression, Anxiety, or Stress-Related Disorders, Comparing Patients With Such Disorders (Green Area) to the Matched Controls (Pink Area)

This supplementary material has been provided by the authors to give readers additional information about their work.

© 2024 Chourpiliadis C et al. *JAMA Network Open.*

eTable 1. ICD codes used for outcome ascertainment

Diagnoses	ICD-8 (1968-1986)	ICD-9 (1987-1996)	ICD-10 (1997-)
Depression	296,0, 296,2, 298,0, 300,4	296B, 296D, 298A, 300E, 311X	F32-F33
Anxiety	300,0, 300,2	300A, 300C	F40-F41
Stress-related disorders	307	308X, 309A, 309B, 309X	F43

© 2024 Chourpiliadis C et al. *JAMA Network Open.*

eTable 2. Descriptive statistics of matching variables between cases and controls

Matching variables		Depression, anxiety, or stress-related disorders		Depression		Anxiety		Stress-related disorders	
		Cases	Controls	Cases	Controls	Cases	Controls	Cases	Controls
N		16 256	162 233	9 725	97 229	7 582	75 799	4 833	48 307
Female, N (%)		8 693 (53.5)	86 706 (53.5)	4 972 (51.1)	49 708 (51.1)	4 248 (56.0)	42 459 (56.0)	2 776 (57.4)	27 737 (57.4)
Age at first blood sampling in years, mean±SD		40.3± 12.9	40.3± 12.8	41.5± 13.0	41.3± 12.9	39.5± 12.8	39.6± 12.6	34.1± 10.5	34.3± 10.4
Calendar year of first blood sampling, N (%)	>1990	6 639 (42.0)	65 940 (41.6)	3 802 (39.1)	37 853 (38.9)	3 183 (42.0)	31 719 (41.8)	2 205 (45.6)	22 260 (46.1)
	≤1990	9 617 (58.0)	96 293 (58.4)	5 923 (60.9)	59 376 (61.1)	4 399 (58.0)	44 080 (58.2)	2 628 (54.3)	26 047 (53.9)

© 2024 Chourpiliadis C et al. *JAMA Network Open.*

eTable 3. Incidence rates (IR) per 10 000 person-years and adjusted hazard ratios (aHR) with 95% confidence intervals (CI) of depression, anxiety, or stress-related disorders in relation to high versus low levels of carbohydrate, lipid, and apolipoprotein biomarkers, analysis stratified by sex

**Biomarkers[a,b]**	**Depression, anxiety, or stress-related disorders**		**Depression**		**Anxiety**		**Stress-related disorders**	
	Males aHR (95% CI)	Females aHR (95% CI)	Males aHR (95% CI)	Females aHR (95% CI)	Males aHR (95% CI)	Females aHR (95% CI)	Males aHR (95% CI)	Females aHR (95% CI)
**High glucose (≥6.11 mmol/L)**								
**Yes**	**1.31 (1.18-1.46)**	**1.26 (1.10-1.43)**	**1.40 (1.23-1.58)**	**1.27 (1.08-1.50)**	1.15 (0.97-1.36)	**1.32 (1.10-1.58)**	1.22 (0.97-1.53)	1.23 (0.93-1.62)
**No**	Ref.	Ref.	Ref.	Ref.	Ref.	Ref.	Ref.	Ref.
**High total cholesterol (≥5.00 mmol/L)**								
**Yes**	1.03 (0.97-1.08)	1.00 (0.95-1.05)	1.02 (0.95-1.10)	0.99 (0.92-1.06)	1.03 (0.95-1.12)	1.05 (0.97-1.13)	1.02 (0.92-1.13)	0.98 (0.90-1.06)
**No**	Ref.	Ref.	Ref.	Ref.	Ref.	Ref.	Ref.	Ref.
**High triglycerides (≥1.71 mmol/L)**								
**Yes**	**1.12 (1.06-1.19)**	**1.21 (1.12-1.31)**	**1.18 (1.10-1.26)**	**1.20 (1.09-1.33)**	**1.13 (1.03-1.23)**	**1.24 (1.11-1.38)**	**1.18 (1.05-1.31)**	**1.28 (1.11-1.49)**
**No**	Ref.	Ref.	Ref.	Ref.	Ref.	Ref.	Ref.	Ref.
**High LDL-C (≥3.00 mmol/L)**								
**Yes**	0.99 (0.91-1.08)	1.00 (0.93-1.08)	0.96 (0.86-1.07)	0.97 (0.87-1.07)	**0.86 (0.76-0.98)**	1.05 (0.94-1.17)	0.95 (0.81-1.12)	0.98 (0.86-1.12)
**No**	Ref.	Ref.	Ref.	Ref.	Ref.	Ref.	Ref.	Ref.
**High HDL-C (≥1.03 mmol/L)**								
**Yes**	**0.88 (0.79-0.99)**	0.85 (0.69-1.05)	0.89 (0.77-1.02)	0.92 (0.69-1.23)	**0.79 (0.67-0.93)**	**0.75 (0.57-0.98)**	0.90 (0.72-1.13)	0.78 (0.54-1.12)
**No**	Ref.	Ref.	Ref.	Ref.	Ref.	Ref.	Ref.	Ref.
**High LDL-C/HDL-C (≥3.50)**								
**Yes**	0.99 (0.90-1.10)	**1.18 (1.02-1.37)**	0.98 (0.86-1.12)	1.15 (0.95-1.39)	1.06 (0.91-1.23)	1.15 (0.93-1.42)	0.80 (0.64-1.02)	1.30 (0.96-1.75)
**No**	Ref.	Ref.	Ref.	Ref.	Ref.	Ref.	Ref.	Ref.
**High ApoA-I (≥1.00 mmol/L in male and 1.10 mmol/L in female)**								
**Yes**	1.09 (0.84-1.43)	0.80 (0.63-1.01)	0.90 (0.66-1.23)	0.78 (0.57-1.06)	1.30 (0.84-2.00)	0.90 (0.64-1.27)	1.25 (0.73-2.12)	0.70 (0.49-1.01)
**No**	Ref.	Ref.	Ref.	Ref.	Ref.	Ref.	Ref.	Ref.
**High ApoB (≥0.90 mmol/L)**								
**Yes**	0.90 (0.80-1.02)	1.00 (0.91-1.10)	0.93 (0.79-1.09)	0.96 (0.84-1.10)	0.94 (0.78-1.13)	1.09 (0.95-1.25)	0.91 (0.73-1.13)	0.87 (0.74-1.02)
**No**	Ref.	Ref.	Ref.	Ref.	Ref.	Ref.	Ref.	Ref.
**High ApoB/ApoA-I (≥0.90 in male and 0.80 in female)**								
**Yes**	1.00 (0.90-1.10)	1.10 (1.00-1.22)	0.98 (0.86-1.10)	1.10 (0.96-1.26)	1.06 (0.92-1.23)	1.13 (0.98-1.29)	0.97 (0.80-1.17)	1.03 (0.85-1.25)
**No**	Ref.	Ref.	Ref.	Ref.	Ref.	Ref.	Ref.	Ref.

Abbreviations: ApoA-I: apolipoprotein A-I; ApoB: apolipoprotein B; HDL-C: high-density lipoprotein cholesterol; LDL-C: low-density lipoprotein cholesterol; Ref.: Reference

[a]Adjusted for age at first blood sampling, sex, fasting status, country of birth, and socioeconomic status

[b]Statistically significant estimates are highlighted with bold

© 2024 Chourpiliadis C et al. *JAMA Network Open.*

eTable 4. Incidence rates (IR) per 10 000 person-years and adjusted hazard ratios (aHR) with 95% confidence intervals (CI) of depression, anxiety, or stress-related disorders in relation to high versus low levels of carbohydrate, lipid, and apolipoprotein biomarkers among the 161 237 definitely employed individuals– a study based on AMORIS Cohort

**Biomarkers[a,b]**	**Depression, anxiety, or stress-related disorders**		**Depression**		**Anxiety**		**Stress-related disorders**	
	**No. of events (IR)**	**aHR (95% CI)**	**No. of events (IR)**	**aHR (95% CI)**	**No. of events (IR)**	**aHR (95% CI)**	**No. of events (IR)**	**aHR (95% CI)**
**High glucose (≥6.11 mmol/L)**								
**Yes**	464 (37.5)	**1.24 (1.13-1.36)**	310 (24.8)	**1.3 (1.16-1.46)**	189 (15.0)	1.11 (0.96-1.29)	102 (8.0)	1.19 (0.98-1.46)
**No**	11 357 (34.7)	Ref.	6 701 (20.2)	Ref.	5 349 (16.1)	Ref.	3 522 (10.5)	Ref.
**High total cholesterol (≥5.00 mmol/L)**								
**Yes**	7 217 (33.5)	1.01 (0.97-1.06)	4 423 (20.3)	1.01 (0.96-1.07)	3 340 (15.2)	1.03 (0.97-1.10)	1 853 (8.4)	0.99 (0.92-1.06)
**No**	4 604 (37.2)	Ref.	2 588 (20.6)	Ref.	2 198 (17.4)	Ref.	1 771 (14.0)	Ref.
**High triglycerides (≥1.71 mmol/L)**								
**Yes**	1 982 (34.6)	**1.16 (1.10-1.22)**	1 267 (21.9)	**1.19 (1.12-1.27)**	893 (15.3)	**1.15 (1.07-1.24)**	522 (8.9)	**1.2 (1.09-1.32)**
**No**	9 839 (34.9)	Ref.	5 744 (20.1)	Ref.	4 645 (16.2)	Ref.	3 102 (10.8)	Ref.
**High LDL-C (≥3.00 mmol/L)**								
**Yes**	2 425 (31.7)	0.97 (0.91-1.04)	1 461 (18.9)	0.95 (0.87-1.04)	1 126 (14.5)	0.96 (0.87-1.06)	620 (7.9)	0.94 (0.83-1.06)
**No**	1 585 (37.2)	Ref.	887 (20.5)	Ref.	781 (18.0)	Ref.	604 (13.9)	Ref.
**High HDL-C (≥1.03 mmol/L)**								
**Yes**	3 673 (33.4)	**0.79 (0.70-0.90)**	2 130 (19.1)	**0.8 (0.69-0.94)**	1 749 (15.6)	**0.69 (0.58-0.82)**	1 132 (10.1)	**0.78 (0.62-0.98)**
**No**	283 (35.9)	Ref.	171 (21.4)	Ref.	149 (18.6)	Ref.	82 (10.2)	Ref.
**High LDL-C/HDL-C (≥3.50)**								
**Yes**	425 (29.7)	1.04 (0.94-1.16)	265 (18.4)	1.03 (0.90-1.18)	206 (14.2)	1.12 (0.96-1.30)	91 (6.2)	0.94 (0.76-1.18)
**No**	3 450 (34.2)	Ref.	1 992 (19.5)	Ref.	1 648 (16.1)	Ref.	1 099 (10.7)	Ref.
**High ApoA-I (≥1.00 mmol/L in male and 1.10 mmol/L in female)**								
**Yes**	3 189 (33.6)	0.89 (0.72-1.09)	1 868 (19.5)	0.94 (0.71-1.25)	1 541 (16.0)	1.09 (0.79-1.52)	971 (10.0)	0.81 (0.57-1.15)
**No**	90 (38.9)	Ref.	49 (20.9)	Ref.	36 (15.2)	Ref.	33 (14.0)	Ref.
**High ApoB (≥0.90 mmol/L)**								
**Yes**	1 983 (32.1)	0.97 (0.88-1.07)	1 165 (18.7)	0.96 (0.84-1.09)	957 (15.3)	1.04 (0.90-1.20)	559 (8.9)	0.91 (0.78-1.07)
**No**	578 (36.8)	Ref.	313 (19.6)	Ref.	270 (16.9)	Ref.	238 (14.8)	Ref.
**High ApoB/ApoA-I (≥0.90 in male and 0.80 in female)**								
**Yes**	843 (31.9)	1.08 (0.98-1.18)	511 (19.1)	1.09 (0.97-1.23)	412 (15.4)	1.15 (1.00-1.31)	217 (8.05)	1.07 (0.91-1.27)
**No**	1 215 (32.3)	Ref.	674 (17.7)	Ref.	584 (15.3)	Ref.	411 (10.7)	Ref.

Abbreviations: ApoA-I: apolipoprotein A-I; ApoB: apolipoprotein B; HDL-C: high-density lipoprotein cholesterol; LDL-C: low-density lipoprotein cholesterol; Ref.: Reference

[a]Adjusted for age at first blood sampling, sex, fasting status at first blood sampling, country of birth, and socioeconomic status

[b]Statistically significant estimates are highlighted with bold

© 2024 Chourpiliadis C et al. *JAMA Network Open.*

eTable 5. Incidence rates (IR) per 10 000 person-years and adjusted hazard ratios (aHR) with 95% confidence intervals (CI) of depression, anxiety, or stress-related disorders for one standard deviation increase in the levels of carbohydrate, lipid, and apolipoprotein biomarkers among the 161 237 definitely employed individuals – a study based on AMORIS Cohort

**Biomarkers[a,b]**	**Depression, anxiety, or stress-related disorders**		**Depression**		**Anxiety**		**Stress-related disorders**	
	**No. of events (IR)**	**aHR (95% CI)**	**No. of events (IR)**	**aHR (95% CI)**	**No. of events (IR)**	**aHR (95% CI)**	**No. of events (IR)**	**aHR (95% CI)**
**Glucose**	11 821 (34.8)	**1.05 (1.03-1.07)**	7 011 (20.4)	**1.06 (1.04-1.09)**	5 538 (16.0)	**1.05 (1.02-1.08)**	3 624 (10.5)	**1.05 (1.02-1.09)**
**Total cholesterol**	11 821 (34.8)	1.02 (1.00-1.04)	7 011 (20.4)	1.02 (1.00-1.05)	5 538 (16.0)	1.03 (1.00-1.06)	3 624 (10.5)	0.99 (0.95-1.03)
**Triglycerides**	11 821 (34.8)	**1.07 (1.05-1.08)**	7 011 (20.4)	**1.07 (1.05-1.09)**	5 538 (16.0)	**1.08 (1.05-1.10)**	3 624 (10.5)	**1.07 (1.04-1.11)**
**LDL-C**	4 010 (33.6)	0.99 (0.96-1.03)	2 348 (19.5)	0.98 (0.93-1.02)	1 907 (15.7)	1.03 (0.98-1.08)	1 224 (10.1)	0.97 (0.91-1.05)
**HDL-C**	3 956 (33.6)	**0.94 (0.91-0.97)**	2 301 (19.3)	**0.94 (0.90-0.99)**	1 898 (15.8)	**0.93 (0.89-0.98)**	1 214 (10.1)	**0.93 (0.87-0.99)**
**LDL-C/HDL-C**	3 875 (33.7)	1.03 (0.99-1.07)	2 257 (19.4)	1.01 (0.96-1.06)	1 854 (15.8)	1.06 (1.00-1.12)	1 190 (10.1)	1.03 (0.96-1.10)
**ApoA-I**	3 279 (33.8)	0.97 (0.93-1.00)	1 917 (19.5)	0.98 (0.93-1.02)	1 577 (16.0)	0.97 (0.92-1.03)	1 004 (10.1)	0.97 (0.91-1.04)
**ApoB**	2 561 (33.1)	1.01 (0.96-1.06)	1 478 (18.9)	1.01 (0.95-1.07)	1 227 (15.6)	1.04 (0.97-1.11)	797 (10.1)	0.97 (0.89-1.07)
**ApoB/ApoA-I**	2 058 (32.1)	1.04 (0.99-1.10)	1 185 (18.3)	1.04 (0.98-1.11)	996 (15.3)	1.04 (0.97-1.12)	628 (9.6)	1.04 (0.94-1.15)

Abbreviations: ApoA-I: apolipoprotein A-I; ApoB: apolipoprotein B; HDL-C: high-density lipoprotein cholesterol; LDL-C: low-density lipoprotein cholesterol; Ref.: Reference

[a]Adjusted for age at first blood sampling, sex, fasting status, country of birth, and socioeconomic status;

[b]Statistically significant estimates are highlighted with bold

© 2024 Chourpiliadis C et al. *JAMA Network Open.*

eTable 6. Incidence rates (IR) per 10 000 person-years and adjusted hazard ratios (aHR) with 95% confidence intervals (CI) of depression, anxiety, or stress-related disorders in relation to high versus low levels of carbohydrate, lipid, and apolipoprotein biomarkers among individuals with biomarker measured through referral by outpatient care – a study based on AMORIS Cohort

**Biomarkers[a,b]**	**Depression, anxiety, or stress-related disorders** No. of events (IR)	**Depression, anxiety, or stress-related disorders** aHR (95% CI)	**Depression** No. of events (IR)	**Depression** aHR (95% CI)	**Anxiety** No. of events (IR)	**Anxiety** aHR (95% CI)	**Stress-related disorders** No. of events (IR)	**Stress-related disorders** aHR (95% CI)
**High glucose (≥6.11 mmol/L)**								
**Yes**	1 389 (61.8)	**1.21 (1.14-1.28)**	964 (42.3)	**1.27 (1.19-1.36)**	578 (25.0)	1.09 (1.00-1.19)	242 (10.4)	1.04 (0.91-1.18)
**No**	24 147 (60.0)	Ref.	14 774 (35.9)	Ref.	12 239 (29.5)	Ref.	7 628 (18.2)	Ref.
**High total cholesterol (≥5.00 mmol/L)**								
**Yes**	16 538 (55.7)	**0.96 (0.93-0.99)**	10 537 (34.9)	**0.96 (0.93-0.99)**	7 968 (26.2)	0.98 (0.94-1.02)	3 979 (12.9)	0.96 (0.91-1.00)
**No**	10 101 (67.4)	Ref.	5 916 (38.4)	Ref.	5 330 (34.4)	Ref.	4 108 (26.3)	Ref.
**High triglycerides (≥1.71 mmol/L)**								
**Yes**	5 220 (57.8)	**1.14 (1.10-1.17)**	3 389 (36.8)	**1.14 (1.10-1.19)**	2 471 (26.7)	**1.14 (1.09-1.19)**	1 247 (13.3)	**1.14 (1.07-1.21)**
**No**	21 330 (60.1)	Ref.	13 008 (35.9)	Ref.	10 786 (29.5)	Ref.	6 822 (18.5)	Ref.
**High LDL-C (≥3.00 mmol/L)**								
**Yes**	7 790 (50.3)	**0.85 (0.81-0.89)**	5 000 (31.8)	**0.83 (0.79-0.88)**	3 765 (23.7)	**0.88 (0.83-0.94)**	1 538 (9.6)	**0.84 (0.78-0.92)**
**No**	3 027 (64.8)	Ref.	1 858 (38.8)	Ref.	1 577 (32.7)	Ref.	1 027 (21.1)	Ref.
**High HDL-C (≥1.03 mmol/L)**								
**Yes**	9 614 (54.4)	0.99 (0.92-1.06)	6 093 (33.9)	1.02 (0.93-1.11)	4 754 (26.2)	0.94 (0.84-1.04)	2 298 (12.5)	0.92 (0.79-1.06)
**No**	858 (46.2)	Ref.	547 (29.0)	Ref.	419 (22.1)	Ref.	203 (10.6)	Ref.
**High LDL-C/HDL-C (≥3.50)**								
**Yes**	1 847 (45.5)	0.96 (0.91-1.01)	1 217 (29.6)	0.96 (0.90-1.02)	857 (20.7)	0.97 (0.90-1.04)	322 (7.7)	0.91 (0.80-1.02)
**No**	8 638 (55.7)	Ref.	5 429 (34.4)	Ref.	4 326 (27.2)	Ref.	2 177 (13.5)	Ref.
**High ApoA-I (≥1.00 mmol/L in male and 1.10 mmol/L in female)**								
**Yes**	9 656 (54.0)	**0.88 (0.78-0.99)**	6 138 (33.7)	**0.88 (0.76-1.02)**	4 766 (26.0)	0.86 (0.73-1.01)	2 266 (12.2)	0.90 (0.73-1.12)
**No**	292 (61.5)	Ref.	183 (37.7)	Ref.	153 (31.3)	Ref.	83 (16.8)	Ref.
**High ApoB (≥0.90 mmol/L)**								
**Yes**	8 303 (51.3)	**0.81 (0.77-0.86)**	5 335 (32.4)	**0.82 (0.77-0.88)**	4 044 (24.4)	**0.82 (0.76-0.89)**	1 744 (10.4)	0.79 (0.72-0.87)
**No**	1 721 (73.5)	Ref.	1 021 (42.3)	Ref.	929 (38.3)	Ref.	637 (25.9)	Ref.
**High ApoB/ApoA-I (≥0.90 in male and 0.80 in female)**								
**Yes**	5 226 (49.5)	**0.91 (0.88-0.95)**	3 394 (31.7)	**0.92 (0.88-0.97)**	2 461 (22.8)	**0.88 (0.83-0.93)**	995 (9.2)	**0.87 (0.80-0.95)**
**No**	4 746 (59.8)	Ref.	2 930 (36.2)	Ref.	2 484 (30.4)	Ref.	1 354 (16.4)	Ref.

Abbreviations: ApoA-I: apolipoprotein A-I; ApoB: apolipoprotein B; HDL-C: high-density lipoprotein cholesterol; LDL-C: low-density lipoprotein cholesterol; Ref.: Reference

[a]Adjusted for age at first blood sampling, sex, fasting status, country of birth, and socioeconomic status

[b]Statistically significant estimates are highlighted with bold

© 2024 Chourpiliadis C et al. *JAMA Network Open.*

eTable 7. Number (%) of participants with diagnosis of depression, anxiety and stress-related disorders among individuals with low, high or missing socioeconomic status.

Socioeconomic status	Depression, Anxiety or Stress-related disorders	Depression	Anxiety	Stress-related disorders
Low income	8 166 (8.5%)	4 896 (5.1%)	3 962 (4.1%)	2 331 (2.4%)
High income	5 967 (6.7%)	3 635 (4.1%)	2 579 (2.9%)	1 735 (1.9%)
Missing	2 251 (9.0%)	1 274 (5.1%)	1 092 (4.4%)	816 (3.3%)

© 2024 Chourpiliadis C et al. *JAMA Network Open.*

eTable 8. Incidence rates (IR) per 10 000 person-years and adjusted hazard ratios (aHR) with 95% confidence intervals (CI) of depression, anxiety, or stress-related disorders in relation to high versus low levels of carbohydrate, lipid, and apolipoprotein biomarkers, excluding from the analysis individuals missing socioeconomic status – a study based on AMORIS Cohort

**Biomarkers[a,b]**	**Depression, anxiety, or stress-related disorders**		**Depression**		**Anxiety**		**Stress-related disorders**	
	**No. of events (IR)**	**aHR (95% CI)**	**No. of events (IR)**	**aHR (95% CI)**	**No. of events (IR)**	**aHR (95% CI)**	**No. of events (IR)**	**aHR (95% CI)**
**High glucose (≥6.11 mmol/L)**								
**Yes**	549 (40.2)	**1.30 (1.19-1.42)**	373 (27.0)	**1.37 (1.24-1.53)**	226 (16.2)	**1.20 (1.05-1.37)**	109 (7.8)	1.21 (1.00-1.47)
**No**	11 552 (34.8)	Ref.	6 879 (20.5)	Ref.	5 408 (16.0)	Ref.	3 446 (10.2)	Ref.
**High total cholesterol (≥5.00 mmol/L)**								
**Yes**	8 013 (34.2)	1.02 (0.98-1.07)	4 965 (20.9)	1.01 (0.96-1.07)	3 692 (15.5)	1.06 (1.00-1.12)	1 949 (8.1)	1.00 (0.93-1.07)
**No**	4 537 (36.5)	Ref.	2 580 (20.5)	Ref.	2 146 (16.9)	Ref.	1 709 (13.4)	Ref.
**High triglycerides (≥1.71 mmol/L)**								
**Yes**	2 199 (35.0)	**1.15 (1.10-1.21)**	1 427 (22.5)	**1.19 (1.12-1.26)**	973 (15.2)	**1.13 (1.05-1.21)**	550 (8.6)	**1.21 (1.10-1.33)**
**No**	10 282 (34.9)	Ref.	6 080 (20.4)	Ref.	4 833 (16.1)	Ref.	3 090 (10.3)	Ref.
**High LDL-C (≥3.00 mmol/L)**								
**Yes**	3 534 (32.7)	1.00 (0.94-1.06)	2 209 (20.2)	0.98 (0.91-1.06)	1 609 (14.6)	0.98 (0.90-1.07)	824 (7.5)	0.98 (0.88-1.09)
**No**	1 870 (35.8)	Ref.	1 085 (20.5)	Ref.	908 (17.1)	Ref.	671 (12.6)	Ref.
**High HDL-C (≥1.03 mmol/L)**								
**Yes**	5 249 (33.6)	0.93 (0.83-1.03)	3 162 (20.0)	0.95 (0.83-1.09)	2 820 (16.0)	**0.83 (0.71-0.96)**	1 481 (9.3)	0.88 (0.71-1.07)
**No**	372 (33.2)	Ref.	226 (18.6)	Ref.	228 (16.6)	Ref.	102 (8.84)	Ref.
**High LDL-C/HDL-C (≥3.50)**								
**Yes**	615 (30.0)	1.02 (0.94-1.12)	390 (18.8)	1.00 (0.90-1.12)	291 (14.0)	1.09 (0.96-1.24)	115 (5.5)	0.90 (0.74-1.09)
**No**	4 557 (34.2)	Ref.	2 737 (20.3)	Ref.	2 131 (15.7)	Ref.	1 336 (9.8)	Ref.
**High ApoA-I (≥1.00 mmol/L in male and 1.10 mmol/L in female)**								
**Yes**	4 580 (33.1)	0.94 (0.78-1.13)	2 773 (19.8)	0.84 (0.67-1.06)	2 181 (15.5)	1.08 (0.81-1.42)	1 258 (8.9)	0.86 (0.63-1.18)
**No**	117 (35.8)	Ref.	77 (23.4)	Ref.	50 (15.0)	Ref.	40 (12.0)	Ref.
**High ApoB (≥0.90 mmol/L)**								
**Yes**	3 168 (31.8)	0.94 (0.86-1.02)	1 935 (19.2)	0.92 (0.82-1.02)	1 505 (14.9)	1.02 (0.91-1.15)	782 (7.7)	0.87 (0.76-1.00)
**No**	811 (36.1)	Ref.	458 (20.1)	Ref.	374 (16.3)	Ref.	313 (13.6)	Ref.
**High ApoB/ApoA-I (≥0.90 in male and 0.80 in female)**								
**Yes**	1 425 (31.6)	1.04 (0.97-1.12)	886 (19.5)	1.03 (0.94-1.13)	688 (15.0)	1.11 (1.00-1.23)	308 (6.7)	0.97 (0.84-1.11)
**No**	1 881 (32.0)	Ref.	1 108 (18.7)	Ref.	892 (15.0)	Ref.	586 (9.8)	Ref.

Abbreviations: ApoA-I: apolipoprotein A-I; ApoB: apolipoprotein B; HDL-C: high-density lipoprotein cholesterol; LDL-C: low-density lipoprotein cholesterol; Ref.: Reference

[a]Adjusted for age at first blood sampling, sex, fasting status, country of birth, and socioeconomic status

[b]Statistically significant estimates are highlighted with bold

© 2024 Chourpiliadis C et al. *JAMA Network Open.*

eTable 9. Observed and predicted biomarker levels among cases and controls during up to 30 years before diagnosis of the cases and their matched controls

Depression, Anxiety or Stress-related disorders												
Glucose												
	Cases						Controls					
	Observed			Predicted			Observed			Predicted		
Time since index date	Number	Mean	SD	Time since index date	Mean	SE	Number	Mean	SD	Time since index date	Mean	SE
0	64	5,44	1,93	0,00	5,65	0,13	660	5,15	1,05	0,00	5,12	0,02
1	101	5,94	2,61	0,61	5,63	0,10	941	5,08	0,96	0,61	5,11	0,02
2	139	5,51	1,99	1,22	5,60	0,09	1186	5,09	1,16	1,22	5,10	0,01
3	158	5,45	1,62	1,84	5,57	0,08	1523	5,08	1,17	1,84	5,09	0,01
4	211	5,58	2,21	2,45	5,54	0,07	2323	5,01	0,99	2,45	5,08	0,01
5	501	5,37	1,92	3,06	5,50	0,07	3726	5,04	1,14	3,06	5,07	0,01
6	544	5,20	1,51	3,67	5,47	0,06	5498	5,04	1,21	3,67	5,06	0,01
7	703	5,24	1,69	4,29	5,43	0,06	6547	5,05	1,17	4,29	5,05	0,01
8	733	5,19	1,54	4,90	5,39	0,05	7440	5,01	1,14	4,90	5,05	0,01
9	850	5,22	1,66	5,51	5,35	0,05	8468	5,02	1,13	5,51	5,04	0,01
10	979	5,10	1,44	6,12	5,32	0,04	9790	4,99	1,10	6,12	5,03	0,01
11	1072	5,00	1,15	6,73	5,28	0,03	10938	4,97	1,11	6,73	5,02	0,01
12	1255	5,02	1,35	7,35	5,25	0,03	12075	4,96	1,14	7,35	5,01	0,01
13	1320	5,07	1,38	7,96	5,22	0,03	13057	4,94	1,07	7,96	5,00	0,01
14	1424	4,99	1,25	8,57	5,18	0,02	13900	4,92	1,09	8,57	5,00	0,01
15	1522	5,01	1,23	9,18	5,15	0,02	14341	4,90	1,03	9,18	4,99	0,00
16	1522	4,95	1,37	9,80	5,13	0,02	15150	4,90	1,08	9,80	4,98	0,00
17	1618	4,92	1,02	10,41	5,10	0,02	15035	4,90	1,11	10,41	4,97	0,00
18	1576	4,90	0,93	11,02	5,08	0,02	15414	4,89	1,01	11,02	4,97	0,00
19	1626	4,92	1,14	11,63	5,05	0,02	15433	4,88	1,01	11,63	4,96	0,00
20	1553	4,89	1,02	12,24	5,03	0,02	15026	4,87	1,00	12,24	4,95	0,00
21	1526	4,93	1,07	12,86	5,02	0,01	14918	4,86	0,95	12,86	4,95	0,00
22	1443	4,90	1,02	13,47	5,00	0,01	14275	4,85	0,93	13,47	4,94	0,00
23	1471	4,89	0,96	14,08	4,98	0,01	13816	4,84	0,85	14,08	4,93	0,00
24	1269	4,85	0,99	14,69	4,97	0,01	12613	4,83	0,93	14,69	4,92	0,00
25	1085	4,90	1,35	15,31	4,96	0,01	11194	4,80	0,89	15,31	4,92	0,00
26	1002	4,73	0,68	15,92	4,95	0,01	9454	4,78	0,87	15,92	4,91	0,00
27	847	4,73	0,74	16,53	4,94	0,01	8135	4,74	0,83	16,53	4,90	0,00
28	761	4,77	0,79	17,14	4,93	0,01	6697	4,74	0,87	17,14	4,90	0,00
29	581	4,71	0,57	17,76	4,92	0,01	5444	4,74	0,91	17,76	4,89	0,00
30	473	4,70	0,72	18,37	4,92	0,01	4256	4,71	0,72	18,37	4,89	0,00
				18,98	4,91	0,01				18,98	4,88	0,00
				19,59	4,91	0,01				19,59	4,87	0,00
				20,20	4,90	0,01				20,20	4,87	0,00
				20,82	4,89	0,01				20,82	4,86	0,00
				21,43	4,89	0,01				21,43	4,85	0,00
				22,04	4,88	0,01				22,04	4,84	0,00
				22,65	4,87	0,01				22,65	4,84	0,00
				23,27	4,87	0,02				23,27	4,83	0,00
				23,88	4,86	0,02				23,88	4,82	0,00
				24,49	4,85	0,02				24,49	4,81	0,00
				25,10	4,84	0,02				25,10	4,80	0,00
				25,71	4,82	0,02				25,71	4,79	0,00
				26,33	4,81	0,02				26,33	4,78	0,00
				26,94	4,79	0,02				26,94	4,77	0,00
				27,55	4,77	0,02				27,55	4,76	0,00
				28,16	4,75	0,02				28,16	4,74	0,00
				28,78	4,73	0,03				28,78	4,73	0,00
				29,39	4,70	0,03				29,39	4,71	0,00
				30,00	4,67	0,04				30,00	4,70	0,01

© 2024 Chourpiliadis C et al. *JAMA Network Open.*

Depression, Anxiety or Stress-related disorders												
Total Cholesterol												
	Cases						Controls					
	Observed			Predicted			Observed			Predicted		
Time since index date	Number	Mean	SD	Time since index date	Mean	SE	Number	Mean	SD	Time since index date	Mean	SE
0	75	6,35	1,50	0,00	6,19	0,07	689	5,76	1,05	0,00	5,76	0,01
1	102	5,90	1,05	0,61	6,12	0,05	987	5,71	1,06	0,61	5,75	0,01
2	148	5,95	1,07	1,22	6,06	0,04	1249	5,76	1,12	1,22	5,74	0,01
3	162	5,84	1,05	1,84	6,00	0,04	1596	5,74	1,13	1,84	5,73	0,01
4	225	5,97	1,18	2,45	5,95	0,03	2481	5,69	1,11	2,45	5,72	0,01
5	530	5,80	1,14	3,06	5,91	0,03	3944	5,63	1,11	3,06	5,70	0,01
6	561	5,69	1,13	3,67	5,87	0,03	5806	5,61	1,12	3,67	5,69	0,01
7	746	5,75	1,23	4,29	5,83	0,03	6941	5,62	1,16	4,29	5,68	0,01
8	763	5,77	1,22	4,90	5,80	0,03	7855	5,62	1,16	4,90	5,67	0,01
9	899	5,65	1,20	5,51	5,77	0,03	8967	5,60	1,16	5,51	5,65	0,01
10	1025	5,67	1,17	6,12	5,75	0,03	10362	5,60	1,15	6,12	5,64	0,01
11	1142	5,65	1,08	6,73	5,73	0,03	11611	5,57	1,14	6,73	5,63	0,01
12	1344	5,58	1,18	7,35	5,71	0,02	12803	5,56	1,14	7,35	5,62	0,00
13	1398	5,63	1,21	7,96	5,69	0,02	13825	5,55	1,13	7,96	5,61	0,00
14	1500	5,58	1,21	8,57	5,67	0,02	14655	5,54	1,13	8,57	5,60	0,00
15	1618	5,58	1,16	9,18	5,66	0,02	15137	5,54	1,13	9,18	5,59	0,00
16	1592	5,59	1,15	9,80	5,65	0,02	15960	5,54	1,14	9,80	5,58	0,00
17	1713	5,55	1,15	10,41	5,64	0,01	15877	5,55	1,15	10,41	5,58	0,00
18	1663	5,51	1,13	11,02	5,63	0,01	16239	5,53	1,14	11,02	5,57	0,00
19	1710	5,50	1,12	11,63	5,62	0,01	16189	5,54	1,15	11,63	5,56	0,00
20	1620	5,58	1,12	12,24	5,61	0,01	15852	5,55	1,15	12,24	5,56	0,00
21	1612	5,56	1,11	12,86	5,60	0,01	15700	5,53	1,12	12,86	5,55	0,00
22	1512	5,56	1,14	13,47	5,59	0,01	14969	5,54	1,12	13,47	5,55	0,00
23	1539	5,51	1,15	14,08	5,59	0,01	14548	5,55	1,14	14,08	5,55	0,00
24	1334	5,55	1,13	14,69	5,58	0,01	13213	5,53	1,13	14,69	5,54	0,00
25	1137	5,58	1,14	15,31	5,58	0,01	11747	5,53	1,14	15,31	5,54	0,00
26	1039	5,47	1,11	15,92	5,57	0,01	9906	5,52	1,12	15,92	5,54	0,00
27	870	5,52	1,13	16,53	5,57	0,01	8504	5,53	1,10	16,53	5,54	0,00
28	789	5,60	1,17	17,14	5,56	0,01	7002	5,54	1,14	17,14	5,54	0,00
29	604	5,51	1,09	17,76	5,56	0,01	5689	5,52	1,11	17,76	5,54	0,00
30	486	5,45	1,09	18,37	5,56	0,01	4421	5,45	1,09	18,37	5,54	0,00
				18,98	5,55	0,01				18,98	5,54	0,00
				19,59	5,55	0,01				19,59	5,54	0,00
				20,20	5,55	0,01				20,20	5,54	0,00
				20,82	5,55	0,01				20,82	5,54	0,00
				21,43	5,55	0,01				21,43	5,54	0,00
				22,04	5,54	0,01				22,04	5,54	0,00
				22,65	5,54	0,01				22,65	5,54	0,00
				23,27	5,54	0,02				23,27	5,54	0,00
				23,88	5,54	0,02				23,88	5,54	0,00
				24,49	5,54	0,02				24,49	5,54	0,00
				25,10	5,54	0,02				25,10	5,54	0,00
				25,71	5,53	0,02				25,71	5,54	0,00
				26,33	5,53	0,02				26,33	5,53	0,00
				26,94	5,53	0,02				26,94	5,53	0,00
				27,55	5,52	0,02				27,55	5,52	0,00
				28,16	5,51	0,02				28,16	5,51	0,00
				28,78	5,51	0,03				28,78	5,50	0,00
				29,39	5,50	0,04				29,39	5,49	0,00
				30,00	5,49	0,05				30,00	5,48	0,00

© 2024 Chourpiliadis C et al. *JAMA Network Open.*

Depression, Anxiety or Stress-related disorders												
Log2 Triglycerides												
	Cases						Controls					
	Observed			Predicted			Observed			Predicted		
Time since index date	Number	Mean	SD	Time since index date	Mean	SE	Number	Mean	SD	Time since index date	Mean	SE
0	73	0,53	1,02	0,00	0,52	0,02	686	0,21	0,79	0,00	0,19	0,02
1	100	0,46	0,99	0,61	0,48	0,01	982	0,20	0,82	0,61	0,19	0,01
2	148	0,44	0,89	1,22	0,45	0,01	1245	0,14	0,84	1,22	0,19	0,01
3	161	0,35	0,99	1,84	0,42	0,01	1590	0,18	0,83	1,84	0,19	0,01
4	224	0,32	0,84	2,45	0,39	0,01	2468	0,18	0,81	2,45	0,19	0,01
5	527	0,25	0,84	3,06	0,37	0,01	3926	0,17	0,82	3,06	0,18	0,01
6	559	0,25	0,84	3,67	0,34	0,01	5775	0,18	0,83	3,67	0,18	0,01
7	744	0,27	0,83	4,29	0,32	0,01	6905	0,16	0,82	4,29	0,18	0,01
8	760	0,26	0,82	4,90	0,30	0,01	7819	0,15	0,81	4,90	0,17	0,01
9	887	0,21	0,81	5,51	0,29	0,01	8919	0,15	0,81	5,51	0,17	0,01
10	1020	0,24	0,83	6,12	0,27	0,01	10311	0,14	0,82	6,12	0,16	0,00
11	1134	0,26	0,85	6,73	0,26	0,01	11540	0,13	0,82	6,73	0,16	0,00
12	1325	0,17	0,84	7,35	0,25	0,01	12738	0,11	0,81	7,35	0,15	0,00
13	1383	0,19	0,85	7,96	0,24	0,01	13746	0,10	0,82	7,96	0,15	0,00
14	1490	0,18	0,84	8,57	0,23	0,01	14578	0,09	0,81	8,57	0,14	0,00
15	1602	0,11	0,79	9,18	0,22	0,01	15075	0,09	0,81	9,18	0,14	0,00
16	1579	0,14	0,82	9,80	0,21	0,01	15899	0,08	0,81	9,80	0,13	0,00
17	1697	0,13	0,84	10,41	0,20	0,01	15780	0,08	0,81	10,41	0,13	0,00
18	1649	0,07	0,80	11,02	0,19	0,01	16169	0,08	0,81	11,02	0,12	0,00
19	1692	0,10	0,79	11,63	0,18	0,01	16129	0,07	0,80	11,63	0,12	0,00
20	1614	0,12	0,86	12,24	0,18	0,01	15757	0,07	0,82	12,24	0,11	0,00
21	1603	0,12	0,83	12,86	0,17	0,01	15604	0,07	0,79	12,86	0,11	0,00
22	1509	0,11	0,81	13,47	0,17	0,01	14902	0,06	0,80	13,47	0,10	0,00
23	1529	0,11	0,82	14,08	0,16	0,01	14479	0,07	0,80	14,08	0,10	0,00
24	1333	0,08	0,78	14,69	0,15	0,01	13162	0,05	0,80	14,69	0,09	0,00
25	1135	0,10	0,82	15,31	0,15	0,01	11701	0,04	0,79	15,31	0,09	0,00
26	1036	0,07	0,82	15,92	0,14	0,01	9875	0,03	0,79	15,92	0,09	0,00
27	869	0,04	0,84	16,53	0,14	0,01	8477	0,02	0,79	16,53	0,08	0,00
28	788	0,04	0,82	17,14	0,14	0,01	6976	0,01	0,79	17,14	0,08	0,00
29	604	-0,03	0,75	17,76	0,13	0,01	5674	0,02	0,79	17,76	0,08	0,00
30	485	-0,02	0,75	18,37	0,13	0,01	4414	-0,02	0,76	18,37	0,08	0,00
				18,98	0,12	0,01				18,98	0,07	0,00
				19,59	0,12	0,01				19,59	0,07	0,00
				20,20	0,11	0,01				20,20	0,07	0,00
				20,82	0,11	0,01				20,82	0,07	0,00
				21,43	0,11	0,01				21,43	0,06	0,00
				22,04	0,10	0,01				22,04	0,06	0,00
				22,65	0,10	0,01				22,65	0,06	0,00
				23,27	0,09	0,01				23,27	0,06	0,00
				23,88	0,09	0,01				23,88	0,05	0,00
				24,49	0,08	0,01				24,49	0,05	0,00
				25,10	0,07	0,01				25,10	0,04	0,00
				25,71	0,06	0,01				25,71	0,04	0,00
				26,33	0,06	0,01				26,33	0,03	0,00
				26,94	0,05	0,01				26,94	0,03	0,00
				27,55	0,03	0,01				27,55	0,02	0,00
				28,16	0,02	0,01				28,16	0,01	0,00
				28,78	0,01	0,01				28,78	0,01	0,00
				29,39	-0,01	0,01				29,39	-0,01	0,00
				30,00	-0,03	0,01				30,00	-0,02	0,01

© 2024 Chourpiliadis C et al. *JAMA Network Open.*

Depression, Anxiety or Stress-related disorders												
LDL-C												
	Cases						Controls					
	Observed			Predicted			Observed			Predicted		
Time since index date	Number	Mean	SD	Time since index date	Mean	SE	Number	Mean	SD	Time since index date	Mean	SE
0	37	4,02	1,16	0,00	3,85	0,07	286	3,74	1,00	0,00	3,75	0,02
1	51	3,54	1,10	0,61	3,83	0,05	413	3,75	0,97	0,61	3,74	0,01
2	63	3,74	1,06	1,22	3,80	0,04	497	3,71	1,05	1,22	3,72	0,01
3	81	3,76	1,10	1,84	3,78	0,03	646	3,71	1,07	1,84	3,71	0,01
4	91	3,93	1,06	2,45	3,77	0,03	979	3,64	1,03	2,45	3,69	0,01
5	198	3,79	1,09	3,06	3,75	0,03	1494	3,59	1,04	3,06	3,68	0,01
6	214	3,61	1,08	3,67	3,74	0,03	2187	3,60	1,04	3,67	3,67	0,01
7	289	3,66	1,08	4,29	3,73	0,03	2631	3,67	1,09	4,29	3,66	0,01
8	272	3,67	1,13	4,90	3,72	0,03	2881	3,65	1,07	4,90	3,65	0,01
9	336	3,63	1,10	5,51	3,71	0,03	3343	3,61	1,03	5,51	3,64	0,01
10	384	3,67	1,12	6,12	3,70	0,03	3912	3,62	1,07	6,12	3,63	0,01
11	393	3,65	1,00	6,73	3,69	0,03	4291	3,59	1,06	6,73	3,63	0,01
12	475	3,62	1,09	7,35	3,69	0,03	4588	3,60	1,07	7,35	3,62	0,01
13	495	3,72	1,06	7,96	3,68	0,02	4924	3,58	1,07	7,96	3,62	0,01
14	454	3,65	1,15	8,57	3,68	0,02	5066	3,59	1,08	8,57	3,61	0,01
15	524	3,66	1,11	9,18	3,67	0,02	5307	3,59	1,07	9,18	3,61	0,01
16	545	3,64	1,07	9,80	3,66	0,02	5430	3,61	1,07	9,80	3,61	0,01
17	546	3,62	1,05	10,41	3,66	0,01	5356	3,59	1,08	10,41	3,60	0,01
18	552	3,55	1,12	11,02	3,65	0,01	5385	3,58	1,06	11,02	3,60	0,01
19	551	3,52	1,05	11,63	3,65	0,01	5458	3,59	1,08	11,63	3,60	0,00
20	517	3,58	1,07	12,24	3,64	0,01	5175	3,60	1,08	12,24	3,60	0,00
21	528	3,57	1,07	12,86	3,64	0,01	5040	3,60	1,05	12,86	3,60	0,00
22	487	3,58	1,08	13,47	3,64	0,01	4819	3,58	1,07	13,47	3,60	0,00
23	503	3,49	1,01	14,08	3,63	0,01	4605	3,58	1,09	14,08	3,59	0,00
24	420	3,57	1,07	14,69	3,63	0,01	3957	3,57	1,09	14,69	3,59	0,00
25	314	3,58	1,09	15,31	3,62	0,01	3442	3,56	1,08	15,31	3,59	0,00
26	299	3,40	0,98	15,92	3,62	0,01	2819	3,55	1,08	15,92	3,59	0,00
27	254	3,59	1,11	16,53	3,61	0,01	2327	3,54	1,05	16,53	3,59	0,00
28	216	3,51	1,08	17,14	3,60	0,01	1866	3,59	1,12	17,14	3,59	0,00
29	167	3,51	1,09	17,76	3,60	0,01	1438	3,52	1,06	17,76	3,59	0,00
30	99	3,37	1,18	18,37	3,59	0,01	1076	3,47	1,02	18,37	3,59	0,00
				18,98	3,59	0,01				18,98	3,59	0,00
				19,59	3,58	0,01				19,59	3,59	0,00
				20,20	3,58	0,01				20,20	3,59	0,00
				20,82	3,57	0,02				20,82	3,59	0,00
				21,43	3,57	0,02				21,43	3,59	0,00
				22,04	3,56	0,02				22,04	3,59	0,00
				22,65	3,55	0,02				22,65	3,58	0,00
				23,27	3,55	0,02				23,27	3,58	0,00
				23,88	3,54	0,03				23,88	3,58	0,00
				24,49	3,53	0,03				24,49	3,57	0,01
				25,10	3,53	0,03				25,10	3,57	0,01
				25,71	3,52	0,03				25,71	3,56	0,01
				26,33	3,51	0,03				26,33	3,56	0,01
				26,94	3,50	0,03				26,94	3,55	0,01
				27,55	3,49	0,03				27,55	3,54	0,01
				28,16	3,48	0,04				28,16	3,53	0,01
				28,78	3,47	0,04				28,78	3,52	0,01
				29,39	3,45	0,05				29,39	3,51	0,02
				30,00	3,44	0,07				30,00	3,49	0,02

© 2024 Chourpiliadis C et al. *JAMA Network Open.*

Depression, Anxiety or Stress-related disorders												
HDL-C												
	Cases						Controls					
	Observed			Predicted			Observed			Predicted		
Time since index date	Number	Mean	SD	Time since index date	Mean	SE	Number	Mean	SD	Time since index date	Mean	SE
0	16	1,50	0,27	0,00	1,53	0,02	196	1,55	0,37	0,00	1,56	0,01
1	25	1,58	0,39	0,61	1,54	0,02	253	1,56	0,43	0,61	1,56	0,01
2	27	1,55	0,38	1,22	1,54	0,02	336	1,58	0,38	1,22	1,56	0,01
3	46	1,57	0,40	1,84	1,55	0,01	434	1,56	0,38	1,84	1,57	0,01
4	51	1,60	0,47	2,45	1,55	0,01	703	1,59	0,39	2,45	1,57	0,01
5	150	1,55	0,37	3,06	1,56	0,01	1151	1,58	0,39	3,06	1,57	0,01
6	177	1,49	0,40	3,67	1,56	0,01	1772	1,54	0,37	3,67	1,57	0,01
7	239	1,55	0,40	4,29	1,56	0,01	2129	1,55	0,38	4,29	1,57	0,01
8	221	1,57	0,40	4,90	1,56	0,01	2410	1,56	0,38	4,90	1,56	0,01
9	248	1,57	0,40	5,51	1,56	0,01	2786	1,56	0,39	5,51	1,56	0,01
10	298	1,55	0,39	6,12	1,56	0,01	3235	1,56	0,38	6,12	1,56	0,01
11	323	1,55	0,39	6,73	1,56	0,01	3533	1,57	0,39	6,73	1,56	0,00
12	366	1,56	0,38	7,35	1,56	0,01	3840	1,56	0,37	7,35	1,56	0,00
13	385	1,56	0,38	7,96	1,56	0,01	4103	1,58	0,38	7,96	1,56	0,00
14	346	1,53	0,38	8,57	1,56	0,01	4305	1,57	0,38	8,57	1,56	0,00
15	409	1,57	0,37	9,18	1,55	0,01	4430	1,58	0,40	9,18	1,56	0,00
16	426	1,55	0,37	9,80	1,55	0,01	4557	1,58	0,37	9,80	1,56	0,00
17	436	1,54	0,36	10,41	1,55	0,01	4493	1,58	0,38	10,41	1,56	0,00
18	443	1,59	0,38	11,02	1,55	0,01	4543	1,58	0,39	11,02	1,56	0,00
19	469	1,59	0,37	11,63	1,55	0,00	4496	1,58	0,38	11,63	1,57	0,00
20	411	1,58	0,42	12,24	1,55	0,00	4328	1,59	0,38	12,24	1,57	0,00
21	422	1,55	0,39	12,86	1,55	0,01	4232	1,58	0,37	12,86	1,57	0,00
22	370	1,60	0,36	13,47	1,55	0,01	4008	1,58	0,38	13,47	1,57	0,00
23	398	1,58	0,39	14,08	1,55	0,01	3821	1,58	0,37	14,08	1,57	0,00
24	324	1,61	0,37	14,69	1,55	0,01	3275	1,60	0,38	14,69	1,57	0,00
25	267	1,59	0,37	15,31	1,55	0,01	2847	1,59	0,38	15,31	1,57	0,00
26	236	1,64	0,36	15,92	1,56	0,01	2293	1,61	0,38	15,92	1,57	0,00
27	196	1,61	0,39	16,53	1,56	0,01	1915	1,61	0,37	16,53	1,57	0,00
28	175	1,62	0,35	17,14	1,56	0,01	1527	1,60	0,38	17,14	1,58	0,00
29	138	1,68	0,36	17,76	1,56	0,01	1216	1,59	0,36	17,76	1,58	0,00
30	78	1,63	0,35	18,37	1,56	0,01	915	1,60	0,35	18,37	1,58	0,00
				18,98	1,57	0,01				18,98	1,58	0,00
				19,59	1,57	0,01				19,59	1,58	0,00
				20,20	1,58	0,01				20,20	1,58	0,00
				20,82	1,58	0,01				20,82	1,58	0,00
				21,43	1,58	0,01				21,43	1,59	0,00
				22,04	1,59	0,01				22,04	1,59	0,00
				22,65	1,59	0,01				22,65	1,59	0,00
				23,27	1,60	0,01				23,27	1,59	0,00
				23,88	1,60	0,01				23,88	1,59	0,00
				24,49	1,61	0,01				24,49	1,59	0,00
				25,10	1,61	0,01				25,10	1,59	0,00
				25,71	1,62	0,01				25,71	1,60	0,00
				26,33	1,62	0,01				26,33	1,60	0,00
				26,94	1,63	0,01				26,94	1,60	0,00
				27,55	1,63	0,01				27,55	1,60	0,00
				28,16	1,64	0,01				28,16	1,60	0,00
				28,78	1,64	0,01				28,78	1,60	0,00
				29,39	1,65	0,02				29,39	1,60	0,00
				30,00	1,65	0,02				30,00	1,60	0,00

© 2024 Chourpiliadis C et al. *JAMA Network Open.*

Depression, Anxiety or Stress-related disorders												
ApoA-I												
	Cases						Controls					
	Observed			Predicted			Observed			Predicted		
Time since index date	Number	Mean	SD	Time since index date	Mean	SE	Number	Mean	SD	Time since index date	Mean	SE
0	31	1,50	0,27	0,00	1,52	0,01	254	1,43	0,21	0,00	1,42	0,01
1	42	1,53	0,25	0,61	1,51	0,01	365	1,41	0,22	0,61	1,43	0,01
2	58	1,49	0,19	1,22	1,50	0,01	438	1,44	0,23	1,22	1,43	0,01
3	78	1,48	0,23	1,84	1,49	0,01	595	1,43	0,22	1,84	1,43	0,01
4	78	1,49	0,29	2,45	1,48	0,01	883	1,43	0,23	2,45	1,43	0,00
5	185	1,44	0,27	3,06	1,47	0,01	1308	1,43	0,24	3,06	1,43	0,00
6	180	1,43	0,24	3,67	1,47	0,01	1869	1,41	0,22	3,67	1,43	0,00
7	233	1,43	0,24	4,29	1,46	0,01	2225	1,42	0,22	4,29	1,43	0,00
8	220	1,42	0,22	4,90	1,45	0,01	2437	1,42	0,22	4,90	1,43	0,00
9	269	1,45	0,23	5,51	1,45	0,01	2881	1,42	0,22	5,51	1,43	0,00
10	318	1,42	0,25	6,12	1,44	0,01	3377	1,42	0,22	6,12	1,42	0,00
11	325	1,43	0,23	6,73	1,44	0,01	3732	1,42	0,22	6,73	1,42	0,00
12	410	1,41	0,23	7,35	1,44	0,00	4021	1,42	0,22	7,35	1,42	0,00
13	418	1,44	0,22	7,96	1,43	0,00	4273	1,43	0,22	7,96	1,42	0,00
14	404	1,43	0,24	8,57	1,43	0,00	4537	1,42	0,22	8,57	1,42	0,00
15	467	1,43	0,23	9,18	1,43	0,00	4758	1,42	0,23	9,18	1,42	0,00
16	497	1,43	0,23	9,80	1,43	0,00	4899	1,42	0,22	9,80	1,42	0,00
17	497	1,41	0,21	10,41	1,43	0,00	4821	1,43	0,22	10,41	1,42	0,00
18	478	1,42	0,22	11,02	1,42	0,00	4853	1,42	0,22	11,02	1,42	0,00
19	516	1,43	0,22	11,63	1,42	0,00	4882	1,42	0,22	11,63	1,42	0,00
20	480	1,43	0,25	12,24	1,42	0,00	4691	1,43	0,22	12,24	1,42	0,00
21	496	1,44	0,24	12,86	1,42	0,00	4550	1,42	0,22	12,86	1,42	0,00
22	442	1,43	0,22	13,47	1,42	0,00	4297	1,42	0,22	13,47	1,42	0,00
23	463	1,42	0,23	14,08	1,42	0,00	4176	1,42	0,21	14,08	1,42	0,00
24	383	1,43	0,23	14,69	1,42	0,00	3641	1,42	0,22	14,69	1,42	0,00
25	304	1,44	0,22	15,31	1,42	0,00	3258	1,42	0,22	15,31	1,42	0,00
26	294	1,44	0,22	15,92	1,42	0,00	2747	1,43	0,22	15,92	1,42	0,00
27	255	1,44	0,23	16,53	1,42	0,00	2321	1,43	0,22	16,53	1,42	0,00
28	214	1,43	0,21	17,14	1,43	0,00	1859	1,41	0,23	17,14	1,42	0,00
29	167	1,44	0,22	17,76	1,43	0,00	1441	1,41	0,22	17,76	1,42	0,00
30	99	1,41	0,24	18,37	1,43	0,00	1076	1,42	0,20	18,37	1,42	0,00
				18,98	1,43	0,00				18,98	1,42	0,00
				19,59	1,43	0,00				19,59	1,42	0,00
				20,20	1,43	0,00				20,20	1,42	0,00
				20,82	1,43	0,00				20,82	1,42	0,00
				21,43	1,43	0,00				21,43	1,42	0,00
				22,04	1,43	0,00				22,04	1,42	0,00
				22,65	1,44	0,00				22,65	1,42	0,00
				23,27	1,44	0,00				23,27	1,42	0,00
				23,88	1,44	0,00				23,88	1,42	0,00
				24,49	1,44	0,00				24,49	1,42	0,00
				25,10	1,44	0,00				25,10	1,42	0,00
				25,71	1,44	0,00				25,71	1,42	0,00
				26,33	1,44	0,00				26,33	1,42	0,00
				26,94	1,44	0,00				26,94	1,42	0,00
				27,55	1,44	0,00				27,55	1,42	0,00
				28,16	1,43	0,00				28,16	1,42	0,00
				28,78	1,43	0,00				28,78	1,42	0,00
				29,39	1,43	0,00				29,39	1,42	0,00
				30,00	1,43	0,01				30,00	1,42	0,00

© 2024 Chourpiliadis C et al. *JAMA Network Open.*

Depression, Anxiety or Stress-related disorders												
ApoB												
	Cases						Controls					
	Observed			Predicted			Observed			Predicted		
Time since index date	Number	Mean	SD	Time since index date	Mean	SE	Number	Mean	SD	Time since index date	Mean	SE
0	33	1,42	0,40	0,00	1,44	0,02	268	1,18	0,32	0,00	1,20	0,01
1	42	1,35	0,42	0,61	1,41	0,02	366	1,21	0,34	0,61	1,20	0,00
2	56	1,40	0,36	1,22	1,38	0,02	452	1,20	0,38	1,22	1,19	0,00
3	73	1,33	0,42	1,84	1,35	0,01	601	1,21	0,36	1,84	1,19	0,00
4	72	1,34	0,38	2,45	1,33	0,01	843	1,19	0,35	2,45	1,19	0,00
5	184	1,23	0,38	3,06	1,31	0,01	1228	1,17	0,35	3,06	1,19	0,00
6	162	1,18	0,36	3,67	1,29	0,01	1775	1,17	0,35	3,67	1,19	0,00
7	214	1,19	0,37	4,29	1,28	0,01	2162	1,18	0,38	4,29	1,18	0,00
8	198	1,21	0,41	4,90	1,26	0,01	2346	1,17	0,38	4,90	1,18	0,00
9	264	1,19	0,36	5,51	1,25	0,01	2872	1,16	0,36	5,51	1,18	0,00
10	298	1,19	0,37	6,12	1,24	0,01	3263	1,16	0,36	6,12	1,18	0,00
11	311	1,22	0,38	6,73	1,23	0,01	3513	1,16	0,36	6,73	1,18	0,00
12	358	1,18	0,36	7,35	1,22	0,01	3768	1,17	0,36	7,35	1,17	0,00
13	384	1,20	0,36	7,96	1,21	0,01	3992	1,17	0,35	7,96	1,17	0,00
14	398	1,22	0,40	8,57	1,20	0,01	4233	1,18	0,36	8,57	1,17	0,00
15	411	1,21	0,36	9,18	1,20	0,01	4247	1,19	0,36	9,18	1,17	0,00
16	451	1,19	0,37	9,80	1,19	0,01	4338	1,19	0,36	9,80	1,17	0,00
17	467	1,18	0,35	10,41	1,19	0,01	4355	1,18	0,36	10,41	1,17	0,00
18	430	1,17	0,36	11,02	1,19	0,01	4496	1,18	0,35	11,02	1,17	0,00
19	470	1,18	0,36	11,63	1,19	0,01	4421	1,19	0,35	11,63	1,17	0,00
20	445	1,19	0,34	12,24	1,19	0,01	4285	1,19	0,35	12,24	1,17	0,00
21	462	1,17	0,35	12,86	1,18	0,00	4132	1,19	0,35	12,86	1,17	0,00
22	388	1,19	0,37	13,47	1,18	0,00	3891	1,19	0,35	13,47	1,17	0,00
23	396	1,20	0,34	14,08	1,18	0,00	3662	1,21	0,36	14,08	1,17	0,00
24	351	1,22	0,36	14,69	1,18	0,00	3221	1,20	0,36	14,69	1,17	0,00
25	273	1,22	0,35	15,31	1,18	0,00	2840	1,22	0,36	15,31	1,17	0,00
26	244	1,19	0,32	15,92	1,19	0,00	2334	1,24	0,36	15,92	1,18	0,00
27	215	1,26	0,36	16,53	1,19	0,00	1870	1,24	0,33	16,53	1,18	0,00
28	166	1,23	0,32	17,14	1,19	0,00	1371	1,27	0,35	17,14	1,18	0,00
29	107	1,21	0,30	17,76	1,19	0,00	1058	1,26	0,32	17,76	1,18	0,00
30	73	1,18	0,29	18,37	1,19	0,00	795	1,26	0,31	18,37	1,19	0,00
				18,98	1,19	0,00				18,98	1,19	0,00
				19,59	1,19	0,00				19,59	1,19	0,00
				20,20	1,20	0,00				20,20	1,19	0,00
				20,82	1,20	0,00				20,82	1,20	0,00
				21,43	1,20	0,00				21,43	1,20	0,00
				22,04	1,20	0,01				22,04	1,20	0,00
				22,65	1,20	0,01				22,65	1,21	0,00
				23,27	1,21	0,01				23,27	1,21	0,00
				23,88	1,21	0,01				23,88	1,22	0,00
				24,49	1,21	0,01				24,49	1,22	0,00
				25,10	1,21	0,01				25,10	1,23	0,00
				25,71	1,21	0,01				25,71	1,23	0,00
				26,33	1,21	0,01				26,33	1,23	0,00
				26,94	1,21	0,01				26,94	1,24	0,00
				27,55	1,21	0,01				27,55	1,24	0,00
				28,16	1,21	0,01				28,16	1,25	0,00
				28,78	1,21	0,01				28,78	1,25	0,00
				29,39	1,21	0,02				29,39	1,26	0,01
				30,00	1,21	0,02				30,00	1,27	0,01

© 2024 Chourpiliadis C et al. *JAMA Network Open.*

Depression, Anxiety or Stress-related disorders												
Log2 LDL-C/HDL-C ratio												
	Cases						Controls					
	Observed			Predicted			Observed			Predicted		
Time since index date	Number	Mean	SD	Time since index date	Mean	SE	Number	Mean	SD	Time since index date	Mean	SE
0	36	1,41	0,60	0,00	1,34	0,02	273	1,26	0,56	0,00	1,27	0,02
1	48	1,20	0,57	0,61	1,32	0,02	398	1,28	0,58	0,61	1,26	0,01
2	59	1,27	0,55	1,22	1,30	0,01	465	1,22	0,62	1,22	1,24	0,01
3	73	1,28	0,66	1,84	1,28	0,01	589	1,22	0,62	1,84	1,23	0,01
4	85	1,26	0,65	2,45	1,27	0,01	902	1,18	0,60	2,45	1,22	0,01
5	175	1,23	0,61	3,06	1,26	0,01	1377	1,16	0,60	3,06	1,22	0,01
6	199	1,22	0,57	3,67	1,25	0,01	2043	1,18	0,59	3,67	1,21	0,01
7	274	1,21	0,63	4,29	1,24	0,01	2464	1,22	0,61	4,29	1,20	0,01
8	250	1,23	0,63	4,90	1,23	0,01	2693	1,19	0,60	4,90	1,20	0,01
9	314	1,16	0,64	5,51	1,23	0,01	3144	1,19	0,59	5,51	1,19	0,01
10	350	1,22	0,61	6,12	1,22	0,01	3671	1,18	0,60	6,12	1,19	0,01
11	364	1,20	0,59	6,73	1,22	0,01	4055	1,16	0,60	6,73	1,18	0,01
12	456	1,22	0,62	7,35	1,21	0,01	4346	1,18	0,60	7,35	1,18	0,01
13	456	1,23	0,59	7,96	1,21	0,01	4638	1,14	0,61	7,96	1,18	0,01
14	427	1,18	0,61	8,57	1,21	0,01	4794	1,16	0,61	8,57	1,18	0,00
15	497	1,17	0,62	9,18	1,21	0,01	4972	1,15	0,63	9,18	1,17	0,00
16	517	1,19	0,60	9,80	1,20	0,01	5107	1,16	0,61	9,80	1,17	0,00
17	514	1,20	0,60	10,41	1,20	0,01	5049	1,15	0,61	10,41	1,17	0,00
18	514	1,11	0,63	11,02	1,20	0,01	5093	1,15	0,60	11,02	1,17	0,00
19	517	1,10	0,60	11,63	1,20	0,01	5149	1,15	0,61	11,63	1,17	0,00
20	483	1,15	0,65	12,24	1,19	0,01	4837	1,14	0,60	12,24	1,17	0,00
21	497	1,14	0,64	12,86	1,19	0,01	4772	1,15	0,60	12,86	1,16	0,00
22	460	1,12	0,59	13,47	1,19	0,01	4605	1,15	0,61	13,47	1,16	0,00
23	481	1,10	0,58	14,08	1,19	0,01	4422	1,15	0,62	14,08	1,16	0,00
24	406	1,14	0,59	14,69	1,18	0,01	3817	1,13	0,61	14,69	1,16	0,00
25	305	1,11	0,59	15,31	1,18	0,01	3309	1,14	0,61	15,31	1,16	0,00
26	283	1,02	0,57	15,92	1,18	0,01	2690	1,10	0,62	15,92	1,16	0,00
27	239	1,10	0,61	16,53	1,17	0,01	2223	1,11	0,60	16,53	1,16	0,00
28	206	1,07	0,55	17,14	1,17	0,01	1771	1,13	0,61	17,14	1,16	0,00
29	162	1,04	0,61	17,76	1,16	0,01	1377	1,11	0,61	17,76	1,15	0,00
30	91	0,98	0,65	18,37	1,16	0,01	1039	1,08	0,58	18,37	1,15	0,00
				18,98	1,15	0,01				18,98	1,15	0,00
				19,59	1,15	0,01				19,59	1,15	0,00
				20,20	1,14	0,01				20,20	1,15	0,00
				20,82	1,14	0,01				20,82	1,15	0,00
				21,43	1,13	0,01				21,43	1,14	0,00
				22,04	1,13	0,01				22,04	1,14	0,00
				22,65	1,12	0,01				22,65	1,14	0,00
				23,27	1,11	0,01				23,27	1,14	0,00
				23,88	1,11	0,01				23,88	1,13	0,00
				24,49	1,10	0,01				24,49	1,13	0,00
				25,10	1,09	0,01				25,10	1,13	0,00
				25,71	1,08	0,01				25,71	1,13	0,01
				26,33	1,07	0,01				26,33	1,12	0,01
				26,94	1,06	0,01				26,94	1,12	0,01
				27,55	1,05	0,02				27,55	1,11	0,01
				28,16	1,04	0,02				28,16	1,11	0,01
				28,78	1,03	0,02				28,78	1,10	0,01
				29,39	1,02	0,02				29,39	1,10	0,01
				30,00	1,01	0,03				30,00	1,09	0,01

© 2024 Chourpiliadis C et al. *JAMA Network Open.*

Depression, Anxiety or Stress-related disorders												
Log2 ApoB/ApoA-I ratio												
	Cases						Controls					
	Observed			Predicted			Observed			Predicted		
Time since index date	Number	Mean	SD	Time since index date	Mean	SE	Number	Mean	SD	Time since index date	Mean	SE
0	78	-0,22	0,24	0,00	-0,23	0,02	1014	-0,37	0,45	0,00	-0,33	0,02
1	110	-0,26	0,34	0,61	-0,26	0,02	1106	-0,30	0,47	0,61	-0,33	0,01
2	92	-0,31	0,37	1,22	-0,28	0,01	1411	-0,33	0,50	1,22	-0,33	0,01
3	168	-0,38	0,49	1,84	-0,30	0,01	1644	-0,31	0,53	1,84	-0,33	0,01
4	138	-0,32	0,53	2,45	-0,32	0,01	2002	-0,35	0,46	2,45	-0,33	0,01
5	333	-0,36	0,43	3,06	-0,33	0,01	2773	-0,36	0,48	3,06	-0,33	0,01
6	352	-0,34	0,48	3,67	-0,34	0,01	3829	-0,33	0,48	3,67	-0,34	0,01
7	456	-0,33	0,46	4,29	-0,35	0,01	4411	-0,32	0,51	4,29	-0,34	0,01
8	403	-0,38	0,48	4,90	-0,35	0,02	4853	-0,36	0,49	4,90	-0,34	0,01
9	526	-0,43	0,58	5,51	-0,36	0,02	5992	-0,37	0,48	5,51	-0,34	0,01
10	527	-0,29	0,49	6,12	-0,36	0,02	6812	-0,40	0,49	6,12	-0,34	0,01
11	506	-0,28	0,50	6,73	-0,36	0,02	7161	-0,36	0,49	6,73	-0,35	0,01
12	562	-0,35	0,45	7,35	-0,36	0,02	7744	-0,35	0,48	7,35	-0,35	0,01
13	694	-0,31	0,48	7,96	-0,35	0,02	8228	-0,35	0,48	7,96	-0,35	0,01
14	776	-0,26	0,49	8,57	-0,35	0,02	8681	-0,36	0,49	8,57	-0,35	0,01
15	792	-0,29	0,54	9,18	-0,35	0,02	8827	-0,35	0,49	9,18	-0,35	0,01
16	861	-0,28	0,53	9,80	-0,34	0,02	8899	-0,32	0,49	9,80	-0,35	0,01
17	927	-0,29	0,48	10,41	-0,34	0,02	8907	-0,33	0,49	10,41	-0,36	0,01
18	926	-0,25	0,51	11,02	-0,33	0,02	8973	-0,34	0,48	11,02	-0,36	0,01
19	966	-0,35	0,47	11,63	-0,33	0,01	8769	-0,33	0,48	11,63	-0,36	0,01
20	861	-0,31	0,53	12,24	-0,32	0,01	8745	-0,34	0,47	12,24	-0,36	0,00
21	894	-0,34	0,49	12,86	-0,32	0,01	8159	-0,31	0,48	12,86	-0,36	0,00
22	717	-0,29	0,46	13,47	-0,31	0,01	7954	-0,30	0,48	13,47	-0,35	0,00
23	789	-0,25	0,48	14,08	-0,31	0,01	7540	-0,30	0,47	14,08	-0,35	0,00
24	637	-0,31	0,48	14,69	-0,30	0,01	6319	-0,30	0,49	14,69	-0,35	0,00
25	577	-0,24	0,48	15,31	-0,30	0,01	5826	-0,27	0,47	15,31	-0,35	0,00
26	485	-0,35	0,43	15,92	-0,29	0,01	4676	-0,28	0,48	15,92	-0,35	0,00
27	389	-0,20	0,50	16,53	-0,29	0,01	3748	-0,26	0,46	16,53	-0,35	0,00
28	296	-0,33	0,45	17,14	-0,29	0,01	2676	-0,22	0,45	17,14	-0,34	0,00
29	190	-0,34	0,41	17,76	-0,29	0,01	1978	-0,21	0,44	17,76	-0,34	0,00
30	145	-0,35	0,37	18,37	-0,29	0,01	1485	-0,19	0,40	18,37	-0,34	0,00
				18,98	-0,28	0,01				18,98	-0,33	0,00
				19,59	-0,28	0,01				19,59	-0,33	0,00
				20,20	-0,28	0,01				20,20	-0,32	0,00
				20,82	-0,28	0,01				20,82	-0,32	0,00
				21,43	-0,28	0,01				21,43	-0,31	0,00
				22,04	-0,28	0,01				22,04	-0,31	0,00
				22,65	-0,29	0,02				22,65	-0,30	0,00
				23,27	-0,29	0,02				23,27	-0,30	0,00
				23,88	-0,29	0,02				23,88	-0,29	0,00
				24,49	-0,29	0,02				24,49	-0,28	0,00
				25,10	-0,29	0,02				25,10	-0,28	0,00
				25,71	-0,30	0,02				25,71	-0,27	0,00
				26,33	-0,30	0,03				26,33	-0,26	0,00
				26,94	-0,30	0,03				26,94	-0,25	0,00
				27,55	-0,31	0,03				27,55	-0,24	0,00
				28,16	-0,31	0,03				28,16	-0,23	0,00
				28,78	-0,32	0,03				28,78	-0,22	0,00
				29,39	-0,33	0,04				29,39	-0,20	0,01
				30,00	-0,33	0,06				30,00	-0,19	0,01

© 2024 Chourpiliadis C et al. *JAMA Network Open.*

Depression												
Glucose												
	Cases						Controls					
	Observed			Predicted			Observed			Predicted		
Time since index date	Number	Mean	SD	Time since index date	Mean	SE	Number	Mean	SD	Time since index date	Mean	SE
0	42	5,47	2,26	0,00	5,74	0,05	486	5,22	1,35	0,00	5,21	0,05
1	84	6,10	2,74	0,61	5,71	0,05	576	5,23	1,25	0,61	5,19	0,04
2	97	5,69	2,30	1,22	5,68	0,05	855	5,07	1,17	1,22	5,18	0,03
3	115	5,44	1,51	1,84	5,65	0,05	1028	5,14	1,36	1,84	5,16	0,02
4	140	5,43	1,50	2,45	5,61	0,05	1571	5,11	1,35	2,45	5,14	0,02
5	331	5,55	2,20	3,06	5,57	0,05	2366	5,06	1,22	3,06	5,13	0,02
6	348	5,32	1,68	3,67	5,54	0,05	3344	5,04	1,21	3,67	5,11	0,02
7	447	5,30	1,65	4,29	5,50	0,05	4017	5,07	1,26	4,29	5,10	0,02
8	481	5,30	1,79	4,90	5,46	0,05	4382	5,04	1,14	4,90	5,09	0,01
9	513	5,28	1,46	5,51	5,42	0,05	5123	5,05	1,24	5,51	5,07	0,01
10	605	5,16	1,52	6,12	5,38	0,04	5790	4,99	1,10	6,12	5,06	0,01
11	643	5,05	1,24	6,73	5,35	0,04	6600	4,97	1,03	6,73	5,05	0,01
12	746	5,10	1,61	7,35	5,31	0,03	7282	4,94	1,00	7,35	5,03	0,01
13	832	5,11	1,37	7,96	5,28	0,03	7930	4,93	0,90	7,96	5,02	0,01
14	873	5,03	1,33	8,57	5,24	0,02	8451	4,93	1,06	8,57	5,01	0,01
15	942	5,00	1,20	9,18	5,21	0,02	8903	4,90	1,06	9,18	5,00	0,01
16	948	5,04	1,60	9,80	5,18	0,02	9378	4,89	1,01	9,80	4,99	0,01
17	1001	4,95	1,11	10,41	5,16	0,02	9223	4,87	0,98	10,41	4,98	0,01
18	995	5,00	1,14	11,02	5,13	0,02	9513	4,88	0,99	11,02	4,97	0,01
19	1028	4,95	1,27	11,63	5,11	0,02	9507	4,86	0,93	11,63	4,96	0,00
20	976	4,95	1,19	12,24	5,09	0,02	9224	4,87	0,92	12,24	4,95	0,00
21	921	5,00	1,16	12,86	5,07	0,02	8899	4,87	0,93	12,86	4,94	0,00
22	842	5,00	1,21	13,47	5,06	0,01	8510	4,89	1,04	13,47	4,93	0,00
23	904	4,92	0,93	14,08	5,04	0,01	8051	4,86	0,91	14,08	4,92	0,00
24	745	4,93	1,17	14,69	5,03	0,01	7406	4,87	1,01	14,69	4,92	0,00
25	654	4,89	1,25	15,31	5,02	0,01	6645	4,83	0,92	15,31	4,91	0,00
26	557	4,78	0,78	15,92	5,01	0,01	5507	4,82	0,93	15,92	4,90	0,00
27	492	4,73	0,73	16,53	5,00	0,01	4708	4,77	0,81	16,53	4,90	0,00
28	428	4,82	0,76	17,14	4,99	0,01	3809	4,77	0,91	17,14	4,89	0,00
29	353	4,75	0,59	17,76	4,98	0,01	3071	4,77	0,83	17,76	4,89	0,00
30	250	4,68	0,66	18,37	4,97	0,01	2260	4,78	0,76	18,37	4,88	0,00
				18,98	4,97	0,01				18,98	4,88	0,00
				19,59	4,96	0,01				19,59	4,87	0,00
				20,20	4,96	0,01				20,20	4,87	0,00
				20,82	4,95	0,01				20,82	4,86	0,00
				21,43	4,94	0,01				21,43	4,86	0,00
				22,04	4,94	0,01				22,04	4,86	0,00
				22,65	4,93	0,01				22,65	4,85	0,00
				23,27	4,92	0,01				23,27	4,85	0,00
				23,88	4,91	0,01				23,88	4,84	0,00
				24,49	4,89	0,01				24,49	4,84	0,00
				25,10	4,88	0,02				25,10	4,83	0,00
				25,71	4,86	0,02				25,71	4,82	0,00
				26,33	4,84	0,02				26,33	4,82	0,00
				26,94	4,82	0,02				26,94	4,81	0,00
				27,55	4,80	0,02				27,55	4,80	0,00
				28,16	4,77	0,02				28,16	4,79	0,01
				28,78	4,74	0,02				28,78	4,78	0,01
				29,39	4,71	0,03				29,39	4,77	0,01
				30,00	4,67	0,04				30,00	4,75	0,01

© 2024 Chourpiliadis C et al. *JAMA Network Open.*

Depression												
Total Cholesterol												
	Cases						Controls					
	Observed			Predicted			Observed			Predicted		
Time since index date	Number	Mean	SD	Time since index date	Mean	SE	Number	Mean	SD	Time since index date	Mean	SE
0	49	6,32	1,01	0,00	6,18	0,05	509	5,92	1,05	0,00	5,92	0,01
1	81	5,99	1,09	0,61	6,14	0,04	622	5,87	1,01	0,61	5,90	0,01
2	104	5,99	1,03	1,22	6,10	0,03	914	5,84	1,08	1,22	5,87	0,01
3	118	5,93	1,07	1,84	6,07	0,03	1090	5,84	1,10	1,84	5,85	0,01
4	149	6,05	1,19	2,45	6,03	0,03	1673	5,77	1,06	2,45	5,82	0,01
5	343	5,90	1,14	3,06	6,00	0,03	2517	5,72	1,10	3,06	5,80	0,01
6	359	5,81	1,13	3,67	5,97	0,03	3565	5,70	1,12	3,67	5,78	0,01
7	467	5,83	1,27	4,29	5,94	0,03	4262	5,68	1,10	4,29	5,76	0,01
8	498	5,90	1,25	4,90	5,91	0,03	4605	5,67	1,12	4,90	5,74	0,01
9	542	5,75	1,19	5,51	5,88	0,03	5414	5,63	1,12	5,51	5,73	0,01
10	638	5,73	1,19	6,12	5,86	0,03	6112	5,67	1,13	6,12	5,71	0,01
11	683	5,69	1,10	6,73	5,83	0,03	7007	5,62	1,13	6,73	5,70	0,01
12	808	5,62	1,24	7,35	5,81	0,02	7701	5,61	1,13	7,35	5,68	0,01
13	888	5,65	1,13	7,96	5,78	0,02	8381	5,60	1,14	7,96	5,67	0,01
14	932	5,66	1,25	8,57	5,76	0,02	8917	5,59	1,14	8,57	5,66	0,01
15	999	5,59	1,15	9,18	5,74	0,02	9404	5,59	1,13	9,18	5,65	0,01
16	992	5,63	1,20	9,80	5,72	0,02	9873	5,58	1,17	9,80	5,64	0,00
17	1051	5,53	1,14	10,41	5,70	0,02	9775	5,57	1,14	10,41	5,63	0,00
18	1044	5,57	1,11	11,02	5,69	0,01	10024	5,55	1,13	11,02	5,62	0,00
19	1076	5,53	1,11	11,63	5,67	0,01	9977	5,57	1,16	11,63	5,61	0,00
20	1025	5,61	1,13	12,24	5,66	0,01	9765	5,59	1,14	12,24	5,60	0,00
21	975	5,59	1,07	12,86	5,64	0,01	9391	5,59	1,16	12,86	5,60	0,00
22	883	5,65	1,12	13,47	5,63	0,01	8945	5,60	1,13	13,47	5,59	0,00
23	933	5,59	1,18	14,08	5,62	0,01	8468	5,59	1,14	14,08	5,59	0,00
24	784	5,64	1,14	14,69	5,61	0,01	7795	5,61	1,13	14,69	5,59	0,00
25	682	5,58	1,11	15,31	5,61	0,01	6958	5,57	1,12	15,31	5,58	0,00
26	584	5,54	1,07	15,92	5,60	0,01	5761	5,56	1,12	15,92	5,58	0,00
27	503	5,60	1,15	16,53	5,59	0,01	4903	5,58	1,13	16,53	5,58	0,00
28	442	5,67	1,14	17,14	5,59	0,01	3972	5,57	1,12	17,14	5,58	0,00
29	370	5,56	1,07	17,76	5,59	0,01	3207	5,56	1,10	17,76	5,58	0,00
30	259	5,45	1,05	18,37	5,59	0,01	2363	5,55	1,14	18,37	5,58	0,00
				18,98	5,59	0,01				18,98	5,58	0,00
				19,59	5,59	0,01				19,59	5,58	0,00
				20,20	5,59	0,01				20,20	5,58	0,00
				20,82	5,59	0,01				20,82	5,58	0,00
				21,43	5,60	0,01				21,43	5,58	0,00
				22,04	5,60	0,01				22,04	5,58	0,00
				22,65	5,60	0,01				22,65	5,58	0,00
				23,27	5,61	0,01				23,27	5,58	0,01
				23,88	5,61	0,01				23,88	5,58	0,01
				24,49	5,61	0,02				24,49	5,58	0,01
				25,10	5,61	0,02				25,10	5,58	0,01
				25,71	5,61	0,02				25,71	5,58	0,01
				26,33	5,60	0,02				26,33	5,58	0,01
				26,94	5,59	0,02				26,94	5,58	0,01
				27,55	5,58	0,02				27,55	5,57	0,01
				28,16	5,57	0,02				28,16	5,57	0,01
				28,78	5,55	0,02				28,78	5,56	0,01
				29,39	5,53	0,03				29,39	5,56	0,01
				30,00	5,50	0,04				30,00	5,55	0,01

© 2024 Chourpiliadis C et al. *JAMA Network Open.*

Depression												
Log2 Triglycerides												
	Cases						Controls					
	Observed			Predicted			Observed			Predicted		
Time since index date	Number	Mean	SD	Time since index date	Mean	SE	Number	Mean	SD	Time since index date	Mean	SE
0	48	0,48	1,04	0,00	0,49	0,03	508	0,29	0,81	0,00	0,30	0,01
1	81	0,51	1,01	0,61	0,46	0,02	621	0,28	0,85	0,61	0,28	0,01
2	104	0,38	0,94	1,22	0,43	0,02	912	0,24	0,80	1,22	0,27	0,01
3	117	0,36	1,00	1,84	0,41	0,02	1084	0,24	0,81	1,84	0,25	0,01
4	148	0,25	0,83	2,45	0,38	0,01	1666	0,20	0,79	2,45	0,24	0,01
5	342	0,27	0,86	3,06	0,36	0,01	2510	0,16	0,81	3,06	0,22	0,01
6	358	0,30	0,83	3,67	0,34	0,01	3548	0,17	0,81	3,67	0,21	0,01
7	466	0,29	0,83	4,29	0,33	0,01	4239	0,15	0,81	4,29	0,20	0,01
8	497	0,29	0,83	4,90	0,31	0,01	4591	0,15	0,80	4,90	0,19	0,01
9	539	0,22	0,81	5,51	0,30	0,01	5387	0,14	0,81	5,51	0,18	0,01
10	637	0,30	0,84	6,12	0,29	0,01	6088	0,15	0,82	6,12	0,17	0,00
11	678	0,30	0,86	6,73	0,28	0,02	6970	0,13	0,81	6,73	0,16	0,00
12	791	0,18	0,86	7,35	0,27	0,02	7664	0,12	0,81	7,35	0,16	0,00
13	875	0,23	0,87	7,96	0,26	0,01	8331	0,12	0,81	7,96	0,15	0,00
14	924	0,22	0,85	8,57	0,25	0,01	8885	0,10	0,82	8,57	0,14	0,00
15	990	0,12	0,78	9,18	0,24	0,01	9378	0,10	0,80	9,18	0,14	0,00
16	986	0,17	0,83	9,80	0,23	0,01	9822	0,10	0,82	9,80	0,13	0,00
17	1045	0,15	0,81	10,41	0,22	0,01	9714	0,08	0,82	10,41	0,13	0,00
18	1041	0,11	0,80	11,02	0,22	0,01	9966	0,09	0,81	11,02	0,12	0,00
19	1072	0,11	0,82	11,63	0,21	0,01	9925	0,08	0,80	11,63	0,12	0,00
20	1022	0,19	0,85	12,24	0,21	0,01	9717	0,09	0,81	12,24	0,12	0,00
21	973	0,15	0,81	12,86	0,20	0,01	9335	0,07	0,79	12,86	0,11	0,00
22	880	0,16	0,83	13,47	0,19	0,01	8898	0,09	0,81	13,47	0,11	0,00
23	930	0,14	0,83	14,08	0,19	0,01	8421	0,09	0,80	14,08	0,11	0,00
24	782	0,16	0,81	14,69	0,19	0,01	7763	0,08	0,80	14,69	0,11	0,00
25	680	0,16	0,88	15,31	0,18	0,01	6933	0,06	0,79	15,31	0,10	0,00
26	581	0,10	0,78	15,92	0,18	0,01	5739	0,06	0,80	15,92	0,10	0,00
27	503	0,05	0,84	16,53	0,17	0,01	4885	0,04	0,80	16,53	0,10	0,00
28	441	0,12	0,86	17,14	0,17	0,01	3957	0,02	0,79	17,14	0,10	0,00
29	370	0,03	0,76	17,76	0,17	0,01	3198	0,04	0,80	17,76	0,09	0,00
30	259	-0,02	0,76	18,37	0,16	0,01	2359	0,05	0,80	18,37	0,09	0,00
				18,98	0,16	0,01				18,98	0,09	0,00
				19,59	0,16	0,01				19,59	0,09	0,00
				20,20	0,16	0,01				20,20	0,08	0,00
				20,82	0,16	0,01				20,82	0,08	0,00
				21,43	0,15	0,01				21,43	0,08	0,00
				22,04	0,15	0,01				22,04	0,08	0,00
				22,65	0,15	0,01				22,65	0,07	0,00
				23,27	0,14	0,01				23,27	0,07	0,00
				23,88	0,14	0,01				23,88	0,07	0,00
				24,49	0,13	0,01				24,49	0,06	0,00
				25,10	0,13	0,01				25,10	0,06	0,00
				25,71	0,12	0,01				25,71	0,06	0,00
				26,33	0,11	0,01				26,33	0,05	0,00
				26,94	0,10	0,01				26,94	0,05	0,00
				27,55	0,08	0,02				27,55	0,05	0,00
				28,16	0,07	0,02				28,16	0,04	0,00
				28,78	0,05	0,02				28,78	0,04	0,00
				29,39	0,02	0,03				29,39	0,04	0,01
				30,00	0,00	0,04				30,00	0,03	0,01

© 2024 Chourpiliadis C et al. *JAMA Network Open.*

Depression												
LDL-C												
	Cases						Controls					
	Observed			Predicted			Observed			Predicted		
Time since index date	Number	Mean	SD	Time since index date	Mean	SE	Number	Mean	SD	Time since index date	Mean	SE
0	26	4,06	1,20	0,00	3,85	0,02	232	3,91	1,00	0,00	3,88	0,03
1	43	3,56	1,14	0,61	3,84	0,02	291	3,78	0,94	0,61	3,86	0,02
2	46	3,72	1,04	1,22	3,84	0,02	371	3,82	0,95	1,22	3,84	0,02
3	57	3,89	1,16	1,84	3,83	0,02	447	3,83	1,10	1,84	3,82	0,02
4	60	3,96	1,16	2,45	3,83	0,02	617	3,77	0,99	2,45	3,80	0,02
5	128	3,87	1,08	3,06	3,82	0,02	976	3,69	1,04	3,06	3,79	0,02
6	134	3,68	1,09	3,67	3,81	0,03	1325	3,72	1,04	3,67	3,77	0,01
7	182	3,72	1,11	4,29	3,80	0,03	1614	3,68	1,05	4,29	3,75	0,01
8	176	3,79	1,18	4,90	3,80	0,03	1746	3,70	1,05	4,90	3,74	0,01
9	206	3,69	1,07	5,51	3,79	0,04	1985	3,65	1,03	5,51	3,72	0,01
10	234	3,76	1,14	6,12	3,78	0,04	2311	3,68	1,07	6,12	3,71	0,01
11	250	3,63	1,02	6,73	3,77	0,04	2645	3,60	1,04	6,73	3,70	0,01
12	304	3,69	1,12	7,35	3,76	0,03	2794	3,65	1,08	7,35	3,69	0,01
13	328	3,75	1,06	7,96	3,75	0,03	2975	3,63	1,06	7,96	3,68	0,01
14	295	3,74	1,23	8,57	3,74	0,03	3013	3,61	1,08	8,57	3,67	0,01
15	341	3,63	1,12	9,18	3,73	0,02	3274	3,60	1,06	9,18	3,66	0,01
16	321	3,68	1,12	9,80	3,72	0,02	3380	3,61	1,09	9,80	3,65	0,01
17	324	3,61	1,07	10,41	3,71	0,02	3271	3,64	1,10	10,41	3,64	0,01
18	344	3,62	1,12	11,02	3,70	0,02	3248	3,57	1,09	11,02	3,64	0,01
19	338	3,53	1,05	11,63	3,69	0,02	3194	3,59	1,08	11,63	3,63	0,01
20	327	3,64	1,09	12,24	3,68	0,02	3125	3,61	1,08	12,24	3,63	0,01
21	309	3,50	1,10	12,86	3,67	0,02	3027	3,68	1,13	12,86	3,62	0,01
22	272	3,62	1,03	13,47	3,66	0,02	2858	3,65	1,09	13,47	3,62	0,01
23	301	3,56	1,04	14,08	3,66	0,02	2641	3,61	1,05	14,08	3,62	0,01
24	248	3,62	1,00	14,69	3,65	0,02	2419	3,65	1,06	14,69	3,61	0,01
25	186	3,57	1,18	15,31	3,64	0,02	2036	3,62	1,08	15,31	3,61	0,01
26	166	3,50	1,04	15,92	3,64	0,02	1585	3,57	1,09	15,92	3,61	0,01
27	145	3,60	1,12	16,53	3,63	0,02	1307	3,59	1,07	16,53	3,61	0,01
28	116	3,49	1,07	17,14	3,63	0,02	1084	3,59	1,05	17,14	3,61	0,01
29	101	3,59	1,14	17,76	3,62	0,02	835	3,57	1,00	17,76	3,61	0,01
30	54	3,14	0,96	18,37	3,62	0,02	515	3,54	1,08	18,37	3,62	0,01
				18,98	3,62	0,02				18,98	3,62	0,01
				19,59	3,62	0,02				19,59	3,62	0,01
				20,20	3,61	0,02				20,20	3,62	0,01
				20,82	3,61	0,02				20,82	3,62	0,01
				21,43	3,61	0,02				21,43	3,62	0,01
				22,04	3,61	0,02				22,04	3,62	0,01
				22,65	3,60	0,02				22,65	3,62	0,01
				23,27	3,60	0,02				23,27	3,62	0,01
				23,88	3,59	0,02				23,88	3,62	0,01
				24,49	3,58	0,03				24,49	3,62	0,01
				25,10	3,57	0,03				25,10	3,62	0,01
				25,71	3,56	0,03				25,71	3,61	0,01
				26,33	3,54	0,03				26,33	3,61	0,01
				26,94	3,52	0,03				26,94	3,60	0,01
				27,55	3,49	0,03				27,55	3,59	0,01
				28,16	3,46	0,03				28,16	3,58	0,01
				28,78	3,42	0,04				28,78	3,57	0,01
				29,39	3,37	0,05				29,39	3,55	0,01
				30,00	3,31	0,07				30,00	3,54	0,01

© 2024 Chourpiliadis C et al. *JAMA Network Open.*

Depression												
HDL-C												
	Cases						Controls					
	Observed			Predicted			Observed			Predicted		
Time since index date	Number	Mean	SD	Time since index date	Mean	SE	Number	Mean	SD	Time since index date	Mean	SE
0	27	1,48	0,32	0,00	1,48	0,04	233	1,57	0,39	0,00	1,56	0,01
1	42	1,52	0,36	0,61	1,51	0,03	293	1,55	0,41	0,61	1,56	0,01
2	45	1,52	0,36	1,22	1,52	0,03	354	1,56	0,39	1,22	1,56	0,01
3	53	1,55	0,44	1,84	1,54	0,02	451	1,56	0,41	1,84	1,56	0,00
4	59	1,68	0,55	2,45	1,55	0,02	620	1,55	0,39	2,45	1,56	0,00
5	125	1,59	0,45	3,06	1,56	0,02	970	1,55	0,39	3,06	1,56	0,00
6	129	1,51	0,39	3,67	1,56	0,02	1342	1,56	0,39	3,67	1,56	0,00
7	191	1,53	0,41	4,29	1,57	0,02	1616	1,59	0,40	4,29	1,56	0,00
8	180	1,53	0,37	4,90	1,57	0,02	1796	1,56	0,38	4,90	1,56	0,00
9	204	1,62	0,44	5,51	1,57	0,02	2040	1,57	0,40	5,51	1,56	0,00
10	223	1,54	0,40	6,12	1,57	0,02	2359	1,57	0,39	6,12	1,56	0,00
11	244	1,54	0,38	6,73	1,57	0,02	2704	1,58	0,40	6,73	1,56	0,00
12	312	1,54	0,37	7,35	1,57	0,02	2887	1,57	0,39	7,35	1,57	0,00
13	326	1,53	0,39	7,96	1,57	0,02	3049	1,56	0,40	7,96	1,57	0,00
14	296	1,55	0,38	8,57	1,56	0,02	3068	1,56	0,39	8,57	1,57	0,00
15	347	1,57	0,41	9,18	1,56	0,01	3316	1,57	0,39	9,18	1,57	0,00
16	333	1,55	0,40	9,80	1,56	0,01	3404	1,58	0,39	9,80	1,57	0,00
17	334	1,51	0,35	10,41	1,55	0,01	3319	1,57	0,39	10,41	1,57	0,00
18	338	1,58	0,38	11,02	1,55	0,01	3296	1,58	0,39	11,02	1,57	0,00
19	334	1,61	0,38	11,63	1,55	0,01	3226	1,57	0,39	11,63	1,57	0,00
20	326	1,54	0,41	12,24	1,55	0,01	3157	1,57	0,38	12,24	1,57	0,00
21	310	1,60	0,41	12,86	1,54	0,01	3047	1,58	0,40	12,86	1,57	0,00
22	261	1,59	0,37	13,47	1,54	0,01	2853	1,58	0,39	13,47	1,57	0,00
23	298	1,60	0,42	14,08	1,54	0,01	2630	1,58	0,38	14,08	1,57	0,00
24	245	1,58	0,39	14,69	1,54	0,01	2396	1,58	0,38	14,69	1,57	0,00
25	190	1,61	0,41	15,31	1,54	0,01	2048	1,58	0,37	15,31	1,57	0,00
26	162	1,62	0,34	15,92	1,54	0,01	1562	1,60	0,38	15,92	1,57	0,00
27	136	1,65	0,38	16,53	1,55	0,01	1265	1,59	0,37	16,53	1,57	0,00
28	111	1,64	0,39	17,14	1,55	0,01	1039	1,60	0,37	17,14	1,57	0,00
29	95	1,66	0,39	17,76	1,55	0,01	805	1,58	0,37	17,76	1,58	0,00
30	51	1,61	0,33	18,37	1,56	0,01	489	1,58	0,37	18,37	1,58	0,00
				18,98	1,56	0,01				18,98	1,58	0,00
				19,59	1,57	0,01				19,59	1,58	0,00
				20,20	1,57	0,01				20,20	1,58	0,00
				20,82	1,58	0,01				20,82	1,58	0,00
				21,43	1,58	0,01				21,43	1,58	0,00
				22,04	1,59	0,01				22,04	1,58	0,00
				22,65	1,60	0,01				22,65	1,58	0,00
				23,27	1,60	0,01				23,27	1,58	0,00
				23,88	1,61	0,01				23,88	1,58	0,00
				24,49	1,62	0,01				24,49	1,58	0,00
				25,10	1,62	0,01				25,10	1,59	0,00
				25,71	1,63	0,01				25,71	1,59	0,00
				26,33	1,63	0,01				26,33	1,59	0,00
				26,94	1,63	0,01				26,94	1,59	0,00
				27,55	1,64	0,01				27,55	1,59	0,00
				28,16	1,64	0,01				28,16	1,59	0,00
				28,78	1,64	0,01				28,78	1,58	0,00
				29,39	1,64	0,01				29,39	1,58	0,00
				30,00	1,63	0,01				30,00	1,58	0,00

© 2024 Chourpiliadis C et al. *JAMA Network Open.*

Depression												
ApoA-I												
	Cases						Controls					
	Observed			Predicted			Observed			Predicted		
Time since index date	Number	Mean	SD	Time since index date	Mean	SE	Number	Mean	SD	Time since index date	Mean	SE
0	24	1,51	0,26	0,00	1,52	0,02	204	1,44	0,23	0,00	1,44	0,01
1	37	1,52	0,25	0,61	1,51	0,01	258	1,43	0,23	0,61	1,43	0,01
2	43	1,49	0,21	1,22	1,50	0,01	331	1,43	0,23	1,22	1,43	0,01
3	54	1,46	0,24	1,84	1,49	0,01	399	1,45	0,23	1,84	1,43	0,00
4	53	1,50	0,30	2,45	1,48	0,01	556	1,43	0,22	2,45	1,43	0,00
5	115	1,45	0,29	3,06	1,48	0,01	810	1,42	0,23	3,06	1,43	0,00
6	115	1,42	0,24	3,67	1,47	0,01	1080	1,42	0,23	3,67	1,43	0,00
7	148	1,42	0,21	4,29	1,46	0,01	1291	1,44	0,23	4,29	1,43	0,00
8	138	1,43	0,20	4,90	1,46	0,01	1464	1,42	0,21	4,90	1,43	0,00
9	160	1,46	0,24	5,51	1,45	0,01	1646	1,43	0,23	5,51	1,43	0,00
10	194	1,42	0,24	6,12	1,45	0,01	1932	1,43	0,22	6,12	1,43	0,00
11	189	1,44	0,23	6,73	1,44	0,01	2182	1,43	0,23	6,73	1,43	0,00
12	255	1,40	0,22	7,35	1,44	0,01	2375	1,42	0,22	7,35	1,43	0,00
13	264	1,42	0,21	7,96	1,43	0,01	2565	1,42	0,22	7,96	1,43	0,00
14	261	1,44	0,23	8,57	1,43	0,01	2649	1,41	0,22	8,57	1,43	0,00
15	296	1,42	0,23	9,18	1,43	0,01	2884	1,42	0,22	9,18	1,43	0,00
16	292	1,42	0,22	9,80	1,42	0,01	2971	1,42	0,23	9,80	1,43	0,00
17	289	1,40	0,20	10,41	1,42	0,01	2911	1,42	0,22	10,41	1,43	0,00
18	293	1,41	0,21	11,02	1,42	0,01	2869	1,43	0,22	11,02	1,42	0,00
19	310	1,44	0,23	11,63	1,42	0,01	2850	1,42	0,22	11,63	1,42	0,00
20	305	1,41	0,24	12,24	1,42	0,01	2841	1,42	0,22	12,24	1,42	0,00
21	275	1,46	0,25	12,86	1,42	0,01	2741	1,42	0,23	12,86	1,42	0,00
22	247	1,43	0,22	13,47	1,42	0,01	2593	1,42	0,22	13,47	1,42	0,00
23	271	1,43	0,24	14,08	1,42	0,01	2401	1,42	0,22	14,08	1,42	0,00
24	228	1,44	0,24	14,69	1,42	0,01	2229	1,42	0,22	14,69	1,42	0,00
25	186	1,44	0,22	15,31	1,42	0,01	1946	1,42	0,21	15,31	1,42	0,00
26	166	1,44	0,22	15,92	1,42	0,01	1558	1,42	0,22	15,92	1,42	0,00
27	145	1,45	0,23	16,53	1,42	0,01	1307	1,42	0,22	16,53	1,42	0,00
28	116	1,45	0,23	17,14	1,42	0,01	1082	1,42	0,22	17,14	1,42	0,00
29	101	1,44	0,23	17,76	1,42	0,01	836	1,41	0,21	17,76	1,42	0,00
30	54	1,42	0,24	18,37	1,42	0,01	515	1,41	0,21	18,37	1,42	0,00
				18,98	1,43	0,01				18,98	1,42	0,00
				19,59	1,43	0,01				19,59	1,42	0,00
				20,20	1,43	0,01				20,20	1,42	0,00
				20,82	1,43	0,01				20,82	1,42	0,00
				21,43	1,43	0,01				21,43	1,42	0,00
				22,04	1,44	0,01				22,04	1,42	0,00
				22,65	1,44	0,00				22,65	1,42	0,00
				23,27	1,44	0,00				23,27	1,42	0,00
				23,88	1,44	0,00				23,88	1,42	0,00
				24,49	1,44	0,00				24,49	1,42	0,00
				25,10	1,44	0,00				25,10	1,42	0,00
				25,71	1,44	0,00				25,71	1,42	0,00
				26,33	1,44	0,00				26,33	1,42	0,00
				26,94	1,44	0,00				26,94	1,42	0,00
				27,55	1,44	0,00				27,55	1,42	0,00
				28,16	1,44	0,00				28,16	1,42	0,00
				28,78	1,44	0,00				28,78	1,42	0,00
				29,39	1,43	0,00				29,39	1,41	0,00
				30,00	1,43	0,00				30,00	1,41	0,00

© 2024 Chourpiliadis C et al. *JAMA Network Open.*

Depression												
ApoB												
	Cases						Controls					
	Observed			Predicted			Observed			Predicted		
Time since index date	Number	Mean	SD	Time since index date	Mean	SE	Number	Mean	SD	Time since index date	Mean	SE
0	26	1,47	0,37	0,00	1,46	0,02	216	1,24	0,35	0,00	1,25	0,01
1	37	1,35	0,43	0,61	1,43	0,01	258	1,24	0,35	0,61	1,25	0,01
2	40	1,40	0,37	1,22	1,41	0,01	334	1,26	0,32	1,22	1,24	0,01
3	51	1,40	0,42	1,84	1,38	0,01	393	1,23	0,35	1,84	1,24	0,00
4	46	1,35	0,38	2,45	1,36	0,01	552	1,23	0,34	2,45	1,23	0,00
5	113	1,28	0,39	3,06	1,34	0,01	756	1,20	0,34	3,06	1,23	0,00
6	99	1,18	0,37	3,67	1,32	0,01	1035	1,21	0,35	3,67	1,23	0,00
7	125	1,23	0,39	4,29	1,31	0,02	1217	1,20	0,36	4,29	1,22	0,00
8	123	1,23	0,41	4,90	1,29	0,02	1401	1,20	0,36	4,90	1,22	0,00
9	162	1,24	0,36	5,51	1,28	0,02	1631	1,19	0,35	5,51	1,21	0,00
10	184	1,25	0,39	6,12	1,27	0,02	1870	1,20	0,36	6,12	1,21	0,00
11	182	1,26	0,40	6,73	1,26	0,01	2036	1,19	0,37	6,73	1,21	0,00
12	232	1,19	0,38	7,35	1,25	0,01	2230	1,20	0,36	7,35	1,20	0,00
13	235	1,24	0,36	7,96	1,24	0,01	2423	1,19	0,37	7,96	1,20	0,00
14	255	1,24	0,42	8,57	1,24	0,01	2514	1,19	0,37	8,57	1,20	0,00
15	257	1,23	0,37	9,18	1,23	0,01	2584	1,20	0,35	9,18	1,20	0,00
16	254	1,22	0,39	9,80	1,22	0,01	2631	1,20	0,36	9,80	1,19	0,00
17	261	1,18	0,33	10,41	1,22	0,01	2613	1,20	0,36	10,41	1,19	0,00
18	266	1,19	0,38	11,02	1,22	0,01	2612	1,19	0,36	11,02	1,19	0,00
19	277	1,20	0,36	11,63	1,21	0,01	2507	1,20	0,36	11,63	1,19	0,00
20	287	1,22	0,34	12,24	1,21	0,01	2538	1,21	0,35	12,24	1,19	0,00
21	253	1,19	0,35	12,86	1,21	0,01	2391	1,22	0,37	12,86	1,19	0,00
22	211	1,23	0,39	13,47	1,21	0,01	2337	1,23	0,36	13,47	1,19	0,00
23	230	1,23	0,34	14,08	1,21	0,01	2095	1,22	0,34	14,08	1,19	0,00
24	200	1,29	0,38	14,69	1,21	0,01	1972	1,24	0,35	14,69	1,19	0,00
25	168	1,23	0,37	15,31	1,21	0,01	1674	1,25	0,34	15,31	1,19	0,00
26	130	1,23	0,34	15,92	1,21	0,01	1333	1,25	0,34	15,92	1,19	0,00
27	123	1,30	0,37	16,53	1,21	0,01	1037	1,28	0,36	16,53	1,20	0,00
28	79	1,23	0,32	17,14	1,21	0,01	786	1,27	0,34	17,14	1,20	0,00
29	65	1,22	0,30	17,76	1,22	0,01	631	1,27	0,31	17,76	1,20	0,00
30	41	1,16	0,28	18,37	1,22	0,01	419	1,26	0,31	18,37	1,20	0,00
				18,98	1,22	0,01				18,98	1,21	0,00
				19,59	1,22	0,01				19,59	1,21	0,00
				20,20	1,23	0,01				20,20	1,21	0,00
				20,82	1,23	0,01				20,82	1,22	0,00
				21,43	1,23	0,01				21,43	1,22	0,00
				22,04	1,24	0,01				22,04	1,23	0,00
				22,65	1,24	0,01				22,65	1,23	0,00
				23,27	1,24	0,01				23,27	1,24	0,00
				23,88	1,24	0,01				23,88	1,24	0,00
				24,49	1,24	0,01				24,49	1,24	0,00
				25,10	1,24	0,01				25,10	1,25	0,00
				25,71	1,24	0,01				25,71	1,25	0,00
				26,33	1,24	0,01				26,33	1,26	0,00
				26,94	1,24	0,01				26,94	1,26	0,00
				27,55	1,23	0,01				27,55	1,26	0,00
				28,16	1,23	0,02				28,16	1,27	0,00
				28,78	1,22	0,02				28,78	1,27	0,00
				29,39	1,21	0,02				29,39	1,27	0,01
				30,00	1,19	0,03				30,00	1,27	0,01

© 2024 Chourpiliadis C et al. *JAMA Network Open.*

Depression												
Log2 LDL-C/ HDL-C ratio												
	Cases						Controls					
	Observed			Predicted			Observed			Predicted		
Time since index date	Number	Mean	SD	Time since index date	Mean	SE	Number	Mean	SD	Time since index date	Mean	SE
0	26	1,42	0,57	0,00	1,34	0,03	219	1,31	0,58	0,00	1,31	0,01
1	40	1,22	0,62	0,61	1,33	0,03	274	1,28	0,59	0,61	1,30	0,01
2	42	1,27	0,56	1,22	1,31	0,02	329	1,31	0,55	1,22	1,29	0,01
3	50	1,34	0,66	1,84	1,30	0,02	406	1,27	0,60	1,84	1,28	0,01
4	58	1,24	0,70	2,45	1,29	0,02	570	1,26	0,58	2,45	1,28	0,01
5	113	1,25	0,63	3,06	1,28	0,02	909	1,22	0,58	3,06	1,27	0,01
6	121	1,24	0,54	3,67	1,27	0,02	1259	1,23	0,59	3,67	1,26	0,01
7	176	1,25	0,64	4,29	1,26	0,02	1516	1,21	0,59	4,29	1,25	0,01
8	161	1,27	0,62	4,90	1,26	0,02	1639	1,22	0,60	4,90	1,24	0,01
9	194	1,17	0,62	5,51	1,25	0,02	1898	1,20	0,60	5,51	1,24	0,01
10	212	1,26	0,62	6,12	1,25	0,02	2206	1,20	0,60	6,12	1,23	0,01
11	230	1,22	0,58	6,73	1,24	0,02	2540	1,16	0,61	6,73	1,22	0,01
12	292	1,24	0,61	7,35	1,24	0,01	2688	1,19	0,61	7,35	1,22	0,00
13	308	1,28	0,59	7,96	1,24	0,01	2846	1,19	0,61	7,96	1,21	0,00
14	283	1,22	0,61	8,57	1,23	0,01	2850	1,18	0,61	8,57	1,20	0,00
15	327	1,18	0,66	9,18	1,23	0,01	3120	1,16	0,61	9,18	1,20	0,00
16	309	1,21	0,62	9,80	1,23	0,01	3203	1,17	0,62	9,80	1,19	0,00
17	310	1,21	0,60	10,41	1,23	0,01	3131	1,17	0,61	10,41	1,19	0,00
18	325	1,14	0,67	11,02	1,22	0,01	3099	1,14	0,62	11,02	1,19	0,00
19	321	1,09	0,59	11,63	1,22	0,01	3044	1,16	0,61	11,63	1,18	0,00
20	307	1,21	0,63	12,24	1,22	0,01	2970	1,16	0,60	12,24	1,18	0,00
21	294	1,09	0,66	12,86	1,21	0,01	2903	1,19	0,62	12,86	1,18	0,00
22	255	1,16	0,57	13,47	1,21	0,01	2739	1,17	0,60	13,47	1,17	0,00
23	289	1,13	0,62	14,08	1,21	0,01	2535	1,16	0,60	14,08	1,17	0,00
24	237	1,19	0,61	14,69	1,20	0,01	2331	1,17	0,59	14,69	1,17	0,00
25	179	1,11	0,60	15,31	1,20	0,01	1963	1,16	0,60	15,31	1,17	0,00
26	158	1,08	0,56	15,92	1,20	0,01	1519	1,12	0,62	15,92	1,17	0,00
27	134	1,07	0,63	16,53	1,19	0,01	1246	1,14	0,58	16,53	1,17	0,00
28	111	1,04	0,61	17,14	1,19	0,01	1039	1,14	0,58	17,14	1,17	0,00
29	95	1,06	0,66	17,76	1,18	0,02	804	1,15	0,58	17,76	1,17	0,00
30	51	0,90	0,68	18,37	1,18	0,02	488	1,13	0,62	18,37	1,16	0,00
				18,98	1,18	0,02				18,98	1,16	0,00
				19,59	1,17	0,02				19,59	1,16	0,00
				20,20	1,17	0,02				20,20	1,16	0,00
				20,82	1,16	0,02				20,82	1,16	0,00
				21,43	1,15	0,02				21,43	1,16	0,00
				22,04	1,15	0,02				22,04	1,16	0,00
				22,65	1,14	0,02				22,65	1,16	0,00
				23,27	1,13	0,02				23,27	1,16	0,00
				23,88	1,13	0,02				23,88	1,16	0,00
				24,49	1,12	0,02				24,49	1,16	0,00
				25,10	1,11	0,02				25,10	1,16	0,00
				25,71	1,10	0,01				25,71	1,15	0,00
				26,33	1,08	0,01				26,33	1,15	0,00
				26,94	1,07	0,01				26,94	1,15	0,00
				27,55	1,05	0,01				27,55	1,14	0,00
				28,16	1,03	0,01				28,16	1,14	0,00
				28,78	1,01	0,01				28,78	1,14	0,00
				29,39	0,98	0,01				29,39	1,13	0,01
				30,00	0,95	0,02				30,00	1,13	0,01

© 2024 Chourpiliadis C et al. *JAMA Network Open.*

Depression												
Log2 ApoB/ApoA-I ratio												
	Cases						Controls					
	Observed			Predicted			Observed			Predicted		
Time since index date	Number	Mean	SD	Time since index date	Mean	SE	Number	Mean	SD	Time since index date	Mean	SE
0	74	-0,21	0,23	0,00	-0,21	0,02	692	-0,24	0,49	0,00	-0,22	0,02
1	97	-0,25	0,36	0,61	-0,23	0,01	716	-0,22	0,42	0,61	-0,23	0,01
2	64	-0,26	0,31	1,22	-0,25	0,01	869	-0,26	0,42	1,22	-0,24	0,01
3	105	-0,31	0,47	1,84	-0,27	0,01	1060	-0,26	0,43	1,84	-0,25	0,01
4	99	-0,26	0,54	2,45	-0,28	0,01	1226	-0,25	0,46	2,45	-0,26	0,01
5	182	-0,29	0,45	3,06	-0,30	0,01	1794	-0,30	0,44	3,06	-0,27	0,01
6	229	-0,33	0,45	3,67	-0,31	0,01	2177	-0,32	0,47	3,67	-0,28	0,01
7	276	-0,33	0,50	4,29	-0,32	0,02	2629	-0,34	0,51	4,29	-0,29	0,01
8	276	-0,42	0,50	4,90	-0,33	0,02	3016	-0,34	0,52	4,90	-0,30	0,01
9	319	-0,50	0,56	5,51	-0,33	0,02	3462	-0,32	0,49	5,51	-0,30	0,01
10	371	-0,27	0,53	6,12	-0,34	0,02	4117	-0,34	0,49	6,12	-0,31	0,01
11	331	-0,27	0,49	6,73	-0,34	0,02	4382	-0,32	0,50	6,73	-0,31	0,01
12	381	-0,38	0,41	7,35	-0,34	0,02	4936	-0,32	0,49	7,35	-0,32	0,01
13	463	-0,27	0,47	7,96	-0,35	0,03	5339	-0,33	0,50	7,96	-0,32	0,00
14	507	-0,24	0,49	8,57	-0,35	0,03	5474	-0,31	0,49	8,57	-0,32	0,00
15	540	-0,27	0,54	9,18	-0,34	0,03	5704	-0,32	0,48	9,18	-0,32	0,00
16	490	-0,26	0,58	9,80	-0,34	0,03	5584	-0,30	0,50	9,80	-0,33	0,00
17	622	-0,31	0,46	10,41	-0,34	0,03	5521	-0,30	0,49	10,41	-0,33	0,00
18	576	-0,26	0,51	11,02	-0,33	0,03	5557	-0,31	0,49	11,02	-0,33	0,00
19	561	-0,36	0,49	11,63	-0,33	0,02	5500	-0,30	0,49	11,63	-0,33	0,00
20	604	-0,27	0,50	12,24	-0,32	0,02	5330	-0,30	0,49	12,24	-0,33	0,00
21	506	-0,33	0,47	12,86	-0,32	0,02	4978	-0,29	0,49	12,86	-0,32	0,00
22	429	-0,21	0,46	13,47	-0,31	0,02	4922	-0,29	0,47	13,47	-0,32	0,00
23	503	-0,21	0,46	14,08	-0,31	0,02	4519	-0,27	0,46	14,08	-0,32	0,00
24	418	-0,26	0,55	14,69	-0,30	0,02	3854	-0,29	0,46	14,69	-0,32	0,00
25	360	-0,22	0,52	15,31	-0,29	0,02	3165	-0,27	0,45	15,31	-0,32	0,00
26	278	-0,29	0,49	15,92	-0,29	0,01	2585	-0,24	0,47	15,92	-0,32	0,00
27	245	-0,27	0,54	16,53	-0,28	0,01	2067	-0,21	0,44	16,53	-0,31	0,00
28	153	-0,44	0,50	17,14	-0,28	0,01	1463	-0,21	0,41	17,14	-0,31	0,00
29	120	-0,37	0,47	17,76	-0,27	0,01	1124	-0,18	0,39	17,76	-0,31	0,00
30	87	-0,41	0,37	18,37	-0,27	0,01	645	-0,21	0,42	18,37	-0,30	0,00
				18,98	-0,26	0,01				18,98	-0,30	0,00
				19,59	-0,26	0,01				19,59	-0,30	0,00
				20,20	-0,25	0,01				20,20	-0,29	0,00
				20,82	-0,25	0,01				20,82	-0,29	0,00
				21,43	-0,25	0,02				21,43	-0,29	0,00
				22,04	-0,25	0,02				22,04	-0,28	0,00
				22,65	-0,25	0,02				22,65	-0,28	0,00
				23,27	-0,26	0,02				23,27	-0,27	0,00
				23,88	-0,26	0,02				23,88	-0,27	0,00
				24,49	-0,27	0,02				24,49	-0,26	0,00
				25,10	-0,27	0,02				25,10	-0,26	0,00
				25,71	-0,28	0,02				25,71	-0,25	0,00
				26,33	-0,30	0,02				26,33	-0,24	0,00
				26,94	-0,31	0,02				26,94	-0,23	0,00
				27,55	-0,33	0,02				27,55	-0,23	0,00
				28,16	-0,35	0,03				28,16	-0,22	0,00
				28,78	-0,37	0,03				28,78	-0,21	0,01
				29,39	-0,40	0,04				29,39	-0,20	0,01
				30,00	-0,42	0,06				30,00	-0,19	0,01

© 2024 Chourpiliadis C et al. *JAMA Network Open.*

Anxiety												
Glucose												
	Cases						Controls					
	Observed			Predicted			Observed			Predicted		
Time since index date	Number	Mean	SD	Time since index date	Mean	SE	Number	Mean	SD	Time since index date	Mean	SE
0	12	5,55	1,81	0,00	5,77	0,34	143	5,11	0,84	0,00	5,09	0,03
1	15	5,89	1,86	0,61	5,71	0,25	197	5,10	0,96	0,61	5,09	0,02
2	26	5,42	1,24	1,22	5,65	0,20	264	5,03	0,83	1,22	5,09	0,02
3	33	6,14	3,13	1,84	5,60	0,16	423	5,08	1,17	1,84	5,08	0,01
4	49	5,23	1,11	2,45	5,54	0,14	640	5,08	1,26	2,45	5,08	0,01
5	106	5,19	1,06	3,06	5,49	0,13	988	5,03	1,13	3,06	5,07	0,01
6	138	5,02	1,03	3,67	5,43	0,13	1498	5,04	1,20	3,67	5,07	0,01
7	193	5,14	1,31	4,29	5,39	0,12	1943	5,02	1,04	4,29	5,06	0,01
8	197	5,04	0,87	4,90	5,34	0,11	2383	4,99	1,06	4,90	5,05	0,01
9	303	5,32	2,24	5,51	5,29	0,10	2993	5,00	1,11	5,51	5,04	0,01
10	373	5,09	1,61	6,12	5,25	0,09	3572	4,97	1,07	6,12	5,03	0,01
11	420	4,96	1,03	6,73	5,22	0,08	4293	4,98	1,25	6,73	5,02	0,01
12	500	5,00	1,25	7,35	5,18	0,07	5011	4,94	1,03	7,35	5,01	0,01
13	597	4,99	1,12	7,96	5,15	0,06	5582	4,94	1,14	7,96	5,00	0,01
14	660	4,93	1,12	8,57	5,12	0,05	6137	4,90	1,00	8,57	4,99	0,01
15	715	5,02	1,25	9,18	5,09	0,05	6773	4,88	0,96	9,18	4,98	0,00
16	704	4,86	0,84	9,80	5,06	0,05	6863	4,86	0,91	9,80	4,97	0,00
17	764	4,94	1,33	10,41	5,04	0,04	7381	4,86	1,00	10,41	4,96	0,00
18	774	4,80	0,72	11,02	5,02	0,04	7753	4,86	1,00	11,02	4,95	0,00
19	836	4,85	0,92	11,63	5,00	0,03	7768	4,86	1,00	11,63	4,94	0,00
20	814	4,81	0,67	12,24	4,98	0,03	7884	4,85	0,96	12,24	4,93	0,00
21	780	4,86	1,00	12,86	4,97	0,03	7739	4,85	0,92	12,86	4,92	0,00
22	796	4,82	0,85	13,47	4,95	0,03	7796	4,87	0,95	13,47	4,92	0,00
23	772	4,87	1,03	14,08	4,94	0,03	7697	4,86	1,03	14,08	4,91	0,00
24	683	4,79	0,88	14,69	4,93	0,02	6918	4,83	1,00	14,69	4,90	0,00
25	601	4,84	1,19	15,31	4,92	0,02	6245	4,82	0,95	15,31	4,89	0,00
26	553	4,80	1,05	15,92	4,91	0,02	5420	4,78	0,91	15,92	4,89	0,00
27	484	4,74	0,71	16,53	4,90	0,02	4620	4,74	0,92	16,53	4,88	0,00
28	448	4,74	0,80	17,14	4,89	0,02	4075	4,73	0,84	17,14	4,88	0,00
29	332	4,70	0,62	17,76	4,88	0,02	3319	4,72	0,88	17,76	4,87	0,00
30	290	4,74	0,72	18,37	4,87	0,02	2604	4,72	0,84	18,37	4,87	0,00
				18,98	4,87	0,02				18,98	4,86	0,00
				19,59	4,86	0,02				19,59	4,86	0,00
				20,20	4,85	0,01				20,20	4,85	0,00
				20,82	4,85	0,01				20,82	4,85	0,00
				21,43	4,84	0,01				21,43	4,84	0,00
				22,04	4,83	0,01				22,04	4,84	0,00
				22,65	4,83	0,01				22,65	4,83	0,00
				23,27	4,82	0,01				23,27	4,83	0,00
				23,88	4,81	0,01				23,88	4,82	0,00
				24,49	4,80	0,01				24,49	4,81	0,00
				25,10	4,79	0,01				25,10	4,80	0,00
				25,71	4,79	0,01				25,71	4,80	0,00
				26,33	4,78	0,01				26,33	4,79	0,00
				26,94	4,77	0,01				26,94	4,77	0,00
				27,55	4,76	0,01				27,55	4,76	0,00
				28,16	4,75	0,01				28,16	4,75	0,00
				28,78	4,74	0,01				28,78	4,73	0,00
				29,39	4,73	0,02				29,39	4,71	0,01
				30,00	4,71	0,02				30,00	4,69	0,01

© 2024 Chourpiliadis C et al. *JAMA Network Open.*

Anxiety												
Total Cholesterol												
	Cases						Controls					
	Observed			Predicted			Observed			Predicted		
Time since index date	Number	Mean	SD	Time since index date	Mean	SE	Number	Mean	SD	Time since index date	Mean	SE
0	15	5,96	1,46	0,00	5,81	0,04	153	5,71	1,10	0,00	5,69	0,02
1	15	5,55	0,98	0,61	5,81	0,04	211	5,69	1,05	0,61	5,69	0,02
2	27	5,73	1,18	1,22	5,81	0,03	286	5,64	1,12	1,22	5,68	0,01
3	33	5,94	1,24	1,84	5,81	0,03	434	5,63	1,07	1,84	5,67	0,01
4	52	5,93	1,68	2,45	5,80	0,04	678	5,67	1,17	2,45	5,66	0,01
5	126	5,79	1,12	3,06	5,79	0,04	1059	5,61	1,11	3,06	5,65	0,01
6	145	5,52	1,15	3,67	5,78	0,05	1581	5,59	1,17	3,67	5,64	0,01
7	217	5,81	1,36	4,29	5,77	0,05	2036	5,58	1,17	4,29	5,63	0,01
8	207	5,65	1,21	4,90	5,76	0,05	2536	5,59	1,18	4,90	5,62	0,01
9	330	5,57	1,22	5,51	5,74	0,06	3181	5,56	1,16	5,51	5,61	0,01
10	391	5,62	1,11	6,12	5,72	0,05	3751	5,53	1,14	6,12	5,60	0,01
11	455	5,65	1,22	6,73	5,71	0,05	4541	5,52	1,16	6,73	5,59	0,01
12	538	5,54	1,15	7,35	5,69	0,05	5281	5,53	1,16	7,35	5,58	0,01
13	624	5,55	1,15	7,96	5,67	0,04	5892	5,51	1,17	7,96	5,57	0,01
14	682	5,51	1,19	8,57	5,66	0,04	6459	5,51	1,15	8,57	5,56	0,01
15	762	5,59	1,24	9,18	5,64	0,03	7117	5,48	1,11	9,18	5,55	0,01
16	743	5,54	1,12	9,80	5,62	0,03	7242	5,49	1,14	9,80	5,54	0,00
17	810	5,56	1,17	10,41	5,61	0,02	7806	5,47	1,13	10,41	5,53	0,00
18	825	5,47	1,17	11,02	5,60	0,02	8161	5,47	1,12	11,02	5,52	0,00
19	884	5,52	1,20	11,63	5,58	0,02	8161	5,46	1,14	11,63	5,52	0,00
20	850	5,53	1,15	12,24	5,57	0,02	8284	5,46	1,14	12,24	5,51	0,00
21	825	5,57	1,12	12,86	5,56	0,02	8136	5,50	1,14	12,86	5,50	0,00
22	837	5,55	1,15	13,47	5,55	0,02	8180	5,49	1,15	13,47	5,50	0,00
23	814	5,52	1,16	14,08	5,54	0,02	8082	5,52	1,15	14,08	5,49	0,00
24	719	5,55	1,10	14,69	5,54	0,01	7291	5,50	1,13	14,69	5,49	0,00
25	633	5,55	1,10	15,31	5,53	0,01	6547	5,52	1,14	15,31	5,49	0,00
26	570	5,49	1,19	15,92	5,53	0,01	5695	5,53	1,17	15,92	5,48	0,00
27	507	5,54	1,15	16,53	5,53	0,01	4802	5,49	1,09	16,53	5,48	0,00
28	460	5,53	1,21	17,14	5,52	0,01	4261	5,51	1,11	17,14	5,48	0,00
29	351	5,44	1,18	17,76	5,52	0,01	3425	5,50	1,15	17,76	5,48	0,00
30	298	5,50	1,17	18,37	5,52	0,01	2686	5,47	1,15	18,37	5,48	0,00
				18,98	5,52	0,01				18,98	5,48	0,00
				19,59	5,52	0,01				19,59	5,48	0,00
				20,20	5,53	0,01				20,20	5,49	0,00
				20,82	5,53	0,01				20,82	5,49	0,00
				21,43	5,53	0,01				21,43	5,49	0,00
				22,04	5,53	0,01				22,04	5,50	0,00
				22,65	5,54	0,01				22,65	5,50	0,01
				23,27	5,54	0,01				23,27	5,50	0,01
				23,88	5,54	0,01				23,88	5,51	0,01
				24,49	5,54	0,01				24,49	5,51	0,01
				25,10	5,54	0,01				25,10	5,51	0,01
				25,71	5,54	0,01				25,71	5,51	0,01
				26,33	5,53	0,01				26,33	5,51	0,01
				26,94	5,53	0,01				26,94	5,51	0,01
				27,55	5,52	0,02				27,55	5,51	0,01
				28,16	5,51	0,02				28,16	5,50	0,01
				28,78	5,50	0,02				28,78	5,50	0,01
				29,39	5,48	0,02				29,39	5,49	0,01
				30,00	5,47	0,03				30,00	5,48	0,01

© 2024 Chourpiliadis C et al. *JAMA Network Open.*

Anxiety												
Log2 Triglyceriders												
	Cases						Controls					
	Observed			Predicted			Observed			Predicted		
Time since index date	Number	Mean	SD	Time since index date	Mean	SE	Number	Mean	SD	Time since index date	Mean	SE
0	15	0,70	1,19	0,00	0,54	0,05	153	0,02	0,76	0,00	0,11	0,01
1	15	0,24	0,71	0,61	0,51	0,04	210	0,23	0,82	0,61	0,12	0,01
2	27	0,45	0,85	1,22	0,48	0,03	284	0,19	0,79	1,22	0,13	0,01
3	33	0,45	0,79	1,84	0,45	0,03	433	0,13	0,80	1,84	0,14	0,01
4	52	0,40	0,91	2,45	0,43	0,03	674	0,12	0,81	2,45	0,15	0,01
5	124	0,33	0,76	3,06	0,40	0,03	1044	0,12	0,81	3,06	0,15	0,01
6	145	0,26	0,83	3,67	0,38	0,03	1568	0,17	0,83	3,67	0,15	0,01
7	217	0,35	0,90	4,29	0,36	0,03	2022	0,12	0,79	4,29	0,15	0,01
8	206	0,25	0,81	4,90	0,34	0,03	2505	0,13	0,82	4,90	0,15	0,01
9	323	0,23	0,77	5,51	0,32	0,03	3162	0,13	0,81	5,51	0,15	0,01
10	386	0,24	0,82	6,12	0,30	0,02	3728	0,11	0,83	6,12	0,14	0,01
11	447	0,23	0,88	6,73	0,29	0,02	4503	0,10	0,82	6,73	0,14	0,01
12	530	0,16	0,81	7,35	0,27	0,02	5249	0,08	0,81	7,35	0,13	0,01
13	614	0,11	0,84	7,96	0,26	0,02	5868	0,07	0,80	7,96	0,13	0,01
14	678	0,13	0,85	8,57	0,24	0,01	6433	0,07	0,80	8,57	0,12	0,01
15	751	0,14	0,82	9,18	0,23	0,01	7084	0,04	0,81	9,18	0,12	0,00
16	738	0,11	0,79	9,80	0,22	0,01	7201	0,05	0,80	9,80	0,11	0,00
17	804	0,17	0,85	10,41	0,20	0,01	7752	0,04	0,80	10,41	0,10	0,00
18	812	0,08	0,82	11,02	0,19	0,01	8125	0,05	0,81	11,02	0,10	0,00
19	871	0,08	0,79	11,63	0,18	0,01	8110	0,04	0,81	11,63	0,09	0,00
20	848	0,08	0,86	12,24	0,17	0,01	8240	0,04	0,79	12,24	0,08	0,00
21	816	0,10	0,83	12,86	0,16	0,01	8092	0,05	0,80	12,86	0,08	0,00
22	835	0,06	0,80	13,47	0,15	0,01	8147	0,04	0,79	13,47	0,07	0,00
23	808	0,09	0,79	14,08	0,15	0,01	8042	0,04	0,80	14,08	0,07	0,00
24	718	0,07	0,75	14,69	0,14	0,01	7270	0,03	0,79	14,69	0,06	0,00
25	633	0,08	0,76	15,31	0,13	0,01	6521	0,02	0,80	15,31	0,06	0,00
26	568	0,07	0,83	15,92	0,13	0,01	5679	0,00	0,80	15,92	0,05	0,00
27	505	0,03	0,82	16,53	0,12	0,01	4798	-0,01	0,80	16,53	0,05	0,00
28	460	0,03	0,77	17,14	0,11	0,01	4248	0,01	0,78	17,14	0,05	0,00
29	351	-0,06	0,70	17,76	0,11	0,01	3422	-0,01	0,79	17,76	0,04	0,00
30	297	0,04	0,76	18,37	0,10	0,01	2685	0,02	0,79	18,37	0,04	0,00
				18,98	0,10	0,01				18,98	0,04	0,00
				19,59	0,09	0,01				19,59	0,04	0,00
				20,20	0,09	0,01				20,20	0,03	0,00
				20,82	0,09	0,01				20,82	0,03	0,00
				21,43	0,08	0,01				21,43	0,03	0,00
				22,04	0,08	0,01				22,04	0,03	0,00
				22,65	0,08	0,01				22,65	0,03	0,00
				23,27	0,07	0,01				23,27	0,03	0,00
				23,88	0,07	0,01				23,88	0,03	0,00
				24,49	0,06	0,01				24,49	0,02	0,00
				25,10	0,06	0,01				25,10	0,02	0,00
				25,71	0,05	0,01				25,71	0,02	0,00
				26,33	0,05	0,01				26,33	0,02	0,00
				26,94	0,04	0,01				26,94	0,01	0,00
				27,55	0,03	0,01				27,55	0,01	0,01
				28,16	0,02	0,01				28,16	0,01	0,01
				28,78	0,01	0,02				28,78	0,00	0,01
				29,39	0,00	0,02				29,39	0,00	0,01
				30,00	-0,01	0,03				30,00	-0,01	0,01

© 2024 Chourpiliadis C et al. *JAMA Network Open.*

Anxiety												
LDL-C												
	Cases						Controls					
	Observed			Predicted			Observed			Predicted		
Time since index date	Number	Mean	SD	Time since index date	Mean	SE	Number	Mean	SD	Time since index date	Mean	SE
0	7	4,06	1,02	0,00	3,98	0,18	65	3,62	0,95	0,00	3,60	0,03
1	5	3,78	0,71	0,61	3,94	0,14	85	3,62	0,99	0,61	3,61	0,02
2	9	3,64	0,92	1,22	3,90	0,11	111	3,62	1,14	1,22	3,63	0,02
3	11	4,19	1,07	1,84	3,86	0,10	168	3,60	0,99	1,84	3,64	0,02
4	25	3,80	0,86	2,45	3,83	0,09	263	3,70	1,06	2,45	3,64	0,01
5	54	3,69	1,14	3,06	3,80	0,09	399	3,63	1,04	3,06	3,65	0,01
6	63	3,50	1,12	3,67	3,77	0,09	616	3,63	1,13	3,67	3,65	0,01
7	98	3,87	1,19	4,29	3,75	0,09	772	3,64	1,09	4,29	3,65	0,01
8	75	3,53	1,13	4,90	3,72	0,08	960	3,59	1,09	4,90	3,64	0,01
9	132	3,56	1,15	5,51	3,70	0,08	1183	3,59	1,06	5,51	3,64	0,01
10	143	3,58	1,08	6,12	3,68	0,07	1442	3,57	1,06	6,12	3,63	0,01
11	137	3,69	1,03	6,73	3,67	0,07	1721	3,54	1,08	6,73	3,62	0,01
12	190	3,54	0,99	7,35	3,65	0,06	1884	3,60	1,10	7,35	3,62	0,01
13	199	3,56	1,06	7,96	3,64	0,06	2126	3,55	1,13	7,96	3,61	0,01
14	213	3,51	1,05	8,57	3,63	0,05	2229	3,56	1,10	8,57	3,60	0,01
15	222	3,82	1,20	9,18	3,62	0,05	2398	3,48	1,04	9,18	3,59	0,01
16	274	3,56	1,05	9,80	3,62	0,04	2499	3,54	1,08	9,80	3,58	0,01
17	257	3,58	1,13	10,41	3,61	0,04	2625	3,51	1,07	10,41	3,57	0,01
18	262	3,57	1,13	11,02	3,61	0,04	2772	3,50	1,05	11,02	3,56	0,01
19	273	3,57	1,07	11,63	3,60	0,04	2740	3,51	1,08	11,63	3,55	0,01
20	271	3,53	1,06	12,24	3,60	0,04	2645	3,52	1,10	12,24	3,55	0,01
21	269	3,70	1,11	12,86	3,60	0,04	2563	3,57	1,08	12,86	3,54	0,01
22	260	3,63	1,13	13,47	3,60	0,04	2557	3,55	1,10	13,47	3,54	0,01
23	273	3,52	1,08	14,08	3,60	0,04	2651	3,57	1,09	14,08	3,53	0,01
24	215	3,59	1,14	14,69	3,60	0,04	2301	3,54	1,09	14,69	3,53	0,01
25	176	3,52	1,04	15,31	3,60	0,04	1994	3,53	1,05	15,31	3,52	0,01
26	161	3,33	1,00	15,92	3,60	0,04	1653	3,61	1,17	15,92	3,52	0,01
27	156	3,64	1,13	16,53	3,59	0,03	1310	3,58	1,05	16,53	3,52	0,01
28	125	3,55	1,16	17,14	3,59	0,03	1111	3,54	1,10	17,14	3,52	0,01
29	103	3,47	1,12	17,76	3,59	0,03	907	3,49	1,10	17,76	3,52	0,01
30	66	3,53	1,31	18,37	3,59	0,03	587	3,50	1,04	18,37	3,52	0,01
				18,98	3,59	0,03				18,98	3,53	0,01
				19,59	3,59	0,03				19,59	3,53	0,01
				20,20	3,58	0,03				20,20	3,54	0,01
				20,82	3,58	0,03				20,82	3,54	0,01
				21,43	3,57	0,03				21,43	3,55	0,01
				22,04	3,57	0,03				22,04	3,55	0,01
				22,65	3,56	0,03				22,65	3,55	0,01
				23,27	3,56	0,03				23,27	3,56	0,01
				23,88	3,55	0,04				23,88	3,56	0,01
				24,49	3,55	0,04				24,49	3,57	0,01
				25,10	3,54	0,04				25,10	3,57	0,01
				25,71	3,53	0,04				25,71	3,57	0,01
				26,33	3,53	0,04				26,33	3,56	0,01
				26,94	3,52	0,04				26,94	3,56	0,01
				27,55	3,52	0,05				27,55	3,55	0,01
				28,16	3,51	0,05				28,16	3,54	0,01
				28,78	3,51	0,06				28,78	3,53	0,01
				29,39	3,51	0,07				29,39	3,51	0,01
				30,00	3,51	0,09				30,00	3,49	0,02

© 2024 Chourpiliadis C et al. *JAMA Network Open.*

Anxiety												
HDL-C												
	Cases						Controls					
	Observed			Predicted			Observed			Predicted		
Time since index date	Number	Mean	SD	Time since index date	Mean	SE	Number	Mean	SD	Time since index date	Mean	SE
0	7	1,22	0,23	0,00	1,36	0,04	69	1,65	0,42	0,00	1,61	0,02
1	5	1,53	0,29	0,61	1,41	0,03	85	1,55	0,41	0,61	1,60	0,02
2	8	1,57	0,30	1,22	1,44	0,03	110	1,56	0,39	1,22	1,59	0,01
3	12	1,54	0,34	1,84	1,48	0,03	168	1,59	0,43	1,84	1,58	0,01
4	25	1,59	0,42	2,45	1,50	0,02	271	1,53	0,36	2,45	1,57	0,01
5	55	1,64	0,39	3,06	1,52	0,03	414	1,59	0,41	3,06	1,57	0,01
6	65	1,45	0,39	3,67	1,54	0,03	637	1,57	0,38	3,67	1,56	0,01
7	100	1,50	0,38	4,29	1,55	0,03	808	1,55	0,39	4,29	1,56	0,01
8	76	1,54	0,42	4,90	1,56	0,02	1010	1,56	0,41	4,90	1,56	0,01
9	135	1,55	0,39	5,51	1,57	0,02	1241	1,55	0,38	5,51	1,56	0,01
10	147	1,54	0,40	6,12	1,57	0,02	1496	1,56	0,40	6,12	1,56	0,01
11	147	1,56	0,38	6,73	1,57	0,02	1795	1,56	0,38	6,73	1,56	0,01
12	199	1,57	0,39	7,35	1,57	0,02	1978	1,57	0,38	7,35	1,56	0,01
13	204	1,62	0,38	7,96	1,57	0,02	2219	1,57	0,39	7,96	1,56	0,01
14	220	1,55	0,41	8,57	1,57	0,01	2296	1,58	0,39	8,57	1,56	0,00
15	228	1,60	0,37	9,18	1,57	0,01	2476	1,60	0,39	9,18	1,56	0,00
16	282	1,58	0,38	9,80	1,57	0,01	2552	1,58	0,38	9,80	1,56	0,00
17	271	1,55	0,38	10,41	1,57	0,01	2706	1,58	0,38	10,41	1,56	0,00
18	266	1,55	0,36	11,02	1,57	0,01	2792	1,58	0,39	11,02	1,56	0,00
19	281	1,59	0,40	11,63	1,56	0,01	2788	1,58	0,38	11,63	1,57	0,00
20	278	1,58	0,39	12,24	1,56	0,01	2715	1,57	0,38	12,24	1,57	0,00
21	278	1,58	0,41	12,86	1,56	0,01	2619	1,59	0,39	12,86	1,57	0,00
22	263	1,60	0,40	13,47	1,56	0,01	2626	1,59	0,39	13,47	1,57	0,00
23	268	1,59	0,38	14,08	1,56	0,01	2695	1,60	0,39	14,08	1,57	0,00
24	215	1,63	0,36	14,69	1,56	0,01	2315	1,60	0,39	14,69	1,57	0,00
25	177	1,62	0,36	15,31	1,56	0,01	1996	1,61	0,38	15,31	1,58	0,00
26	157	1,64	0,34	15,92	1,56	0,01	1637	1,60	0,38	15,92	1,58	0,00
27	151	1,66	0,39	16,53	1,56	0,01	1262	1,61	0,38	16,53	1,58	0,00
28	120	1,61	0,34	17,14	1,56	0,01	1069	1,62	0,38	17,14	1,58	0,00
29	101	1,68	0,35	17,76	1,57	0,01	871	1,63	0,38	17,76	1,58	0,00
30	59	1,65	0,39	18,37	1,57	0,01	566	1,61	0,37	18,37	1,58	0,00
				18,98	1,57	0,01				18,98	1,58	0,00
				19,59	1,58	0,01				19,59	1,59	0,00
				20,20	1,58	0,01				20,20	1,59	0,00
				20,82	1,59	0,01				20,82	1,59	0,00
				21,43	1,59	0,01				21,43	1,59	0,00
				22,04	1,60	0,01				22,04	1,59	0,00
				22,65	1,60	0,01				22,65	1,59	0,00
				23,27	1,61	0,01				23,27	1,59	0,00
				23,88	1,61	0,01				23,88	1,60	0,00
				24,49	1,62	0,01				24,49	1,60	0,00
				25,10	1,63	0,01				25,10	1,60	0,00
				25,71	1,63	0,01				25,71	1,60	0,00
				26,33	1,64	0,01				26,33	1,60	0,00
				26,94	1,64	0,01				26,94	1,61	0,00
				27,55	1,65	0,01				27,55	1,61	0,00
				28,16	1,65	0,01				28,16	1,61	0,00
				28,78	1,65	0,02				28,78	1,62	0,00
				29,39	1,66	0,02				29,39	1,62	0,00
				30,00	1,66	0,03				30,00	1,62	0,00

© 2024 Chourpiliadis C et al. *JAMA Network Open.*

Anxiety												
ApoA-I												
	Cases						Controls					
	Observed			Predicted			Observed			Predicted		
Time since index date	Number	Mean	SD	Time since index date	Mean	SE	Number	Mean	SD	Time since index date	Mean	SE
0	5	1,35	0,24	0,00	1,39	0,03	60	1,49	0,24	0,00	1,47	0,01
1	4	1,39	0,14	0,61	1,41	0,02	76	1,46	0,24	0,61	1,46	0,01
2	9	1,50	0,17	1,22	1,42	0,02	102	1,41	0,20	1,22	1,45	0,01
3	11	1,51	0,18	1,84	1,43	0,02	151	1,43	0,23	1,84	1,44	0,01
4	23	1,44	0,26	2,45	1,44	0,01	225	1,41	0,20	2,45	1,44	0,01
5	45	1,49	0,25	3,06	1,45	0,01	322	1,44	0,23	3,06	1,43	0,01
6	48	1,40	0,25	3,67	1,45	0,01	495	1,44	0,21	3,67	1,43	0,01
7	74	1,42	0,23	4,29	1,46	0,01	629	1,42	0,21	4,29	1,43	0,01
8	57	1,42	0,27	4,90	1,46	0,01	794	1,42	0,22	4,90	1,42	0,00
9	101	1,45	0,24	5,51	1,46	0,01	950	1,42	0,21	5,51	1,42	0,00
10	102	1,44	0,23	6,12	1,46	0,01	1184	1,42	0,22	6,12	1,42	0,00
11	116	1,43	0,21	6,73	1,46	0,01	1428	1,41	0,21	6,73	1,42	0,00
12	158	1,45	0,22	7,35	1,45	0,01	1605	1,42	0,21	7,35	1,42	0,00
13	169	1,47	0,22	7,96	1,45	0,01	1824	1,42	0,23	7,96	1,42	0,00
14	187	1,42	0,23	8,57	1,45	0,01	1892	1,42	0,22	8,57	1,42	0,00
15	192	1,44	0,23	9,18	1,45	0,01	2074	1,43	0,23	9,18	1,42	0,00
16	243	1,44	0,22	9,80	1,44	0,01	2150	1,42	0,22	9,80	1,42	0,00
17	239	1,42	0,21	10,41	1,44	0,01	2297	1,42	0,22	10,41	1,42	0,00
18	229	1,41	0,21	11,02	1,44	0,01	2389	1,42	0,22	11,02	1,42	0,00
19	255	1,43	0,23	11,63	1,44	0,01	2410	1,43	0,22	11,63	1,42	0,00
20	254	1,44	0,22	12,24	1,43	0,01	2325	1,42	0,22	12,24	1,42	0,00
21	249	1,46	0,23	12,86	1,43	0,01	2267	1,43	0,23	12,86	1,42	0,00
22	238	1,44	0,25	13,47	1,43	0,01	2287	1,43	0,22	13,47	1,42	0,00
23	250	1,42	0,22	14,08	1,43	0,01	2389	1,43	0,23	14,08	1,42	0,00
24	193	1,44	0,21	14,69	1,43	0,01	2090	1,43	0,22	14,69	1,42	0,00
25	167	1,44	0,22	15,31	1,43	0,01	1863	1,43	0,21	15,31	1,42	0,00
26	160	1,45	0,22	15,92	1,43	0,01	1597	1,42	0,22	15,92	1,42	0,00
27	157	1,46	0,23	16,53	1,43	0,01	1292	1,42	0,22	16,53	1,42	0,00
28	124	1,43	0,21	17,14	1,43	0,00	1110	1,43	0,23	17,14	1,42	0,00
29	103	1,47	0,21	17,76	1,43	0,00	908	1,43	0,23	17,76	1,42	0,00
30	66	1,42	0,25	18,37	1,43	0,00	588	1,43	0,21	18,37	1,42	0,00
				18,98	1,43	0,00				18,98	1,42	0,00
				19,59	1,43	0,00				19,59	1,43	0,00
				20,20	1,43	0,00				20,20	1,43	0,00
				20,82	1,43	0,00				20,82	1,43	0,00
				21,43	1,44	0,00				21,43	1,43	0,00
				22,04	1,44	0,00				22,04	1,43	0,00
				22,65	1,44	0,00				22,65	1,43	0,00
				23,27	1,44	0,01				23,27	1,43	0,00
				23,88	1,44	0,01				23,88	1,43	0,00
				24,49	1,45	0,01				24,49	1,43	0,00
				25,10	1,45	0,01				25,10	1,43	0,00
				25,71	1,45	0,01				25,71	1,43	0,00
				26,33	1,45	0,01				26,33	1,43	0,00
				26,94	1,45	0,01				26,94	1,43	0,00
				27,55	1,45	0,01				27,55	1,43	0,00
				28,16	1,45	0,01				28,16	1,43	0,00
				28,78	1,44	0,01				28,78	1,43	0,00
				29,39	1,44	0,01				29,39	1,43	0,00
				30,00	1,44	0,02				30,00	1,43	0,00

© 2024 Chourpiliadis C et al. *JAMA Network Open.*

Anxiety												
ApoB												
	Cases						Controls					
	Observed			Predicted			Observed			Predicted		
Time since index date	Number	Mean	SD	Time since index date	Mean	SE	Number	Mean	SD	Time since index date	Mean	SE
0	5	1,31	0,46	0,00	1,34	0,05	64	1,06	0,26	0,00	1,11	0,01
1	4	1,38	0,53	0,61	1,32	0,04	69	1,15	0,34	0,61	1,12	0,01
2	10	1,24	0,36	1,22	1,31	0,03	98	1,19	0,40	1,22	1,13	0,01
3	11	1,30	0,36	1,84	1,29	0,03	142	1,17	0,32	1,84	1,14	0,00
4	22	1,25	0,35	2,45	1,28	0,02	214	1,17	0,33	2,45	1,15	0,00
5	42	1,20	0,33	3,06	1,26	0,02	328	1,14	0,33	3,06	1,15	0,00
6	47	1,15	0,37	3,67	1,25	0,02	482	1,14	0,35	3,67	1,16	0,00
7	71	1,26	0,41	4,29	1,24	0,02	605	1,14	0,36	4,29	1,16	0,00
8	51	1,22	0,43	4,90	1,23	0,02	806	1,15	0,36	4,90	1,16	0,00
9	92	1,15	0,36	5,51	1,22	0,02	964	1,14	0,35	5,51	1,16	0,00
10	96	1,15	0,37	6,12	1,21	0,02	1158	1,15	0,36	6,12	1,16	0,00
11	115	1,19	0,35	6,73	1,20	0,02	1369	1,14	0,35	6,73	1,16	0,00
12	135	1,17	0,36	7,35	1,19	0,02	1551	1,15	0,36	7,35	1,16	0,00
13	161	1,13	0,35	7,96	1,19	0,02	1769	1,15	0,37	7,96	1,16	0,00
14	176	1,14	0,35	8,57	1,18	0,01	1800	1,16	0,35	8,57	1,15	0,00
15	179	1,20	0,37	9,18	1,18	0,01	1915	1,14	0,35	9,18	1,15	0,00
16	229	1,19	0,35	9,80	1,17	0,01	2043	1,16	0,36	9,80	1,15	0,00
17	236	1,19	0,37	10,41	1,17	0,01	2108	1,16	0,37	10,41	1,15	0,00
18	211	1,19	0,40	11,02	1,17	0,01	2221	1,17	0,35	11,02	1,15	0,00
19	235	1,19	0,36	11,63	1,17	0,01	2146	1,17	0,35	11,63	1,15	0,00
20	222	1,16	0,35	12,24	1,17	0,01	2162	1,16	0,36	12,24	1,15	0,00
21	229	1,21	0,35	12,86	1,17	0,01	2080	1,19	0,36	12,86	1,15	0,00
22	214	1,19	0,37	13,47	1,17	0,01	2123	1,18	0,36	13,47	1,15	0,00
23	212	1,21	0,35	14,08	1,17	0,01	2112	1,19	0,36	14,08	1,15	0,00
24	171	1,22	0,35	14,69	1,17	0,01	1823	1,19	0,37	14,69	1,15	0,00
25	150	1,22	0,33	15,31	1,17	0,01	1651	1,21	0,35	15,31	1,15	0,00
26	131	1,18	0,30	15,92	1,17	0,01	1334	1,24	0,37	15,92	1,15	0,00
27	126	1,25	0,35	16,53	1,17	0,01	1063	1,23	0,34	16,53	1,15	0,00
28	97	1,23	0,33	17,14	1,18	0,01	876	1,24	0,34	17,14	1,16	0,00
29	74	1,23	0,30	17,76	1,18	0,01	651	1,24	0,33	17,76	1,16	0,00
30	51	1,22	0,30	18,37	1,18	0,01	475	1,26	0,30	18,37	1,16	0,00
				18,98	1,19	0,01				18,98	1,17	0,00
				19,59	1,19	0,01				19,59	1,17	0,00
				20,20	1,19	0,01				20,20	1,17	0,00
				20,82	1,19	0,01				20,82	1,18	0,00
				21,43	1,20	0,01				21,43	1,18	0,00
				22,04	1,20	0,01				22,04	1,19	0,00
				22,65	1,20	0,01				22,65	1,19	0,00
				23,27	1,21	0,01				23,27	1,20	0,00
				23,88	1,21	0,01				23,88	1,20	0,00
				24,49	1,21	0,01				24,49	1,21	0,00
				25,10	1,22	0,01				25,10	1,22	0,00
				25,71	1,22	0,01				25,71	1,22	0,00
				26,33	1,22	0,01				26,33	1,23	0,00
				26,94	1,22	0,01				26,94	1,23	0,00
				27,55	1,22	0,01				27,55	1,24	0,00
				28,16	1,22	0,01				28,16	1,24	0,00
				28,78	1,23	0,01				28,78	1,25	0,00
				29,39	1,23	0,02				29,39	1,25	0,01
				30,00	1,23	0,02				30,00	1,26	0,01

© 2024 Chourpiliadis C et al. *JAMA Network Open.*

Anxiety												
Log2 LDL-C/HDL-C ratio												
	Cases						Controls					
	Observed			Predicted			Observed			Predicted		
Time since index date	Number	Mean	SD	Time since index date	Mean	SE	Number	Mean	SD	Time since index date	Mean	SE
0	6	1,69	0,38	0,00	1,54	0,10	60	1,10	0,55	0,00	1,14	0,04
1	5	1,31	0,39	0,61	1,48	0,08	80	1,23	0,57	0,61	1,15	0,03
2	8	1,18	0,44	1,22	1,42	0,06	102	1,16	0,62	1,22	1,16	0,02
3	11	1,45	0,64	1,84	1,37	0,06	151	1,15	0,62	1,84	1,17	0,02
4	22	1,21	0,57	2,45	1,33	0,05	249	1,24	0,57	2,45	1,18	0,02
5	54	1,15	0,59	3,06	1,29	0,05	374	1,18	0,59	3,06	1,19	0,02
6	59	1,21	0,61	3,67	1,26	0,05	587	1,17	0,61	3,67	1,19	0,02
7	90	1,35	0,63	4,29	1,24	0,05	730	1,20	0,59	4,29	1,19	0,02
8	70	1,16	0,64	4,90	1,22	0,05	919	1,17	0,61	4,90	1,19	0,01
9	128	1,15	0,65	5,51	1,21	0,04	1137	1,18	0,58	5,51	1,19	0,01
10	139	1,19	0,62	6,12	1,19	0,04	1378	1,17	0,61	6,12	1,19	0,01
11	130	1,21	0,63	6,73	1,19	0,04	1645	1,15	0,59	6,73	1,19	0,01
12	184	1,16	0,59	7,35	1,18	0,03	1782	1,16	0,60	7,35	1,18	0,01
13	187	1,12	0,60	7,96	1,18	0,03	2043	1,14	0,63	7,96	1,18	0,01
14	205	1,15	0,61	8,57	1,17	0,03	2134	1,14	0,60	8,57	1,17	0,01
15	211	1,23	0,57	9,18	1,17	0,02	2294	1,09	0,60	9,18	1,17	0,01
16	265	1,15	0,58	9,80	1,17	0,02	2385	1,13	0,60	9,80	1,16	0,01
17	243	1,17	0,61	10,41	1,17	0,02	2517	1,12	0,60	10,41	1,16	0,00
18	249	1,16	0,62	11,02	1,17	0,02	2627	1,11	0,60	11,02	1,15	0,00
19	257	1,13	0,60	11,63	1,17	0,02	2617	1,11	0,61	11,63	1,15	0,00
20	259	1,12	0,62	12,24	1,17	0,02	2535	1,12	0,61	12,24	1,14	0,00
21	259	1,18	0,64	12,86	1,17	0,02	2440	1,14	0,61	12,86	1,14	0,00
22	247	1,14	0,65	13,47	1,18	0,01	2462	1,12	0,62	13,47	1,13	0,00
23	259	1,10	0,60	14,08	1,18	0,01	2552	1,13	0,62	14,08	1,13	0,00
24	209	1,11	0,58	14,69	1,18	0,01	2231	1,11	0,62	14,69	1,13	0,00
25	173	1,09	0,54	15,31	1,18	0,01	1932	1,10	0,61	15,31	1,12	0,00
26	152	0,97	0,59	15,92	1,17	0,01	1596	1,13	0,63	15,92	1,12	0,00
27	149	1,09	0,58	16,53	1,17	0,01	1253	1,11	0,61	16,53	1,12	0,00
28	120	1,10	0,52	17,14	1,17	0,01	1068	1,09	0,62	17,14	1,12	0,00
29	101	1,00	0,57	17,76	1,17	0,01	871	1,06	0,60	17,76	1,12	0,00
30	59	1,02	0,67	18,37	1,16	0,01	566	1,08	0,59	18,37	1,12	0,00
			0,38	18,98	1,16	0,01				18,98	1,12	0,00
			0,39	19,59	1,15	0,01				19,59	1,12	0,00
			0,44	20,20	1,14	0,01				20,20	1,12	0,00
			0,64	20,82	1,14	0,01				20,82	1,12	0,00
			0,57	21,43	1,13	0,01				21,43	1,12	0,00
			0,59	22,04	1,12	0,01				22,04	1,12	0,00
			0,61	22,65	1,11	0,01				22,65	1,12	0,00
			0,63	23,27	1,11	0,02				23,27	1,12	0,01
			0,64	23,88	1,10	0,02				23,88	1,12	0,01
			0,65	24,49	1,09	0,02				24,49	1,12	0,01
			0,62	25,10	1,08	0,02				25,10	1,12	0,01
			0,63	25,71	1,07	0,02				25,71	1,12	0,01
			0,59	26,33	1,06	0,02				26,33	1,11	0,01
			0,60	26,94	1,05	0,02				26,94	1,11	0,01
			0,61	27,55	1,05	0,02				27,55	1,10	0,01
			0,57	28,16	1,04	0,03				28,16	1,09	0,01
			0,58	28,78	1,03	0,03				28,78	1,09	0,01
			0,61	29,39	1,03	0,04				29,39	1,07	0,01
			0,62	30,00	1,03	0,05				30,00	1,06	0,01

© 2024 Chourpiliadis C et al. *JAMA Network Open.*

Anxiety													
Log2 ApoB/ApoA-I ratio													
	Cases						Controls						
	Observed			Predicted			Observed			Predicted			
Time since index date	Number	Mean	SD	Time since index date	Mean	SE	Number	Mean	SD	Time since index date	Mean	SE	
0	11	-0,29	0,24	0,00	-0,40	0,09	207	-0,62	0,41	0,00	-0,60	0,02	
1	6	-0,39	0,00	0,61	-0,42	0,07	210	-0,53	0,42	0,61	-0,56	0,02	
2	28	-0,64	0,25	1,22	-0,44	0,06	300	-0,50	0,41	1,22	-0,53	0,01	
3	42	-0,56	0,41	1,84	-0,45	0,05	380	-0,40	0,40	1,84	-0,51	0,01	
4	36	-0,38	0,53	2,45	-0,45	0,04	447	-0,42	0,38	2,45	-0,48	0,01	
5	98	-0,56	0,31	3,06	-0,45	0,04	641	-0,45	0,44	3,06	-0,46	0,01	
6	121	-0,41	0,50	3,67	-0,45	0,04	899	-0,44	0,44	3,67	-0,45	0,01	
7	147	-0,28	0,42	4,29	-0,45	0,04	1312	-0,40	0,46	4,29	-0,43	0,01	
8	120	-0,34	0,41	4,90	-0,44	0,04	1585	-0,39	0,48	4,90	-0,42	0,01	
9	184	-0,29	0,62	5,51	-0,43	0,04	1980	-0,41	0,46	5,51	-0,41	0,01	
10	160	-0,43	0,42	6,12	-0,43	0,03	2454	-0,37	0,47	6,12	-0,41	0,01	
11	194	-0,30	0,43	6,73	-0,42	0,03	2902	-0,38	0,48	6,73	-0,40	0,01	
12	232	-0,36	0,45	7,35	-0,41	0,03	3328	-0,37	0,52	7,35	-0,39	0,01	
13	277	-0,42	0,50	7,96	-0,40	0,03	3829	-0,39	0,51	7,96	-0,39	0,01	
14	326	-0,35	0,45	8,57	-0,39	0,02	3714	-0,37	0,49	8,57	-0,39	0,01	
15	296	-0,32	0,52	9,18	-0,39	0,02	3998	-0,39	0,48	9,18	-0,39	0,01	
16	411	-0,34	0,48	9,80	-0,38	0,02	4262	-0,39	0,46	9,80	-0,38	0,01	
17	354	-0,36	0,48	10,41	-0,37	0,02	4482	-0,36	0,49	10,41	-0,38	0,01	
18	437	-0,26	0,46	11,02	-0,36	0,02	4554	-0,35	0,47	11,02	-0,38	0,01	
19	489	-0,30	0,50	11,63	-0,36	0,02	4434	-0,34	0,50	11,63	-0,38	0,01	
20	383	-0,41	0,47	12,24	-0,35	0,02	4165	-0,36	0,51	12,24	-0,38	0,00	
21	414	-0,37	0,45	12,86	-0,35	0,02	4167	-0,35	0,48	12,86	-0,38	0,00	
22	331	-0,32	0,46	13,47	-0,35	0,02	4355	-0,37	0,49	13,47	-0,38	0,00	
23	425	-0,23	0,48	14,08	-0,34	0,02	4491	-0,31	0,50	14,08	-0,38	0,00	
24	332	-0,27	0,43	14,69	-0,34	0,02	3723	-0,32	0,51	14,69	-0,38	0,00	
25	302	-0,25	0,44	15,31	-0,33	0,02	3174	-0,29	0,46	15,31	-0,38	0,01	
26	261	-0,28	0,43	15,92	-0,33	0,02	2532	-0,26	0,47	15,92	-0,38	0,01	
27	207	-0,12	0,41	16,53	-0,33	0,02	2043	-0,27	0,47	16,53	-0,38	0,01	
28	154	-0,21	0,34	17,14	-0,33	0,02	1644	-0,23	0,47	17,14	-0,37	0,01	
29	125	-0,27	0,32	17,76	-0,32	0,02	1098	-0,22	0,43	17,76	-0,37	0,01	
30	74	-0,25	0,38	18,37	-0,32	0,02	735	-0,20	0,41	18,37	-0,37	0,01	
				18,98	-0,32	0,02				18,98	-0,36	0,01	
				19,59	-0,31	0,02				19,59	-0,36	0,01	
				20,20	-0,31	0,02				20,20	-0,35	0,01	
				20,82	-0,30	0,02				20,82	-0,35	0,01	
				21,43	-0,30	0,02				21,43	-0,34	0,01	
				22,04	-0,29	0,02				22,04	-0,33	0,01	
				22,65	-0,29	0,02				22,65	-0,33	0,01	
				23,27	-0,28	0,02				23,27	-0,32	0,01	
				23,88	-0,27	0,02				23,88	-0,31	0,01	
				24,49	-0,27	0,02				24,49	-0,30	0,01	
				25,10	-0,26	0,02				25,10	-0,29	0,01	
				25,71	-0,25	0,02				25,71	-0,28	0,01	
				26,33	-0,25	0,02				26,33	-0,27	0,01	
				26,94	-0,24	0,02				26,94	-0,26	0,01	
				27,55	-0,24	0,03				27,55	-0,25	0,01	
				28,16	-0,23	0,03				28,16	-0,23	0,01	
				28,78	-0,23	0,03				28,78	-0,22	0,01	
				29,39	-0,23	0,04				29,39	-0,21	0,01	
				30,00	-0,23	0,05				30,00	-0,20	0,01	

© 2024 Chourpiliadis C et al. *JAMA Network Open.*

Stress-related disorders												
Glucose												
	Cases						Controls					
	Observed			Predicted			Observed			Predicted		
Time since index date	Number	Mean	SD	Time since index date	Mean	SE	Number	Mean	SD	Time since index date	Mean	SE
0	18	5,42	1,21	0,00	5,15	0,30	170	5,02	0,90	0,00	5,03	0,04
1	15	4,84	0,57	0,61	5,19	0,24	268	4,93	0,70	0,61	5,03	0,03
2	35	4,96	0,83	1,22	5,21	0,20	309	5,12	1,44	1,22	5,03	0,03
3	34	5,09	1,03	1,84	5,23	0,17	418	5,08	1,44	1,84	5,03	0,02
4	50	6,12	3,66	2,45	5,24	0,16	636	5,09	1,47	2,45	5,03	0,02
5	123	5,16	1,57	3,06	5,24	0,15	957	5,01	1,23	3,06	5,03	0,02
6	138	5,08	1,19	3,67	5,23	0,15	1429	4,89	0,91	3,67	5,02	0,02
7	186	5,08	1,87	4,29	5,22	0,15	1798	4,90	0,92	4,29	5,01	0,02
8	173	4,87	0,79	4,90	5,20	0,14	1982	4,90	1,00	4,90	5,00	0,02
9	195	4,97	1,03	5,51	5,18	0,13	2134	4,93	1,13	5,51	4,99	0,02
10	213	4,84	0,71	6,12	5,16	0,12	2344	4,91	1,03	6,12	4,98	0,02
11	247	4,95	1,05	6,73	5,13	0,11	2733	4,91	1,09	6,73	4,96	0,02
12	345	4,94	1,29	7,35	5,11	0,09	2985	4,84	0,89	7,35	4,95	0,02
13	310	4,96	1,53	7,96	5,08	0,08	3330	4,86	1,11	7,96	4,94	0,01
14	358	4,89	1,00	8,57	5,05	0,06	3574	4,83	0,98	8,57	4,93	0,01
15	379	4,87	1,01	9,18	5,02	0,05	3683	4,80	0,94	9,18	4,92	0,01
16	407	4,78	0,80	9,80	5,00	0,04	4037	4,82	1,07	9,80	4,90	0,01
17	418	4,73	0,68	10,41	4,97	0,03	4082	4,79	0,87	10,41	4,89	0,01
18	436	4,80	0,72	11,02	4,95	0,03	4254	4,78	0,94	11,02	4,88	0,01
19	434	4,82	0,86	11,63	4,92	0,02	4656	4,79	0,89	11,63	4,87	0,01
20	471	4,77	0,74	12,24	4,90	0,02	4647	4,77	0,88	12,24	4,86	0,01
21	492	4,76	0,77	12,86	4,88	0,02	4513	4,76	0,88	12,86	4,85	0,01
22	489	4,80	0,89	13,47	4,86	0,02	4426	4,77	0,94	13,47	4,84	0,01
23	462	4,75	0,90	14,08	4,85	0,02	4475	4,72	0,88	14,08	4,83	0,01
24	407	4,74	1,03	14,69	4,83	0,01	4147	4,70	0,89	14,69	4,83	0,01
25	374	4,70	0,97	15,31	4,82	0,01	3646	4,67	0,74	15,31	4,82	0,01
26	347	4,63	0,56	15,92	4,81	0,01	2987	4,66	0,90	15,92	4,81	0,00
27	257	4,58	0,74	16,53	4,80	0,01	2543	4,60	0,76	16,53	4,80	0,00
28	231	4,55	0,65	17,14	4,79	0,01	2144	4,56	0,64	17,14	4,80	0,00
29	170	4,53	0,52	17,76	4,78	0,01	1795	4,56	0,74	17,76	4,79	0,00
30	150	4,48	0,55	18,37	4,78	0,01	1357	4,60	0,76	18,37	4,78	0,00
	18	5,42		18,98	4,77	0,01				18,98	4,77	0,00
	15	4,84		19,59	4,77	0,01				19,59	4,77	0,00
	35	4,96		20,20	4,76	0,01				20,20	4,76	0,00
	34	5,09		20,82	4,76	0,01				20,82	4,75	0,00
	50	6,12		21,43	4,75	0,01				21,43	4,74	0,00
	123	5,16		22,04	4,75	0,01				22,04	4,73	0,00
	138	5,08		22,65	4,74	0,01				22,65	4,72	0,00
	186	5,08		23,27	4,73	0,01				23,27	4,71	0,00
	173	4,87		23,88	4,72	0,01				23,88	4,70	0,01
	195	4,97		24,49	4,71	0,01				24,49	4,69	0,01
	213	4,84		25,10	4,70	0,01				25,10	4,67	0,01
	247	4,95		25,71	4,68	0,01				25,71	4,66	0,01
	345	4,94		26,33	4,66	0,01				26,33	4,65	0,01
	310	4,96		26,94	4,64	0,00				26,94	4,63	0,00
	358	4,89		27,55	4,61	0,00				27,55	4,62	0,01
	379	4,87		28,16	4,57	0,00				28,16	4,60	0,01
	407	4,78		28,78	4,54	0,00				28,78	4,58	0,01
	418	4,73		29,39	4,49	0,01				29,39	4,56	0,01
	436	4,80		30,00	4,44	0,01				30,00	4,55	0,01

© 2024 Chourpiliadis C et al. *JAMA Network Open.*

Stress-related disorders												
Total Cholesterol												
	Cases						Controls					
	Observed			Predicted			Observed			Predicted		
Time since index date	Number	Mean	SD	Time since index date	Mean	SE	Number	Mean	SD	Time since index date	Mean	SE
0	21	6,57	2,15	0,00	6,27	0,18	174	5,75	1,08	0,00	5,82	0,05
1	18	5,63	0,96	0,61	6,12	0,14	288	5,83	1,18	0,61	5,78	0,04
2	36	5,87	1,31	1,22	5,99	0,11	322	5,66	1,11	1,22	5,73	0,03
3	35	5,53	0,94	1,84	5,88	0,09	438	5,66	1,08	1,84	5,69	0,02
4	52	5,77	1,06	2,45	5,78	0,08	662	5,60	1,17	2,45	5,65	0,02
5	124	5,55	1,23	3,06	5,70	0,07	1014	5,53	1,11	3,06	5,61	0,02
6	146	5,55	1,14	3,67	5,63	0,06	1487	5,41	1,12	3,67	5,58	0,02
7	194	5,47	1,16	4,29	5,58	0,06	1895	5,40	1,11	4,29	5,55	0,02
8	181	5,37	1,00	4,90	5,53	0,05	2089	5,39	1,09	4,90	5,52	0,02
9	201	5,33	1,08	5,51	5,50	0,05	2214	5,41	1,11	5,51	5,50	0,01
10	229	5,42	1,25	6,12	5,47	0,04	2453	5,38	1,07	6,12	5,47	0,01
11	260	5,46	1,02	6,73	5,44	0,03	2853	5,36	1,08	6,73	5,45	0,01
12	360	5,39	1,09	7,35	5,43	0,03	3098	5,34	1,08	7,35	5,44	0,01
13	328	5,39	1,09	7,96	5,41	0,02	3511	5,35	1,09	7,96	5,42	0,01
14	374	5,31	1,06	8,57	5,40	0,02	3717	5,30	1,07	8,57	5,40	0,01
15	411	5,39	1,09	9,18	5,39	0,02	3816	5,31	1,09	9,18	5,39	0,01
16	418	5,30	1,02	9,80	5,39	0,02	4194	5,30	1,07	9,80	5,38	0,01
17	444	5,29	1,07	10,41	5,39	0,02	4240	5,27	1,06	10,41	5,37	0,01
18	462	5,24	1,08	11,02	5,38	0,02	4451	5,27	1,07	11,02	5,36	0,01
19	457	5,22	1,11	11,63	5,38	0,02	4834	5,24	1,06	11,63	5,35	0,01
20	489	5,27	1,09	12,24	5,37	0,02	4841	5,22	1,05	12,24	5,34	0,01
21	521	5,12	1,08	12,86	5,37	0,02	4745	5,19	1,06	12,86	5,33	0,01
22	501	5,16	1,11	13,47	5,37	0,01	4612	5,17	1,06	13,47	5,32	0,01
23	481	5,10	1,09	14,08	5,36	0,01	4614	5,17	1,04	14,08	5,31	0,01
24	418	5,02	0,98	14,69	5,35	0,01	4283	5,16	1,04	14,69	5,30	0,00
25	388	5,12	1,16	15,31	5,34	0,01	3764	5,13	1,04	15,31	5,30	0,00
26	352	5,00	0,99	15,92	5,33	0,01	3088	5,12	1,01	15,92	5,29	0,00
27	262	5,05	1,02	16,53	5,32	0,01	2605	5,12	1,05	16,53	5,28	0,00
28	242	5,04	0,96	17,14	5,30	0,01	2215	5,05	1,00	17,14	5,27	0,00
29	175	4,95	0,92	17,76	5,29	0,01	1852	5,05	1,04	17,76	5,26	0,00
30	153	5,00	1,00	18,37	5,27	0,01	1390	5,08	1,04	18,37	5,25	0,00
				18,98	5,25	0,01				18,98	5,24	0,00
				19,59	5,23	0,01				19,59	5,23	0,00
				20,20	5,21	0,02				20,20	5,22	0,00
				20,82	5,19	0,02				20,82	5,21	0,00
				21,43	5,17	0,02				21,43	5,20	0,00
				22,04	5,15	0,02				22,04	5,19	0,00
				22,65	5,12	0,02				22,65	5,17	0,00
				23,27	5,10	0,02				23,27	5,16	0,00
				23,88	5,08	0,02				23,88	5,15	0,00
				24,49	5,06	0,02				24,49	5,14	0,01
				25,10	5,04	0,02				25,10	5,13	0,01
				25,71	5,03	0,02				25,71	5,12	0,01
				26,33	5,01	0,02				26,33	5,11	0,01
				26,94	5,00	0,02				26,94	5,10	0,01
				27,55	5,00	0,02				27,55	5,09	0,01
				28,16	4,99	0,02				28,16	5,08	0,01
				28,78	4,99	0,03				28,78	5,07	0,01
				29,39	5,00	0,03				29,39	5,07	0,01
				30,00	5,01	0,04				30,00	5,06	0,02

© 2024 Chourpiliadis C et al. *JAMA Network Open.*

Stress-related disorders												
Log2 Triglycerides												
	Cases						Controls					
	Observed			Predicted			Observed			Predicted		
Time since index date	Number	Mean	SD	Time since index date	Mean	SE	Number	Mean	SD	Time since index date	Mean	SE
0	19	0,32	0,82	0,00	0,37	0,11	174	0,24	0,89	0,00	0,23	0,03
1	16	0,34	1,09	0,61	0,35	0,09	285	0,16	0,92	0,61	0,21	0,02
2	36	0,40	0,82	1,22	0,33	0,07	323	0,22	0,88	1,22	0,20	0,02
3	35	0,19	1,04	1,84	0,31	0,06	438	0,15	0,84	1,84	0,19	0,02
4	52	0,47	0,88	2,45	0,29	0,05	658	0,16	0,88	2,45	0,17	0,01
5	124	0,08	0,81	3,06	0,27	0,05	1006	0,15	0,83	3,06	0,16	0,01
6	143	0,11	0,81	3,67	0,25	0,05	1480	0,07	0,82	3,67	0,15	0,01
7	194	0,19	0,75	4,29	0,24	0,05	1879	0,12	0,82	4,29	0,14	0,01
8	180	0,14	0,75	4,90	0,22	0,05	2077	0,07	0,80	4,90	0,13	0,01
9	198	0,11	0,80	5,51	0,20	0,05	2209	0,09	0,85	5,51	0,12	0,01
10	225	0,07	0,80	6,12	0,19	0,04	2455	0,07	0,80	6,12	0,12	0,01
11	258	0,13	0,79	6,73	0,17	0,04	2842	0,09	0,83	6,73	0,11	0,01
12	357	0,11	0,85	7,35	0,16	0,03	3079	0,07	0,80	7,35	0,10	0,01
13	326	0,11	0,83	7,96	0,15	0,03	3496	0,04	0,82	7,96	0,09	0,01
14	371	0,08	0,86	8,57	0,14	0,03	3705	0,02	0,81	8,57	0,09	0,01
15	408	0,09	0,82	9,18	0,13	0,02	3803	0,01	0,80	9,18	0,08	0,01
16	414	0,03	0,80	9,80	0,12	0,02	4170	-0,01	0,81	9,80	0,07	0,01
17	434	0,00	0,82	10,41	0,11	0,02	4228	-0,02	0,80	10,41	0,07	0,01
18	458	-0,02	0,77	11,02	0,10	0,02	4437	0,00	0,81	11,02	0,06	0,01
19	448	0,06	0,77	11,63	0,09	0,01	4805	-0,02	0,79	11,63	0,05	0,01
20	484	0,04	0,84	12,24	0,09	0,01	4815	-0,05	0,78	12,24	0,05	0,01
21	519	-0,03	0,86	12,86	0,08	0,01	4711	-0,04	0,79	12,86	0,04	0,00
22	502	0,04	0,84	13,47	0,07	0,01	4575	-0,04	0,78	13,47	0,03	0,00
23	478	0,01	0,82	14,08	0,07	0,01	4594	-0,07	0,78	14,08	0,03	0,00
24	417	-0,07	0,79	14,69	0,06	0,01	4269	-0,08	0,79	14,69	0,02	0,00
25	388	-0,10	0,81	15,31	0,06	0,01	3751	-0,07	0,77	15,31	0,02	0,00
26	351	-0,10	0,80	15,92	0,05	0,01	3074	-0,11	0,78	15,92	0,01	0,00
27	261	-0,08	0,82	16,53	0,05	0,01	2604	-0,12	0,77	16,53	0,00	0,00
28	241	-0,16	0,76	17,14	0,04	0,01	2212	-0,17	0,73	17,14	0,00	0,00
29	175	-0,17	0,79	17,76	0,03	0,01	1851	-0,16	0,73	17,76	-0,01	0,00
30	153	-0,18	0,70	18,37	0,03	0,01	1390	-0,15	0,74	18,37	-0,02	0,00
				18,98	0,02	0,01				18,98	-0,02	0,00
				19,59	0,02	0,01				19,59	-0,03	0,00
				20,20	0,01	0,01				20,20	-0,04	0,00
				20,82	0,00	0,01				20,82	-0,04	0,00
				21,43	-0,01	0,01				21,43	-0,05	0,00
				22,04	-0,01	0,01				22,04	-0,06	0,00
				22,65	-0,02	0,01				22,65	-0,06	0,00
				23,27	-0,03	0,01				23,27	-0,07	0,00
				23,88	-0,04	0,01				23,88	-0,08	0,00
				24,49	-0,06	0,01				24,49	-0,09	0,00
				25,10	-0,07	0,01				25,10	-0,10	0,00
				25,71	-0,08	0,01				25,71	-0,10	0,00
				26,33	-0,10	0,01				26,33	-0,11	0,01
				26,94	-0,11	0,01				26,94	-0,12	0,01
				27,55	-0,13	0,01				27,55	-0,13	0,01
				28,16	-0,14	0,01				28,16	-0,14	0,01
				28,78	-0,16	0,02				28,78	-0,15	0,01
				29,39	-0,18	0,02				29,39	-0,16	0,01
				30,00	-0,20	0,02				30,00	-0,17	0,01

© 2024 Chourpiliadis C et al. *JAMA Network Open.*

Stress-related disorders												
LDL-C												
	Cases						Controls					
	Observed			Predicted			Observed			Predicted		
Time since index date	Number	Mean	SD	Time since index date	Mean	SE	Number	Mean	SD	Time since index date	Mean	SE
0	7	3,93	1,10	0,00	3,84	0,29	73	3,90	1,02	0,00	3,86	0,05
1	7	3,49	1,00	0,61	3,78	0,22	113	3,74	1,14	0,61	3,81	0,04
2	15	3,97	1,18	1,22	3,73	0,17	148	3,62	0,99	1,22	3,76	0,03
3	22	3,25	0,83	1,84	3,69	0,14	169	3,71	0,97	1,84	3,72	0,02
4	15	3,82	1,10	2,45	3,65	0,12	236	3,70	1,02	2,45	3,68	0,02
5	40	3,59	1,42	3,06	3,62	0,11	384	3,55	1,01	3,06	3,65	0,02
6	54	3,61	0,99	3,67	3,58	0,11	555	3,53	1,10	3,67	3,62	0,02
7	64	3,48	1,13	4,29	3,56	0,10	679	3,43	1,00	4,29	3,59	0,02
8	61	3,40	0,99	4,90	3,54	0,09	771	3,48	1,00	4,90	3,57	0,02
9	61	3,23	0,90	5,51	3,52	0,08	785	3,48	1,10	5,51	3,54	0,02
10	80	3,51	1,11	6,12	3,50	0,08	903	3,42	1,03	6,12	3,52	0,02
11	88	3,51	0,89	6,73	3,49	0,07	1013	3,40	1,02	6,73	3,50	0,02
12	122	3,38	1,03	7,35	3,48	0,06	1060	3,39	1,00	7,35	3,49	0,02
13	94	3,35	0,98	7,96	3,47	0,06	1179	3,40	1,04	7,96	3,47	0,02
14	113	3,34	0,98	8,57	3,46	0,05	1218	3,37	1,03	8,57	3,46	0,01
15	123	3,59	1,11	9,18	3,45	0,05	1255	3,39	1,01	9,18	3,45	0,01
16	130	3,21	0,91	9,80	3,45	0,04	1391	3,37	1,00	9,80	3,44	0,01
17	150	3,48	1,02	10,41	3,44	0,04	1389	3,35	1,05	10,41	3,43	0,01
18	153	3,41	1,06	11,02	3,44	0,04	1451	3,33	1,01	11,02	3,42	0,01
19	145	3,28	1,02	11,63	3,43	0,04	1492	3,30	1,03	11,63	3,41	0,01
20	136	3,42	1,13	12,24	3,43	0,04	1505	3,29	1,02	12,24	3,40	0,01
21	166	3,19	1,00	12,86	3,42	0,04	1462	3,27	1,03	12,86	3,40	0,01
22	163	3,27	1,08	13,47	3,42	0,04	1408	3,27	1,03	13,47	3,39	0,01
23	156	3,11	0,95	14,08	3,41	0,04	1425	3,24	1,06	14,08	3,38	0,01
24	136	3,03	0,89	14,69	3,40	0,04	1230	3,21	1,05	14,69	3,37	0,00
25	110	3,17	1,19	15,31	3,40	0,04	1052	3,16	0,98	15,31	3,37	0,00
26	101	3,00	0,81	15,92	3,39	0,04	837	3,17	0,99	15,92	3,36	0,00
27	61	3,06	0,88	16,53	3,38	0,04	694	3,19	1,01	16,53	3,35	0,00
28	72	3,10	0,94	17,14	3,36	0,04	564	3,10	0,94	17,14	3,34	0,00
29	45	3,00	0,88	17,76	3,35	0,04	465	3,11	0,98	17,76	3,33	0,00
30	25	2,97	1,03	18,37	3,33	0,04	321	3,14	1,05	18,37	3,32	0,00
				18,98	3,32	0,04				18,98	3,31	0,00
				19,59	3,30	0,03				19,59	3,30	0,00
				20,20	3,28	0,03				20,20	3,29	0,00
				20,82	3,26	0,03				20,82	3,28	0,00
				21,43	3,24	0,03				21,43	3,26	0,01
				22,04	3,22	0,03				22,04	3,25	0,01
				22,65	3,19	0,03				22,65	3,24	0,01
				23,27	3,17	0,03				23,27	3,23	0,01
				23,88	3,15	0,03				23,88	3,21	0,01
				24,49	3,12	0,03				24,49	3,20	0,01
				25,10	3,10	0,03				25,10	3,19	0,01
				25,71	3,08	0,03				25,71	3,17	0,01
				26,33	3,06	0,03				26,33	3,16	0,01
				26,94	3,04	0,03				26,94	3,15	0,01
				27,55	3,03	0,03				27,55	3,14	0,01
				28,16	3,01	0,03				28,16	3,13	0,01
				28,78	3,00	0,03				28,78	3,13	0,01
				29,39	3,00	0,04				29,39	3,12	0,02
				30,00	3,00	0,05				30,00	3,12	0,02

© 2024 Chourpiliadis C et al. *JAMA Network Open.*

Stress-related disorders												
HDL-C												
	Cases						Controls					
	Observed			Predicted			Observed			Predicted		
Time since index date	Number	Mean	SD	Time since index date	Mean	SE	Number	Mean	SD	Time since index date	Mean	SE
0	7	1,76	0,38	0,00	1,66	0,07	76	1,50	0,32	0,00	1,52	0,01
1	7	1,58	0,39	0,61	1,64	0,05	120	1,54	0,38	0,61	1,52	0,01
2	16	1,46	0,35	1,22	1,62	0,04	146	1,52	0,39	1,22	1,52	0,01
3	22	1,63	0,42	1,84	1,60	0,03	164	1,54	0,39	1,84	1,53	0,01
4	14	1,53	0,43	2,45	1,59	0,03	239	1,54	0,41	2,45	1,53	0,01
5	43	1,53	0,32	3,06	1,58	0,03	391	1,53	0,35	3,06	1,53	0,01
6	55	1,58	0,38	3,67	1,58	0,03	577	1,57	0,36	3,67	1,54	0,01
7	69	1,59	0,46	4,29	1,57	0,03	705	1,55	0,39	4,29	1,54	0,01
8	60	1,66	0,47	4,90	1,57	0,03	804	1,55	0,37	4,90	1,54	0,01
9	68	1,53	0,40	5,51	1,56	0,02	826	1,56	0,40	5,51	1,55	0,00
10	80	1,53	0,41	6,12	1,56	0,02	926	1,55	0,37	6,12	1,55	0,00
11	92	1,60	0,42	6,73	1,56	0,02	1051	1,54	0,36	6,73	1,55	0,00
12	128	1,54	0,41	7,35	1,56	0,02	1093	1,56	0,38	7,35	1,55	0,00
13	97	1,60	0,38	7,96	1,56	0,02	1232	1,58	0,38	7,96	1,55	0,00
14	114	1,55	0,35	8,57	1,56	0,02	1251	1,56	0,38	8,57	1,56	0,00
15	129	1,51	0,36	9,18	1,56	0,02	1288	1,58	0,38	9,18	1,56	0,00
16	132	1,59	0,38	9,80	1,56	0,02	1436	1,56	0,38	9,80	1,56	0,00
17	155	1,53	0,37	10,41	1,55	0,02	1414	1,56	0,37	10,41	1,56	0,00
18	152	1,57	0,38	11,02	1,55	0,01	1492	1,57	0,36	11,02	1,56	0,00
19	148	1,54	0,37	11,63	1,55	0,01	1579	1,56	0,37	11,63	1,56	0,00
20	144	1,54	0,43	12,24	1,55	0,01	1605	1,57	0,37	12,24	1,56	0,00
21	181	1,50	0,39	12,86	1,55	0,01	1513	1,57	0,37	12,86	1,56	0,00
22	166	1,55	0,35	13,47	1,55	0,01	1482	1,55	0,37	13,47	1,56	0,00
23	159	1,59	0,40	14,08	1,55	0,01	1483	1,59	0,38	14,08	1,56	0,00
24	138	1,66	0,35	14,69	1,55	0,01	1305	1,58	0,38	14,69	1,56	0,00
25	112	1,62	0,29	15,31	1,55	0,01	1094	1,58	0,38	15,31	1,56	0,00
26	100	1,66	0,36	15,92	1,55	0,01	852	1,60	0,38	15,92	1,56	0,00
27	58	1,59	0,39	16,53	1,55	0,01	684	1,64	0,39	16,53	1,56	0,00
28	70	1,71	0,33	17,14	1,55	0,01	547	1,61	0,37	17,14	1,56	0,00
29	45	1,59	0,38	17,76	1,56	0,01	452	1,60	0,34	17,76	1,56	0,00
30	25	1,62	0,31	18,37	1,56	0,01	308	1,66	0,35	18,37	1,56	0,00
				18,98	1,56	0,01				18,98	1,57	0,00
				19,59	1,56	0,01				19,59	1,57	0,00
				20,20	1,57	0,01				20,20	1,57	0,00
				20,82	1,57	0,01				20,82	1,57	0,00
				21,43	1,58	0,01				21,43	1,57	0,00
				22,04	1,58	0,01				22,04	1,57	0,00
				22,65	1,59	0,01				22,65	1,57	0,01
				23,27	1,59	0,01				23,27	1,58	0,01
				23,88	1,60	0,01				23,88	1,58	0,01
				24,49	1,61	0,02				24,49	1,58	0,01
				25,10	1,61	0,02				25,10	1,59	0,01
				25,71	1,62	0,02				25,71	1,59	0,01
				26,33	1,62	0,02				26,33	1,60	0,01
				26,94	1,63	0,02				26,94	1,60	0,01
				27,55	1,63	0,02				27,55	1,61	0,01
				28,16	1,63	0,03				28,16	1,62	0,01
				28,78	1,63	0,03				28,78	1,63	0,01
				29,39	1,63	0,04				29,39	1,64	0,01
				30,00	1,63	0,06				30,00	1,65	0,01

© 2024 Chourpiliadis C et al. *JAMA Network Open.*

Stress-related disorders												
ApoA-I												
	Cases						Controls					
	Observed			Predicted			Observed			Predicted		
Time since index date	Number	Mean	SD	Time since index date	Mean	SE	Number	Mean	SD	Time since index date	Mean	SE
0	4	1,59	0,27	0,00	1,61	0,06	61	1,40	0,18	0,00	1,42	0,01
1	5	1,64	0,19	0,61	1,59	0,04	109	1,43	0,21	0,61	1,42	0,01
2	12	1,48	0,16	1,22	1,57	0,03	137	1,44	0,23	1,22	1,42	0,00
3	20	1,52	0,24	1,84	1,54	0,03	155	1,41	0,24	1,84	1,42	0,00
4	10	1,56	0,23	2,45	1,53	0,03	210	1,41	0,23	2,45	1,42	0,00
5	38	1,38	0,20	3,06	1,51	0,02	331	1,41	0,20	3,06	1,42	0,00
6	40	1,44	0,17	3,67	1,49	0,02	448	1,42	0,21	3,67	1,42	0,00
7	47	1,46	0,29	4,29	1,48	0,02	563	1,40	0,23	4,29	1,42	0,00
8	46	1,44	0,25	4,90	1,47	0,02	647	1,41	0,21	4,90	1,42	0,00
9	53	1,40	0,23	5,51	1,46	0,02	658	1,42	0,22	5,51	1,41	0,00
10	68	1,39	0,25	6,12	1,45	0,02	742	1,40	0,21	6,12	1,41	0,00
11	64	1,42	0,23	6,73	1,44	0,02	857	1,40	0,20	6,73	1,41	0,00
12	105	1,41	0,23	7,35	1,43	0,02	897	1,40	0,22	7,35	1,41	0,00
13	78	1,47	0,23	7,96	1,43	0,01	1032	1,42	0,22	7,96	1,41	0,00
14	90	1,40	0,21	8,57	1,42	0,01	1041	1,40	0,21	8,57	1,41	0,00
15	109	1,39	0,20	9,18	1,42	0,01	1094	1,41	0,22	9,18	1,41	0,00
16	116	1,41	0,21	9,80	1,41	0,01	1194	1,41	0,22	9,80	1,41	0,00
17	136	1,39	0,20	10,41	1,41	0,01	1201	1,40	0,21	10,41	1,41	0,00
18	127	1,39	0,21	11,02	1,41	0,01	1260	1,40	0,21	11,02	1,41	0,00
19	122	1,41	0,21	11,63	1,41	0,01	1311	1,40	0,21	11,63	1,41	0,00
20	126	1,44	0,28	12,24	1,41	0,01	1324	1,40	0,21	12,24	1,41	0,00
21	160	1,39	0,22	12,86	1,41	0,01	1253	1,40	0,21	12,86	1,40	0,00
22	147	1,40	0,20	13,47	1,41	0,01	1248	1,39	0,21	13,47	1,40	0,00
23	139	1,42	0,24	14,08	1,41	0,01	1269	1,41	0,22	14,08	1,40	0,00
24	128	1,44	0,22	14,69	1,41	0,01	1163	1,42	0,22	14,69	1,40	0,00
25	105	1,43	0,19	15,31	1,41	0,01	1013	1,41	0,22	15,31	1,40	0,00
26	98	1,45	0,24	15,92	1,41	0,01	832	1,41	0,22	15,92	1,40	0,00
27	60	1,41	0,24	16,53	1,41	0,01	693	1,44	0,23	16,53	1,40	0,00
28	70	1,47	0,20	17,14	1,41	0,01	563	1,41	0,23	17,14	1,40	0,00
29	45	1,40	0,23	17,76	1,41	0,01	466	1,41	0,20	17,76	1,40	0,00
30	25	1,42	0,17	18,37	1,41	0,01	323	1,44	0,21	18,37	1,40	0,00
				18,98	1,41	0,01				18,98	1,40	0,00
				19,59	1,41	0,01				19,59	1,40	0,00
				20,20	1,42	0,01				20,20	1,40	0,00
				20,82	1,42	0,01				20,82	1,40	0,00
				21,43	1,42	0,01				21,43	1,40	0,00
				22,04	1,42	0,01				22,04	1,41	0,00
				22,65	1,42	0,01				22,65	1,41	0,00
				23,27	1,43	0,01				23,27	1,41	0,00
				23,88	1,43	0,01				23,88	1,41	0,00
				24,49	1,43	0,01				24,49	1,41	0,00
				25,10	1,43	0,01				25,10	1,41	0,00
				25,71	1,43	0,01				25,71	1,41	0,00
				26,33	1,43	0,01				26,33	1,42	0,00
				26,94	1,43	0,01				26,94	1,42	0,00
				27,55	1,43	0,01				27,55	1,42	0,01
				28,16	1,43	0,01				28,16	1,42	0,01
				28,78	1,43	0,01				28,78	1,43	0,01
				29,39	1,43	0,02				29,39	1,43	0,01
				30,00	1,42	0,02				30,00	1,43	0,01

© 2024 Chourpiliadis C et al. *JAMA Network Open.*

Stress-related disorders												
ApoB												
	Cases						Controls					
	Observed			Predicted			Observed			Predicted		
Time since index date	Number	Mean	SD	Time since index date	Mean	SE	Number	Mean	SD	Time since index date	Mean	SE
0	4	1,13	0,39	0,00	1,29	0,10	59	1,17	0,33	0,00	1,23	0,01
1	5	1,40	0,39	0,61	1,29	0,08	109	1,23	0,37	0,61	1,22	0,01
2	12	1,47	0,26	1,22	1,29	0,06	125	1,27	0,42	1,22	1,22	0,01
3	18	1,15	0,39	1,84	1,29	0,05	140	1,26	0,35	1,84	1,21	0,01
4	12	1,36	0,43	2,45	1,28	0,05	204	1,24	0,47	2,45	1,21	0,01
5	40	1,21	0,43	3,06	1,27	0,04	302	1,17	0,34	3,06	1,20	0,01
6	37	1,25	0,39	3,67	1,26	0,04	404	1,12	0,35	3,67	1,20	0,01
7	50	1,13	0,37	4,29	1,25	0,04	523	1,11	0,33	4,29	1,19	0,01
8	42	1,18	0,37	4,90	1,24	0,04	613	1,15	0,34	4,90	1,18	0,01
9	48	1,03	0,28	5,51	1,22	0,04	658	1,14	0,38	5,51	1,18	0,01
10	61	1,10	0,32	6,12	1,21	0,03	712	1,12	0,34	6,12	1,17	0,01
11	58	1,11	0,30	6,73	1,20	0,03	825	1,14	0,39	6,73	1,16	0,01
12	79	1,12	0,33	7,35	1,19	0,03	830	1,11	0,35	7,35	1,16	0,01
13	67	1,12	0,32	7,96	1,18	0,02	942	1,12	0,34	7,96	1,15	0,01
14	87	1,15	0,38	8,57	1,16	0,02	964	1,11	0,35	8,57	1,15	0,01
15	101	1,16	0,34	9,18	1,15	0,02	996	1,13	0,35	9,18	1,14	0,01
16	104	1,05	0,33	9,80	1,14	0,02	1063	1,11	0,33	9,80	1,14	0,01
17	119	1,10	0,34	10,41	1,14	0,02	1095	1,12	0,35	10,41	1,13	0,01
18	109	1,12	0,39	11,02	1,13	0,01	1106	1,11	0,35	11,02	1,13	0,01
19	111	1,09	0,35	11,63	1,12	0,01	1170	1,09	0,34	11,63	1,12	0,01
20	123	1,11	0,36	12,24	1,12	0,01	1265	1,09	0,34	12,24	1,12	0,00
21	156	1,06	0,37	12,86	1,11	0,01	1139	1,09	0,34	12,86	1,12	0,00
22	133	1,11	0,38	13,47	1,11	0,01	1119	1,09	0,35	13,47	1,11	0,00
23	119	1,06	0,34	14,08	1,10	0,01	1143	1,08	0,34	14,08	1,11	0,00
24	115	1,06	0,31	14,69	1,10	0,01	984	1,07	0,33	14,69	1,11	0,00
25	92	1,10	0,38	15,31	1,10	0,01	838	1,09	0,33	15,31	1,11	0,00
26	82	1,03	0,24	15,92	1,09	0,01	675	1,10	0,31	15,92	1,10	0,00
27	49	1,04	0,30	16,53	1,09	0,01	507	1,12	0,32	16,53	1,10	0,00
28	44	1,11	0,26	17,14	1,09	0,01	401	1,11	0,30	17,14	1,10	0,00
29	31	1,09	0,26	17,76	1,09	0,01	342	1,15	0,30	17,76	1,10	0,00
30	18	1,09	0,27	18,37	1,09	0,01	239	1,17	0,32	18,37	1,09	0,00
				18,98	1,09	0,01				18,98	1,09	0,00
				19,59	1,09	0,01				19,59	1,09	0,00
				20,20	1,08	0,01				20,20	1,09	0,00
				20,82	1,08	0,01				20,82	1,09	0,00
				21,43	1,08	0,01				21,43	1,09	0,00
				22,04	1,08	0,01				22,04	1,09	0,00
				22,65	1,08	0,01				22,65	1,09	0,00
				23,27	1,08	0,01				23,27	1,09	0,00
				23,88	1,08	0,01				23,88	1,09	0,00
				24,49	1,08	0,01				24,49	1,09	0,00
				25,10	1,08	0,01				25,10	1,09	0,00
				25,71	1,08	0,01				25,71	1,10	0,00
				26,33	1,08	0,01				26,33	1,10	0,00
				26,94	1,08	0,01				26,94	1,11	0,00
				27,55	1,08	0,01				27,55	1,12	0,01
				28,16	1,08	0,01				28,16	1,13	0,01
				28,78	1,08	0,02				28,78	1,14	0,01
				29,39	1,08	0,02				29,39	1,15	0,01
				30,00	1,09	0,03				30,00	1,17	0,01

© 2024 Chourpiliadis C et al. *JAMA Network Open.*

Stress-related disorders												
Log2 LDL-C/HDL-C ratio												
	Cases						Controls					
	Observed			Predicted			Observed			Predicted		
Time since index date	Number	Mean	SD	Time since index date	Mean	SE	Number	Mean	SD	Time since index date	Mean	SE
0	7	1,14	0,69	0,00	1,20	0,15	70	1,32	0,54	0,00	1,31	0,02
1	7	1,14	0,32	0,61	1,19	0,11	111	1,25	0,61	0,61	1,29	0,01
2	15	1,38	0,50	1,22	1,18	0,09	137	1,24	0,57	1,22	1,28	0,01
3	21	1,00	0,65	1,84	1,17	0,07	155	1,27	0,56	1,84	1,26	0,01
4	14	1,30	0,67	2,45	1,16	0,06	226	1,25	0,58	2,45	1,24	0,01
5	37	1,15	0,69	3,06	1,16	0,06	369	1,19	0,57	3,06	1,23	0,01
6	51	1,14	0,55	3,67	1,15	0,06	536	1,14	0,57	3,67	1,22	0,01
7	63	1,11	0,63	4,29	1,14	0,05	654	1,12	0,61	4,29	1,20	0,01
8	59	1,00	0,69	4,90	1,14	0,05	743	1,14	0,59	4,90	1,19	0,01
9	60	1,06	0,62	5,51	1,13	0,04	757	1,13	0,66	5,51	1,18	0,01
10	76	1,16	0,58	6,12	1,13	0,04	872	1,11	0,62	6,12	1,17	0,01
11	86	1,16	0,58	6,73	1,13	0,04	972	1,10	0,62	6,73	1,16	0,01
12	119	1,12	0,64	7,35	1,12	0,03	1007	1,09	0,59	7,35	1,15	0,01
13	90	1,03	0,58	7,96	1,12	0,03	1142	1,07	0,62	7,96	1,14	0,01
14	110	1,07	0,60	8,57	1,12	0,03	1165	1,07	0,63	8,57	1,13	0,01
15	119	1,21	0,58	9,18	1,12	0,02	1208	1,07	0,62	9,18	1,12	0,01
16	125	1,00	0,59	9,80	1,12	0,02	1346	1,07	0,62	9,80	1,11	0,01
17	145	1,17	0,59	10,41	1,12	0,02	1336	1,06	0,61	10,41	1,11	0,01
18	145	1,09	0,60	11,02	1,12	0,02	1401	1,05	0,59	11,02	1,10	0,00
19	142	1,06	0,60	11,63	1,12	0,02	1453	1,05	0,61	11,63	1,10	0,00
20	130	1,13	0,67	12,24	1,12	0,02	1473	1,03	0,61	12,24	1,09	0,00
21	162	1,07	0,66	12,86	1,12	0,02	1415	1,02	0,62	12,86	1,09	0,00
22	157	1,04	0,61	13,47	1,12	0,03	1365	1,03	0,61	13,47	1,08	0,00
23	151	0,94	0,57	14,08	1,12	0,03	1385	0,99	0,64	14,08	1,08	0,00
24	132	0,83	0,54	14,69	1,11	0,03	1205	0,98	0,65	14,69	1,07	0,00
25	107	0,89	0,59	15,31	1,11	0,03	1020	0,96	0,62	15,31	1,07	0,00
26	96	0,82	0,52	15,92	1,10	0,03	810	0,95	0,61	15,92	1,06	0,00
27	57	0,87	0,53	16,53	1,10	0,03	675	0,91	0,63	16,53	1,06	0,00
28	70	0,83	0,52	17,14	1,09	0,02	547	0,91	0,62	17,14	1,06	0,00
29	45	0,89	0,58	17,76	1,08	0,02	452	0,91	0,58	17,76	1,05	0,00
30	25	0,82	0,58	18,37	1,07	0,02	308	0,85	0,60	18,37	1,05	0,00
				18,98	1,06	0,02				18,98	1,04	0,00
				19,59	1,05	0,02				19,59	1,04	0,00
				20,20	1,03	0,02				20,20	1,03	0,00
				20,82	1,02	0,02				20,82	1,02	0,00
				21,43	1,00	0,02				21,43	1,02	0,00
				22,04	0,99	0,02				22,04	1,01	0,00
				22,65	0,97	0,02				22,65	1,00	0,00
				23,27	0,95	0,02				23,27	0,99	0,00
				23,88	0,93	0,02				23,88	0,98	0,00
				24,49	0,92	0,02				24,49	0,97	0,00
				25,10	0,90	0,02				25,10	0,96	0,00
				25,71	0,88	0,02				25,71	0,95	0,00
				26,33	0,87	0,02				26,33	0,94	0,00
				26,94	0,85	0,02				26,94	0,93	0,00
				27,55	0,84	0,02				27,55	0,92	0,01
				28,16	0,84	0,02				28,16	0,90	0,01
				28,78	0,83	0,02				28,78	0,89	0,01
				29,39	0,83	0,03				29,39	0,88	0,01
				30,00	0,84	0,03				30,00	0,86	0,01

© 2024 Chourpiliadis C et al. *JAMA Network Open.*

Stress-related disorders												
Log2 ApoB/ApoA-I ratio												
	Cases						Controls					
	Observed			Predicted			Observed			Predicted		
Time since index date	Number	Mean	SD	Time since index date	Mean	SE	Number	Mean	SD	Time since index date	Mean	SE
0	11	-0,47	0,00	0,00	-0,36	0,19	212	-0,34	0,35	0,00	-0,27	0,03
1	18	-0,43	0,22	0,61	-0,35	0,14	389	-0,28	0,43	0,61	-0,28	0,03
2	20	0,01	0,16	1,22	-0,35	0,11	374	-0,25	0,49	1,22	-0,28	0,02
3	39	-0,43	0,49	1,84	-0,35	0,09	421	-0,18	0,47	1,84	-0,29	0,02
4	16	-0,38	0,16	2,45	-0,35	0,08	471	-0,32	0,47	2,45	-0,30	0,02
5	89	-0,26	0,35	3,06	-0,35	0,07	781	-0,33	0,45	3,06	-0,30	0,02
6	90	-0,34	0,39	3,67	-0,36	0,07	877	-0,36	0,48	3,67	-0,31	0,02
7	121	-0,36	0,41	4,29	-0,36	0,06	1096	-0,41	0,46	4,29	-0,32	0,02
8	83	-0,41	0,50	4,90	-0,36	0,06	1313	-0,33	0,51	4,90	-0,32	0,02
9	116	-0,64	0,38	5,51	-0,36	0,06	1468	-0,40	0,53	5,51	-0,33	0,02
10	124	-0,38	0,39	6,12	-0,36	0,05	1580	-0,37	0,51	6,12	-0,34	0,02
11	97	-0,40	0,53	6,73	-0,37	0,05	1952	-0,36	0,50	6,73	-0,34	0,01
12	117	-0,32	0,41	7,35	-0,37	0,04	1830	-0,43	0,48	7,35	-0,35	0,01
13	110	-0,36	0,46	7,96	-0,37	0,04	2039	-0,43	0,50	7,96	-0,35	0,01
14	195	-0,39	0,46	8,57	-0,37	0,04	2109	-0,37	0,51	8,57	-0,36	0,01
15	231	-0,43	0,48	9,18	-0,37	0,03	2229	-0,36	0,52	9,18	-0,36	0,01
16	227	-0,40	0,44	9,80	-0,37	0,03	2348	-0,36	0,49	9,80	-0,37	0,01
17	246	-0,25	0,51	10,41	-0,37	0,03	2377	-0,37	0,49	10,41	-0,37	0,01
18	259	-0,30	0,61	11,02	-0,37	0,03	2442	-0,40	0,51	11,02	-0,38	0,01
19	219	-0,47	0,55	11,63	-0,37	0,03	2628	-0,42	0,47	11,63	-0,38	0,01
20	234	-0,46	0,61	12,24	-0,37	0,03	2737	-0,42	0,48	12,24	-0,38	0,01
21	304	-0,48	0,63	12,86	-0,37	0,03	2239	-0,45	0,46	12,86	-0,39	0,01
22	227	-0,43	0,47	13,47	-0,37	0,03	2335	-0,39	0,51	13,47	-0,39	0,01
23	166	-0,55	0,45	14,08	-0,38	0,03	2236	-0,39	0,54	14,08	-0,39	0,01
24	223	-0,54	0,41	14,69	-0,38	0,03	1992	-0,44	0,50	14,69	-0,39	0,01
25	202	-0,41	0,51	15,31	-0,38	0,03	1625	-0,44	0,49	15,31	-0,40	0,01
26	155	-0,54	0,27	15,92	-0,39	0,03	1305	-0,41	0,47	15,92	-0,40	0,01
27	76	-0,55	0,47	16,53	-0,39	0,03	913	-0,43	0,51	16,53	-0,40	0,01
28	58	-0,49	0,32	17,14	-0,40	0,03	728	-0,37	0,46	17,14	-0,40	0,01
29	47	-0,61	0,23	17,76	-0,41	0,02	495	-0,32	0,41	17,76	-0,41	0,01
30	30	-0,19	0,31	18,37	-0,42	0,02	421	-0,33	0,39	18,37	-0,41	0,01
				18,98	-0,43	0,02				18,98	-0,41	0,01
				19,59	-0,44	0,02				19,59	-0,41	0,01
				20,20	-0,45	0,02				20,20	-0,42	0,01
				20,82	-0,46	0,02				20,82	-0,42	0,01
				21,43	-0,47	0,02				21,43	-0,42	0,01
				22,04	-0,48	0,02				22,04	-0,42	0,01
				22,65	-0,50	0,02				22,65	-0,42	0,01
				23,27	-0,51	0,02				23,27	-0,42	0,01
				23,88	-0,51	0,03				23,88	-0,42	0,01
				24,49	-0,52	0,03				24,49	-0,42	0,01
				25,10	-0,52	0,03				25,10	-0,42	0,01
				25,71	-0,52	0,03				25,71	-0,41	0,01
				26,33	-0,52	0,03				26,33	-0,41	0,01
				26,94	-0,51	0,03				26,94	-0,40	0,01
				27,55	-0,49	0,03				27,55	-0,39	0,01
				28,16	-0,47	0,04				28,16	-0,37	0,01
				28,78	-0,44	0,04				28,78	-0,36	0,01
				29,39	-0,40	0,05				29,39	-0,34	0,01
				30,00	-0,34	0,07				30,00	-0,32	0,02

© 2024 Chourpiliadis C et al. *JAMA Network Open.*

eFigure 1. Flowchart of the Study Design

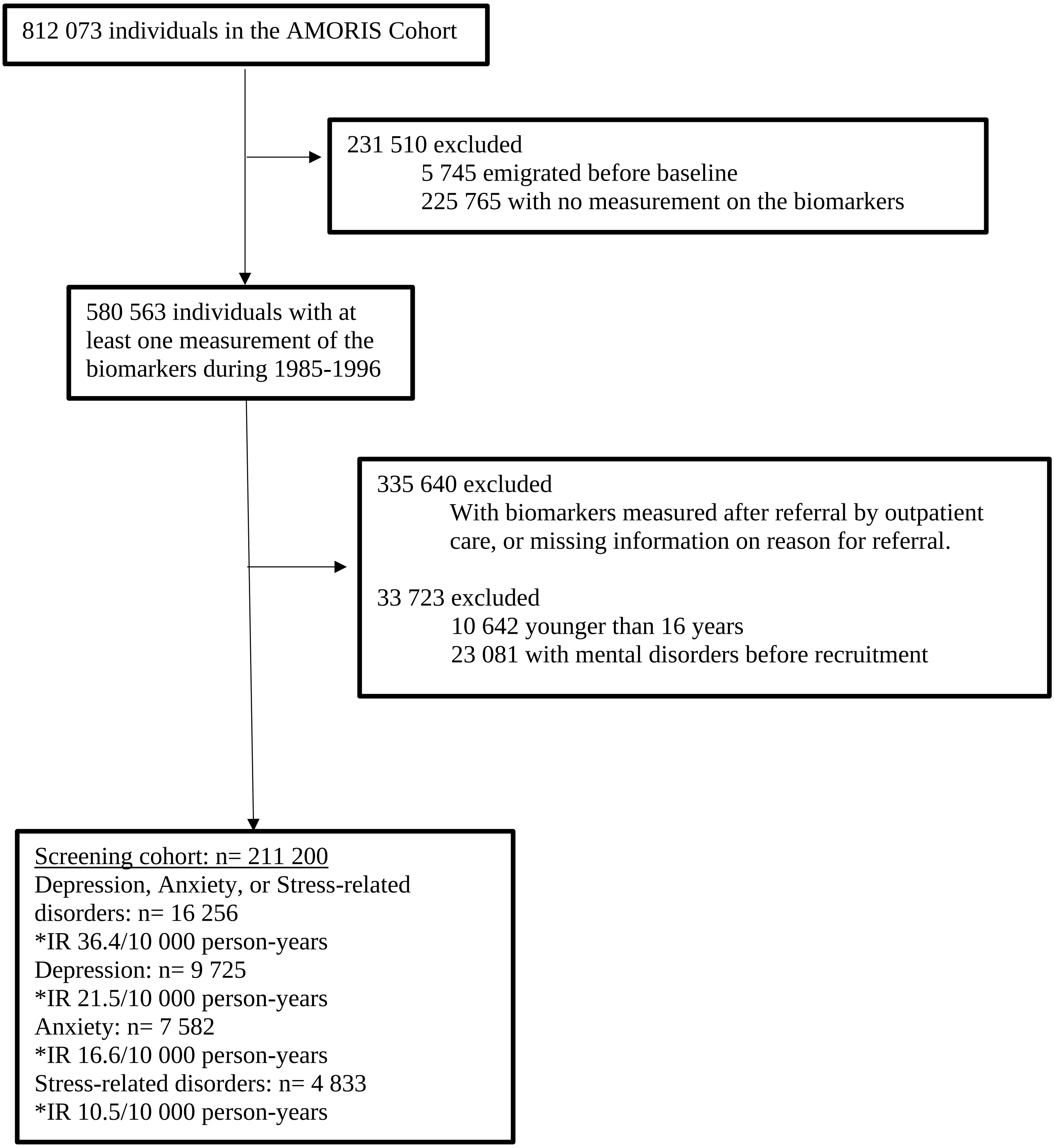

© 2024 Chourpiliadis C et al. *JAMA Network Open.*

eFigure 2. Mean Concentrations of Blood Biomarkers of Lipid, Carbohydrate, and Apolipoprotein Metabolism During the 30 Years Before the Diagnosis of Depression, Anxiety, or Stress-Related Disorders, Comparing Patients With Such Disorders (Green Area) to the Matched Controls (Pink Area)

The method of local polynomial smoothing with 4th-degree polynomial function and Gaussian kernel function was used to plot the mean concentrations of the biomarkers over time before the index date with 95% CI. TC: total cholesterol; TG: triglycerides; LDL-C: low-density lipoprotein cholesterol; HDL-C: high-density lipoprotein cholesterol; ApoA-I: apolipoprotein A-I; ApoB: apolipoprotein B

© 2024 Chourpiliadis C et al. *JAMA Network Open.*

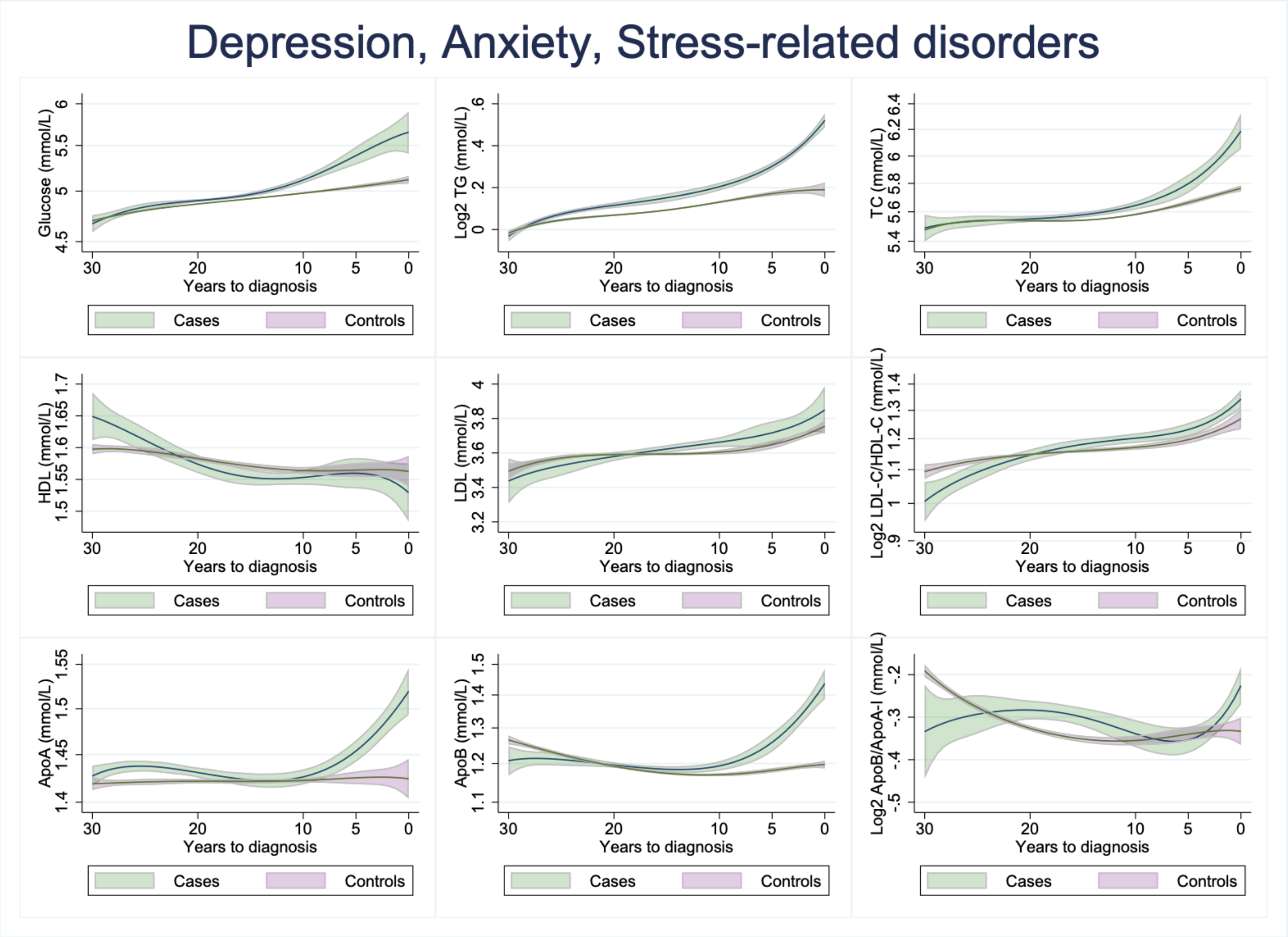

© 2024 Chourpiliadis C et al. *JAMA Network Open.*

Depression

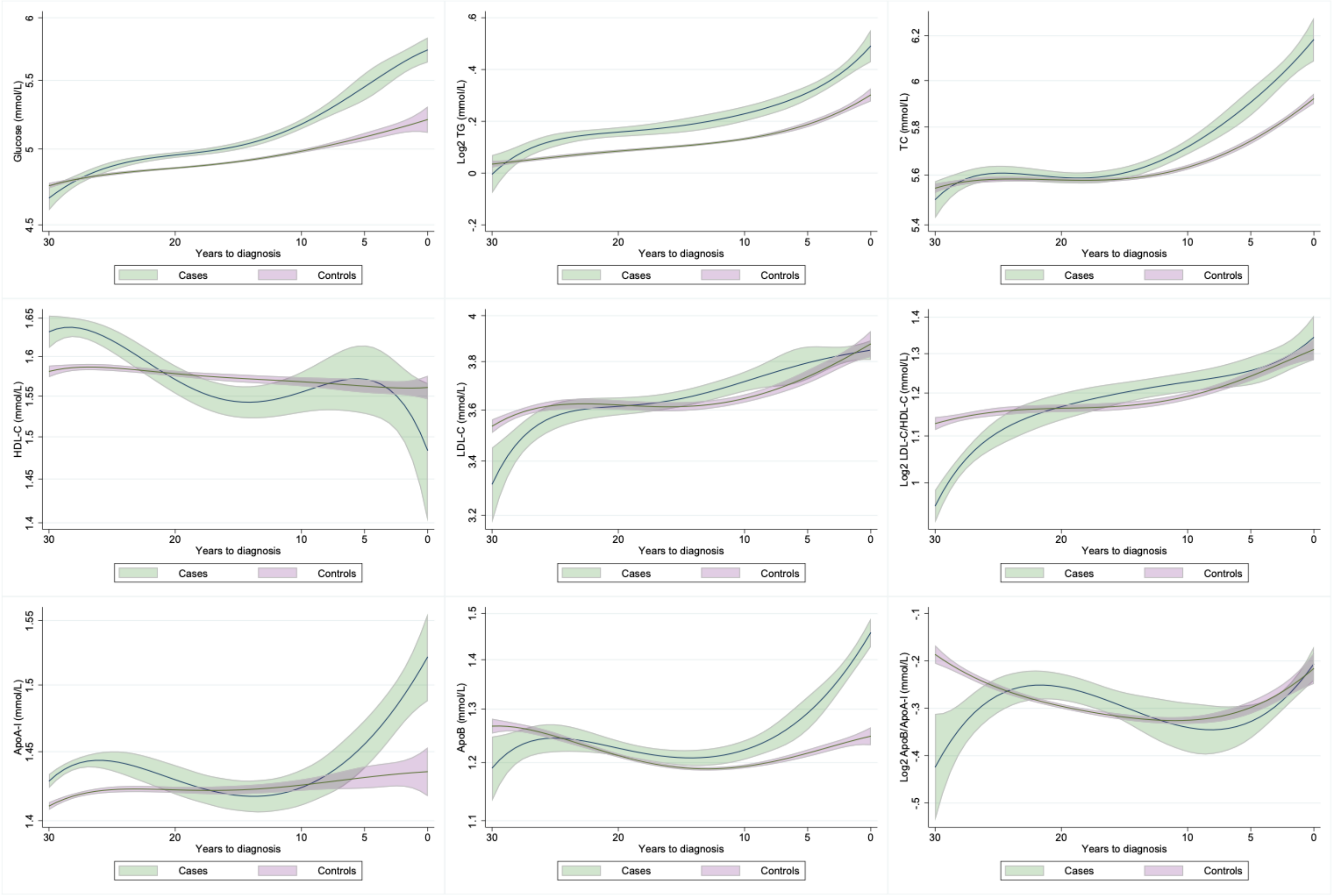

© 2024 Chourpiliadis C et al. *JAMA Network Open.*

Anxiety

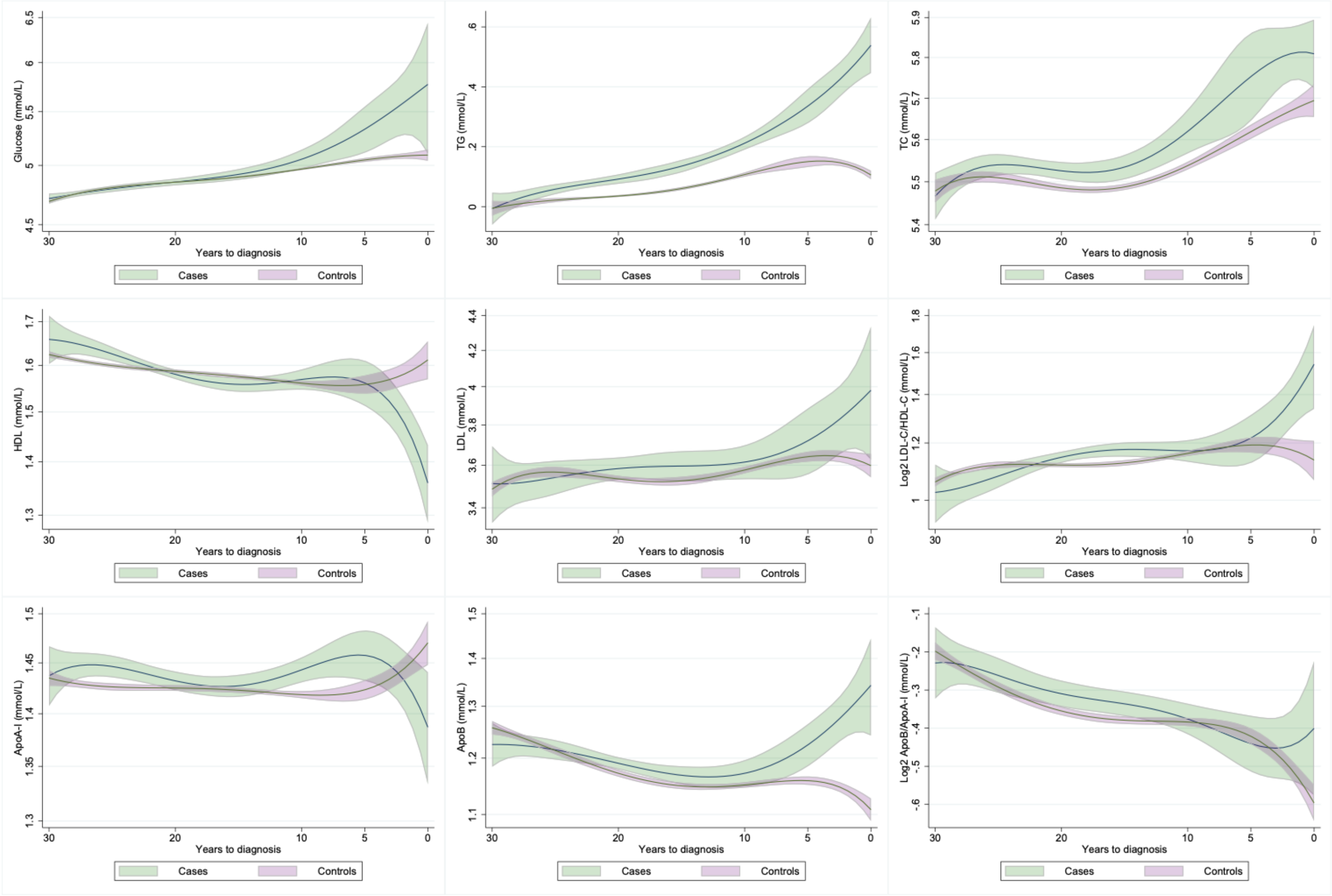

© 2024 Chourpiliadis C et al. *JAMA Network Open.*

Stress- related Disorders

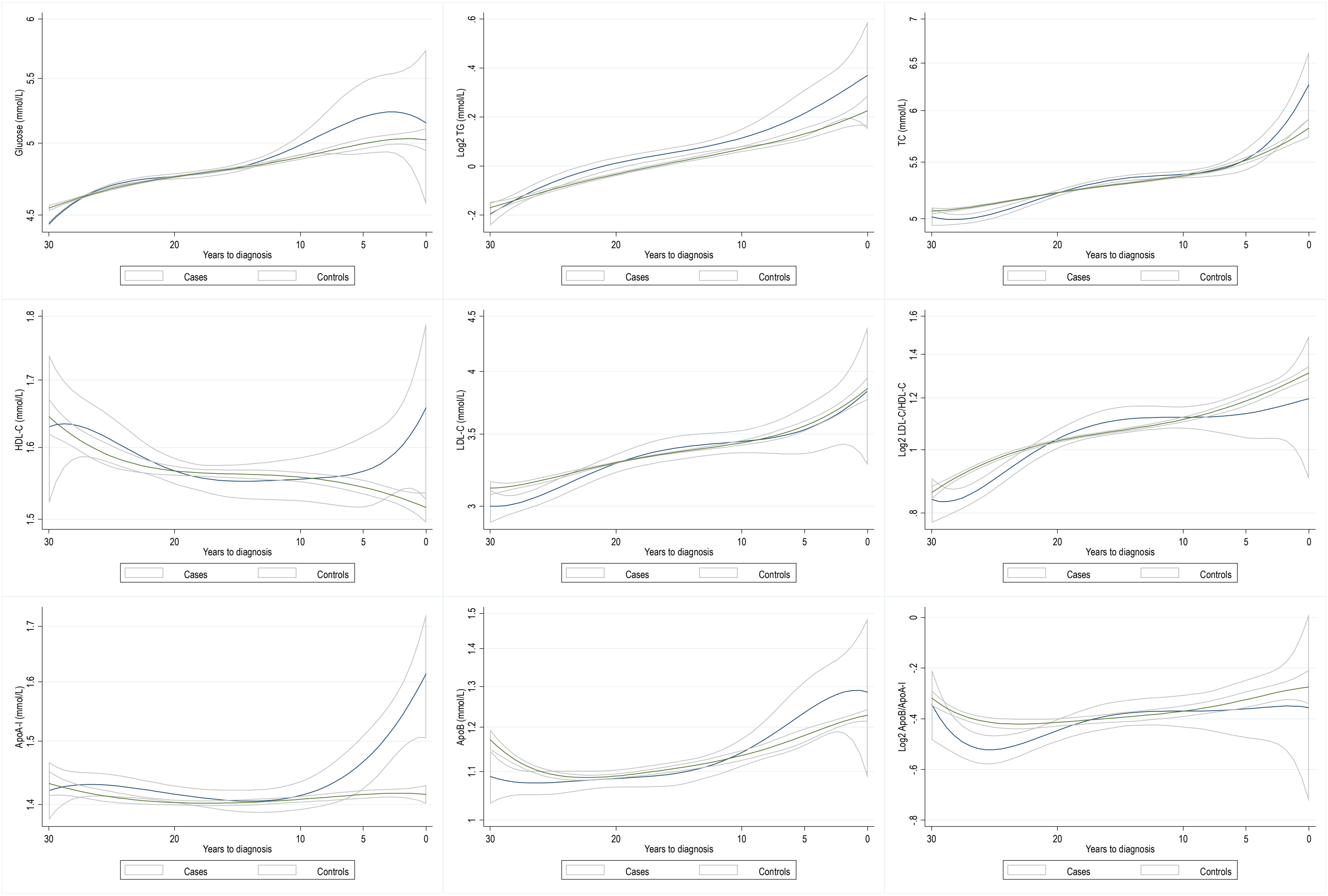

© 2024 Chourpiliadis C et al. *JAMA Network Open.*
