# Supplementary material for: Metabolic Profile and Long-Term Risk of Depression, Anxiety, and Stress-Related Disorders
Source: JAMA Netw Open. 2024 Apr 2;7(4):e244525. doi: 10.1001/jamanetworkopen.2024.4525 (PMC10988352; doi:10.1001/jamanetworkopen.2024.4525)
Supplement: Supplement 2. — Data Sharing Statement [file jamanetwopen-e244525-s002.pdf]

Data Sharing Statement

Chourpiliadis. Metabolic Profile and Long-Term Risk of Depression, Anxiety, and Stress-Related Disorders. *JAMA Netw Open*. Published April 02, 2024.
doi:10.1001/jamanetworkopen.2024.4525

Data
Data available: No
